# Impact of patient involvement on clinical practice guideline development: a parallel group study

**DOI:** 10.1186/s13012-018-0745-6

**Published:** 2018-04-16

**Authors:** Melissa J. Armstrong, C. Daniel Mullins, Gary S. Gronseth, Anna R. Gagliardi

**Affiliations:** 1 Department of Neurology, University of Florida College of Medicine, Gainesville, FL USA; 2 Pharmaceutical Health Services Research Department, University of Maryland School of Pharmacy, Baltimore, MD USA; 3 Department of Neurology, University of Kansas Medical Center, Kansas City, KS USA; 4 Toronto General Research Institute, University Health Network, Toronto, ON Canada

**Keywords:** Guidelines as Topic [MeSH term], Clinical practice guideline, Evidence-based guideline, Patient Participation [MeSH term], Patient-Centered Care [MeSH term], Patient involvement, Guideline Adherence [MeSH term], Implementation

## Abstract

**Background:**

Patient and public involvement (PPI) is recognized as a key component of clinical practice guideline development with important implications for guideline implementability. The impact of PPI on guidelines, however, has not been rigorously assessed. Better understanding of the impact of PPI must start with guideline question formation, which drives all subsequent development steps. The aim of this study was to investigate the effect of PPI on guideline question formation and validate a conceptual model of patient and public contributions to guidelines.

**Methods:**

For development of a clinical practice guideline on the topic of using amyloid positron emission tomography in the diagnosis of dementia, we convened two parallel guideline development groups, one with and one without patient representatives. Participating physicians were randomized to group assignment. Each group developed Population, Intervention, Comparator, Outcome, Time (PICOT) questions and identified key benefits and harms to incorporate in guideline development. Analysis included a descriptive comparison of proposed PICOT questions, benefits, and harms between groups and a qualitative analysis of discussion themes from audio recordings of the question development retreats.

**Results:**

Proposed guideline questions, benefits, and harms were largely similar between groups, but only the experimental group proposed outcomes relating to development of cognitive impairment at specific time points and rate of progression. The qualitative analysis of the discussions occurring during guideline question development demonstrated key differences in group conduct and validated the proposed conceptual model of patient and public contributions to guidelines. PPI influenced the conduct of guideline development, scope, inclusion of patient-relevant topics, outcome selection, and planned approaches to recommendation development, implementation, and dissemination with implications for both guideline developers and the guideline development process.

**Conclusions:**

Evidence of how PPI impacts guideline development underscores the importance of engaging patient stakeholders in guideline development and highlights developer- and guideline-specific outcomes of PPI, both of which have implications for guideline implementation. It also raises the question of whether guidelines developed without such input are acceptable for use. PPI should be considered an essential element of trustworthy guideline development for purposes of development and funding.

**Electronic supplementary material:**

The online version of this article (10.1186/s13012-018-0745-6) contains supplementary material, which is available to authorized users.

## Background

Clinical practice guidelines provide recommendations aimed at optimizing patient care and outcomes (at the individual or system level) based on a systematic literature review and assessment of benefits and harms [1, 2]. Guidelines are foundational to health care improvement efforts [3], but compliance with guidelines is variable and often poor [4–6]. When guidelines are implemented, they can improve outcomes and reduce resource utilization [7, 8], but important gaps remain in understanding optimal implementation strategies [9, 10]. There are many identified barriers to guideline implementation [11, 12]. These include guideline-related factors such as lack of confidence in development (credibility) [11, 13], stakeholder involvement (i.e., use of experts alone) [13], utility/applicability of the guideline in general [12, 13], applicability to individual patients [11, 13], and physician judgment about the balance of benefits and risks [11]. Research suggests that guidelines are more implementable when they address patient needs and preferences and include information to support patient involvement in decision-making [14] and that stakeholder involvement is a key domain impacting implementability [13]. Improving guideline implementation must thus start with involving key stakeholders—including patients—and developing guideline questions that result in relevant and applicable recommendations addressing patient preferences and needs.

In part for these reasons, patient and public involvement (PPI) is internationally recognized as an important component of guideline development. Numerous organizations recommend or require that guideline development panels include patients, patient representatives, or health consumers, including the Guidelines International Network [15], the United States’ Institute of Medicine (IOM, now renamed the National Academy of Medicine) [1], and the United Kingdom’s National Institute for Health and Care Excellence [16]. The Appraisal of Guidelines for Research and Evaluation II instrument requires that guideline developers seek the views of the target population [2]. PPI in guidelines is advocated because it recognizes that patients are experts, respects the rights of citizens in health policy development, empowers and informs consumers in health care decisions, and leads to the development of more patient-centered and trustworthy guidelines [17].

It is largely unknown, however, how PPI influences guidelines. Frameworks describe *mechanisms* for engaging patients and the public in guidelines [17, 18] and research [19, 20], but no identified framework describes *contributions* PPI makes to guidelines or anticipated outcomes. The Patient-Centered Outcomes Research Institute’s (PCORI’s) conceptual model of patient-centered outcomes research [21] outlines near-term, intermediate, and long-term outcomes of patient engagement:

- Near-term: patient-centered culture and meaningful and effective partnerships
- Intermediate: research relevant to patients and other stakeholders, questions and outcomes meaningful to end-users, use of research results in health decisions, quality health decisions, and satisfaction with health care experiences
- Long-term: optimal health

When considered within this model, investigations of PPI in guidelines—largely guideline developers’ reflections on PPI experiences and analyses of participant feedback—suggest a framework for PPI contributions to guideline development (Table 1, Additional file 1) [16, 22–33], but one that requires further investigation and validation.

**Table 1:** Conceptual model of patient and public contributions to guidelines

PCORI conceptual model—relevant outcomes	PPI guideline contribution	Select examples from literature*
1. Culture of patient-centeredness	1.1 Shaping how discussions are conducted	Patients/carers brought “common sense to bear” and reminded guideline development group to speak in patient-centered terms (Jarrett [16]) Patient participation helped keep guideline development patient-focused (van der Ham [33])
	1.2 Setting patient-centered scope	Patient representatives elaborated on content and scope of guideline topics, particularly relating to lifestyle and psychological impact of tests, etc. (Tong [22]) Participants emphasized need for patient involvement in topic selection (Brouwers [25])
	1.3 Describing personal impact of disease	Patient/carer presence constant reminder of experience of disease (Jarrett [16]) Patient involvement helped give “lived experiences” a more central role in guideline development (van der Ham [33])
	1.4 Impacting how professional team members view PPI	Experience with stakeholder involvement informed future attitudes; consensus that end-user involvement was worthwhile after initial uncertainty (Coon [24])
2. Meaningful and effective partnerships	2. Meaningful and effective partnerships	Patient’s participation in guideline development led her organization to discuss how to provide robust input into guideline development and resulted in developing an implementation plan which included a role for patient organizations (van der Ham [33])
3. Research relevant to patients/stakeholders (including questions, outcomes)	3.1 Identifying issues that may be overlooked by medical professionals	*Mentioning patient-relevant symptoms or issues not recognized by professionals* Focus groups identified issue not in the literature (lack of anesthesia use when self-harm wounds are stitched) (Cowl [31]) Patient input on mental illness guideline emphasized unique topics including vocational limitations, workplace needs, and employment support (van der Ham [33]) Infertile couples mentioned 8 issues not described by professionals, most relating to patient-centered aspects of care (e.g., taboos, lack of support after treatment) (Den Breejen [27]) *Importance of non-pharmacologic and holistic approaches* Reminding that medication is not always an appropriate treatment, prompting inclusion of non-traditional therapies (e.g., aromatherapy) in guideline (Jarrett [16]) Prompting a holistic approach including psychological and bereavement support (Jarrett [16]) *Emphasizing importance of role of relatives* Importance of role of relatives (Jarrett [16])
	3.2 Helping select patient-relevant topics and outcomes	Patient representatives help “feed in” patient and carer issues when developing clinical questions (Graham [28]) PPI resulted in additional guideline subtopic (Tong [22]) Patients helped define key questions, particularly relating to side effects (Díaz del Campo [23]) Patient/carer involvement prompted selection of patient-relevant outcomes (e.g., satisfaction) (Jarrett [16])
	3.3 Influencing guideline structure/development	*Impacting guideline structure/approach* Patient/carer engagement prompted guideline section on users’ perspective of treatment (Jarrett [16]) PPI resulted in additional guideline chapters on patient issues and on social and psychosocial issues (Graham [28]) *Participating in systematic review* Patients involved in synthesizing knowledge, forming recommendations, revising drafts (Légaré [29]) Patient representatives helped incorporate evidence from gray literature (van der Ham [33]) *Influencing recommendation development* Patients/carers influenced multiple recommendations; chair felt it was important to test recommendations against patients (Jarrett [16]) Patient representatives ensure patient/carer views are incorporated into recommendations (Graham [28]) Patient preferences provided context for recommendations (Díaz del Campo [23]) *PPI in recommendation development can identify whether the problem is a priority, inform meaningful effects, weigh risks and benefits, and assess impact of costs, acceptability, and feasibility (Alonso-Coello* [32]*, EtD 2)* *Influencing language used in guideline* Patient/carer involvement made guideline language more patient-friendly (Jarrett [16]) Patients helped make sure the guideline used widely understood terminology (Légaré [29])
4. Use of results in health decisions	4. Facilitating guideline dissemination and implementation	*4.1 Prompting inclusion of education, support for patients/carers* Patient/carer involvement prompted inclusion of information and advocacy and support for patients and carers (Jarrett [16]) Patient discussions informed implementation interventions to address workplace stigma (van der Ham [33]) *4.2 Contributing to patient guideline versions* Patients informed appropriate vocabulary and relevant content for patient guideline versions (Díaz del Campo [23]) Participants described an important role in creating a patient version of the guideline (Brouwers [25]) *4.3 Encouraging shared decision-making* Patients encouraged patients and health care professionals to partner to make decisions (Légaré [29]) *4.4 Guiding regarding end-user uptake* Patients/carers provided guidance on how local services should be involved (Jarrett [16]) *4.5 Actively disseminating* Patient associations participated in guideline development then incorporated guidelines in educational activities and conferences (Díaz del Campo [23]) End users helped plan charity event with discussion of findings (Coon [24])

Policy or development publications (rather than research) are presented in italics

*PPI* patient and public involvement

*Additional examples are provided in Additional file 1

Even if arguing for PPI on ethical and societal grounds rather than on anticipated outcomes, understanding the effect of PPI on guidelines is important in order to understand the guideline development steps at which engagement is critical, whether guideline developers are missing key questions by excluding patients/consumers, and whether PPI results in more patient-relevant outcomes and guidelines that are more acceptable to end-users and the public, all of which impact implementability. This study thus aimed to (1) investigate the effect of PPI on guideline question formation and (2) validate the conceptual model of patient and public contributions to guidelines.

## Methods

### Design

A pragmatic parallel group study design was chosen to investigate the impact of PPI on guideline question formation. The question development step was selected because it is one of the critical first steps in guideline development, it is a step for which PPI is likely to have an important role, and the impact of PPI on later steps may depend on early engagement. The study compared the conduct and proposed questions for two guideline development groups, one including patient representatives (experimental group) and one involving physicians alone (control group).

The guideline chosen for the study—the use of amyloid positron emission tomography (PET) imaging in patients with or at risk for dementia—was selected from the American Academy of Neurology (AAN) guideline subcommittee’s waiting list of nominated projects [34]. The only prior amyloid PET guideline was developed by expert consensus [35], not an IOM-compliant evidence-based process, and two more recent policy statements [36, 37] are similarly consensus-based. This guideline topic is well suited to investigate the impact of PPI as there are strong patient, advocate, and public opinions regarding the diagnosis of Alzheimer’s disease (AD) and biomarker testing, amyloid PET tracers are approved by the Food and Drug Administration but not covered by the Centers for Medicare and Medicaid Services [38], and research shows that patients want amyloid testing results even when implications are uncertain [39, 40].

The University of Florida Institutional Review Board provided approval for this study (IRB201501210).

### Recruitment

Each guideline development group included eight participants with different types of experience, balancing diverse views and manageability. Facilitators with prior experience in leading guideline development groups were invited to chair the two groups. As per usual AAN practice, an open invitation was offered at a guideline subcommittee meeting to engage subcommittee member participants. All guideline subcommittee members provided guideline development expertise; a subset also had content expertise. Additional content experts were recruited by their reputation in the field and recommendations from colleagues and/or prior AAN interactions. Patient, caregiver, and advocacy participants (“patient representatives”) were recruited through the Alzheimer’s Association’s Minnesota-North Dakota chapter. The US-based AAN takes a patient/stakeholder approach to PPI rather than seeking public/consumer representatives. All participants reviewed the IRB-approved consent document and verbally agreed to participate and have sessions audio-recorded. The study was operated under a waiver of documentation of informed consent (i.e., no signature was required). Participants were reimbursed for expenses related to meeting attendance but not otherwise compensated.

### Population and randomization

Each guideline development group included one facilitator from the guideline subcommittee with experience in moderating but not content expertise, one dementia content expert without guideline experience, one content expert with amyloid PET experience, and 1–2 guideline subcommittee members with dementia experience. The four non-physician participants in the experimental group were offset by guideline subcommittee members without content expertise in the control group to maintain consistent group size with similar degrees of physician content expertise.

Professional participants were randomized to group assignment after training at the in-person guideline retreat in order to avoid bias that might result from specific physician assignments. Randomization was stratified according to role (facilitator, dementia expert, imaging expert). An AAN staff member typed group assignment on identical cards which were double-enveloped in opaque envelopes labeled with respective roles. A separate staff member confirmed that group assignment could not be determined by looking at the envelopes. Participants selected an envelope corresponding to their role for group assignment.

### Data collection

Data was collected at a day-long in-person guideline question retreat at AAN headquarters in Minneapolis, MN, on July 15, 2016. Based on pilot study results [26], all participants received identical three-page pre-reading describing the topic and guideline development basics. At the retreat, participants jointly received training regarding the topic and guideline development. The pre-reading and in-person training were pilot-tested with University of Florida Citizen Scientist program participants.

After randomization, the groups split to meet in separate but identical conference rooms, one floor apart. The groups were tasked with developing the questions for the amyloid PET guideline using the PICOT (Population, Intervention, Comparator, Outcome, Time) format [30], identifying relevant benefits and harms, and crafting patient-language versions of proposed questions. The PICOT framework is the format used to develop answerable clinical question and inform search concepts and systematic review analysis; this framework is used for all AAN guideline questions [34]. Anticipated benefits and harms of amyloid PET use were collected separately as these inform recommendation development [1, 2] and may be distinct from diagnostic accuracy guideline question outcomes.

Data collected included meeting length, number of proposed questions, details of the PICOT categories for each question, and anticipated benefits and harms of testing. Session recordings were professionally transcribed verbatim using one recording and confirmed using a second. The principal investigator (MJA) added a blinded code for each speaker and confirmed removal of identifying information.

### Analysis

The analysis consisted of three components: (1) a descriptive analysis of meeting characteristics (e.g., length) and (2) retreat deliverables (e.g., PICOT questions) and (3) qualitative analysis of transcribed discussions. Investigators were not blind to group results. A qualitative descriptive approach [41] was used to identify, define, and organize themes from retreat discussions using NVivo 11 Pro and Microsoft Word tables. One investigator (MJA) with an interest in guideline methodology and patient engagement independently analyzed retreat discussions to create a log of codes reflecting emerging themes and sample quotes illustrating theme coding (open coding using an inductive approach). These were reviewed and discussed with a second investigator (ARG) to achieve consensus on emerging themes and to expand or merge thematic codes (axial coding). Similarities and differences in themes between groups were compared. Consolidated criteria for reporting qualitative research [42] guided the reporting of study findings (Additional file 2). Coding supporting article conclusions is included within the article and Additional file 3.

### Conceptual model

Given the lack of an existing framework of PPI contributions to guidelines, themes and differences between groups were assessed using a revised version of the PCORI conceptual model of patient-centered outcomes research [21]. Prior to finalization of the current analysis, existing studies of PPI in guidelines [16, 22–33] were reviewed by the principal investigator (MJA) and results categorized within the PCORI model. The draft model was reviewed by co-investigators to achieve consensus on placement of themes within the model (Table 1, Additional file 1). Results from the current study were then framed within that model. The PCORI conceptual model, literature review, and current results were subsequently combined into a model relating to guidelines rather than research.

## Results

Nineteen individuals consented to participate: two methodologists, two facilitators, two dementia content experts, two dementia imaging content experts, seven members of the guideline subcommittee with and without dementia expertise, and four lay participants (one person with mild cognitive impairment [MCI], the spouse of the person with MCI, the spouse of a person with dementia, and an advocate from the Alzheimer’s Association) (Table 2). Two guideline subcommittee members contacted for participation declined (one had not volunteered and one was too busy), and five content experts contacted for participation declined (due to busyness, schedule conflicts, and/or conflicts of interest). No volunteer was turned down for participation. One consenting content expert was unexpectedly unable to travel as planned; guideline subcommittee members with dementia expertise were used to maintain balance in content expertise between the two groups. Eight panel members were randomized to each group, with each group supported by a methodologist and a staff person.

**Table 2:** Participant demographics

Characteristic	Experimental group (*n* = 9)	Control group (*n* = 9)
Gender (male)	5 (55%)	5 (55%)
Race		
White	9 (100%)	7 (78%)
Other	0 (0%)	2 (22%)
Age		
30–40 years old	0 (0%)	2 (22%)
40–50 years old	4 (44%)	2 (22%)
50–60 years old	2 (22%)	4 (44%)
60–70 years old	0 (0%)	1 (11%)
> 70 years old	3 (33%)	0 (0%)

### Meeting results

Question development was finished by both groups at the retreat. After the combined training, the experimental group met for longer than the control group (4 h 11 min versus 2 h 55 min; difference 1 h 16 min), but the control group forgot to draft plain-language question versions. The experimental group drafted eight over-arching PICOT questions versus four drafted by the control group, but the control group nested multiple populations within each PICOT question. In the control group, the methodologist, facilitator, content expert, and one of the guideline content experts accounted for 82% of the transcript word count. In the experimental group, the two content experts, facilitator, and guideline content expert accounted for 81% of the transcript word count. The patient, caregivers, and advocate in the experimental group accounted for the same percent of the transcript word count as the non-expert guideline committee members in the control group (14%).

### PICOT questions

The PICOT questions proposed by each group are listed in Additional file 4. The main difference between the two groups was in the proposed outcomes.

#### Population

Both groups identified three populations for guideline questions: individuals at risk for AD dementia without cognitive impairment (preclinical), people with MCI, and people with dementia. The experimental group specified that the MCI population of interest was people with MCI suspected to be prodromal AD. The control group suggested a screening question investigating the prevalence of positive amyloid PET in a general population without AD dementia.

#### Intervention

The intervention for the guideline was, by definition, amyloid PET. Neither group specified individual PET tracers in their questions. The control group asked one question about the diagnostic accuracy of amyloid PET plus a “standard evaluation” in addition to the diagnostic accuracy of amyloid PET alone. The experimental group noted that amyloid PET performed in addition to an exam might be better than either amyloid PET or an exam alone, but did not write a question on this topic.

#### Comparators

Both groups included two overarching comparators: (1) not getting amyloid PET and (2) some kind of other testing. For “other testing,” both groups described clinical evaluation, lumbar puncture with cerebrospinal fluid testing, volumetric magnetic resonance imaging (MRI), fluorodeoxyglucose-positron emission tomography (FDG-PET), and pathology in the setting of a clinical presentation consistent with AD dementia. The experimental group also mentioned tau imaging, functional MRI, ^123^I-Ioflupane SPECT scans, and genomic testing as potential tools that could contribute to reference standards.

#### Outcomes and time

Both groups included accurate diagnosis of AD dementia as an important outcome. For people without cognitive impairment (preclinical population) or with MCI, the experimental group was interested in the development of cognitive impairment due to AD pathology at 1, 3, 5, 10, and 15 years post-scan, whereas the control group was interested in the accurate future diagnosis of AD dementia. The experimental group also included the outcomes of accurate diagnosis of type of dementia and accurate prediction of the rate of decline/progression due to AD dementia.

The control group included a screening question for the general population without AD dementia; the outcome for that question was the prevalence of positive amyloid scans. The control group also asked one question about the outcomes of having an amyloid PET, such as costs, quality of life, benefits/harms of a “correct” diagnosis, stopping the search for other causes, treatment, and preparation for personal and social consequences of AD dementia if diagnosed. The benefits and harms of an AD dementia diagnosis and amyloid imaging were discussed by the experimental group, but the group opted to not include these as specific PICOT outcomes.

### Benefits and harms

Because PICOT questions were expected to focus on diagnostic and prognostic accuracy outcomes, anticipated benefits and harms of amyloid PET use were collected separately to inform recommendation development [1, 2]. The groups described largely overlapping benefits and harms. The control group broadly grouped benefits of amyloid testing as (1) knowing the correct diagnosis, (2) stopping the search for other causes, (3) treatment, and (4) preparing for the personal and social consequences of AD dementia. Specific examples in both groups fell within these categories, including reducing uncertainty, accessing care and support, understanding prognosis to enable long-term planning, and opportunities for research enrollment. Harms described by both groups included misdiagnosis or misprognosis, coping with a correct diagnosis (e.g., depression), discrimination, and loss of job, volunteer, or insurance options.

### Qualitative analysis

After the inductive qualitative descriptive analysis (Additional file 3), differences in group conduct were framed within the proposed conceptual model (Table 1, with numbers below referencing theme numbers within the table).

#### Culture of patient-centeredness

##### Shaping how discussions are conducted (1.1)

Introductions were markedly different between groups. In the control group, participants provided their names, specialties, and academic affiliations. In the experimental group, introductions started with the patient and caregivers who described their personal experiences with cognitive impairment. The advocate gave her name and affiliation, similar to control group physicians. Physician participants in the experimental group described their purpose and role in the project in addition to introducing themselves. Context experts in the experimental group described their backgrounds in depth.

In the control group, guideline subcommittee members without content expertise asked questions about amyloid PET scanning (how it is performed, tracer differences, etc.). These technical details were not discussed in the experimental group. The control group also spent more time discussing comparators/reference standards. In contrast, the experimental group spent more time discussing reasons it is important for patients and families to have a diagnosis in the setting of cognitive impairment. The patient representatives emphasized the importance of having a diagnosis in general, getting closure, validation that something is wrong, reduction in uncertainty, avoidance of unnecessary testing, understanding prognosis, linking to services and support, being able to plan one’s life, enabling formal advance planning, accessing disability and employment protections, accessing targeted treatment and care, and gaining increased control.

##### Setting patient-centered scope (1.2)

Patient representatives emphasized that the amyloid PET topic was important because of its real-life impact:

You know you can talk about the pathology but I have a life to live and the more information I can get about what are my likely paths for my life, that allows me more control over my life. And so, how can you translate the imaging and the other techniques to help me plan my future life? It’s kind of like a business, you know, you want to know the economic climate you’re in, what are your resources, what’s your prognosis and so I’ve got to plan my life based on what you can tell me. (Patient)

##### Describing personal impact of disease (1.3)

The patient and two caregivers described their personal experiences with cognitive impairment in the introduction and with examples throughout the meeting. These testimonies emphasized the guideline’s relevance:

I am caregiving for my husband of 52 years. He was diagnosed with Alzheimer’s in 2009, early stage Alzheimer’s. Subsequently he’s been diagnosed with MCI, subsequently with vascular dementia. Subsequently he has had a spinal tap, the [FDG]-PET scan... MRI, a blood test... Most recently the doctor said “I’m rather certain it’s not Alzheimer’s and I don’t know why you’re here.” And I fell apart because I’ve been caregiving for a very long period of time and my life is devoted to caring for my husband and my husband, according to him he has nothing wrong and is doing just fine and he continues to live as normal a life as possible… It’s been a very long process and actually in conversation with the doctor, he’s going to have the… [amyloid PET] imaging next week. We’ve been actually waiting for a bit to have it. So I will be very relieved to have some closure on what is happening with his brain because on a day to day basis, life is very challenging. (Caregiver-2)

##### Impacting how professional team members view PPI (1.4)

Participants in the experimental group noted the value of the patient voice:

It is really helpful having you here to hear…the decreased uncertainty, increased confidence in diagnosis and prognosis is so important. You know as doctors… we don’t want to do a test if it’s not going to change management, if there’s not a specific therapy that we’re going to offer based on that test… (Moderator)

#### Guidelines relevant to patients/stakeholders (including questions and outcomes)

##### Identifying issues that may be overlooked by medical professionals (3.1)

Patient representatives emphasized the importance of having a diagnosis, challenges of physicians not recognizing that something is wrong, poor communication between primary care physicians (PCPs) and neurologists, and physician reluctance to give a dementia diagnosis:

I am hoping that one of the outcomes of the amyloid testing will be that the diagnosis of the disease will be much more respected with doctors… Doctors don’t want to burden the family with the news when there’s no cure and they don’t really know what to do about it and why should I worry the patient. But I’ll tell you… having gone to 7 doctors before [my husband] was kind of diagnosed, it’s better to know than not to know than not to know. (Caregiver-2)

A content expert in the control group also noted a tendency for PCPs to underdiagnose dementia. The value of early diagnosis was also emphasized by the patient:

I think my life is extremely improved by having that early diagnosis… How can we get to that earlier diagnosis or just channeling say this is something to check up? (Patient)

Both groups mentioned caregiver issues, but this was more common in the experimental group (Additional file 3).

##### Helping select patient-relevant topics and outcomes (3.2)

While the control group was interested in the accurate future diagnosis of AD dementia, the experimental group specified an interest in the development of cognitive impairment due to AD pathology at 1, 3, 5, 10, and 15 years post-scan, as noted above. This emphasis on time-based prognostication was also present in transcripts:

The question really is, Ok can you answer questions that will help me live a better life or you know help me reduce the impact of the disease? So I mean part of it is just tell me I…you know, I have six months to live or six years. And the other one is, you know, what is the progression going to be and is there anything I can do to intervene in that? But it…and I just sense I’m going to repeat myself. I’m going to demonstrate I have this disease, but I mean how do I, you know, both treat it and live with it? (Patient)

Inclusion of time parameters was also based on input from physician panel members. Within the experimental group, there were different views on whether the specific type of future dementia was important. The benefits of a specific diagnosis were emphasized, but for prognostication, the patient felt that the dementia type was less critical:

I mean as a patient I just want to know am I going to get dementia, memory loss… The type depends on how you treat it, but the response to me is the same, I’m losing my mental ability. (Patient, with a mis-speak; treatment depends on dementia type)

##### Influencing guideline structure/development (3.3)

While this study focused on question development, groups identified issues relevant to later recommendation development. Both groups discussed potential harms resulting from PCPs/non-specialists ordering amyloid PET, restricting amyloid PET orders to specialists, establishing minimum standards for ordering, and pre-test counseling (Additional file 3). The experimental group also emphasized that there should be guidance regarding giving a diagnosis of dementia:

Also another very, a really important aspect of it... when a person receives the diagnosis, is how is it presented. The “how” is very, very important because if the neurologist is supportive and can say it in a way that it can be a safe diagnosis, if there’s such a thing for a patient and the caregiver, that makes all the difference… That is going to make a whole, a whole difference in terms of how a patient is going to take this diagnosis. (Caregiver-2)

In the experimental group, patient representatives played an important role in crafting the plain-language versions of the guideline questions, though there was banter regarding whether some suggestions reflected advanced educational backgrounds.

#### Use of results in health decisions/facilitating guideline dissemination and implementation

While the meeting focused on question development, topics pertaining to dissemination and implementation were addressed in both groups. When a guideline committee member in the experimental group described how AAN guidelines typically target general neurologists, the patient and advocate both raised the importance of disseminating the guideline to PCPs:

Can I just say, though, that patients generally interact with their general practice and how do we get this back to that stage, because that’s where I think… You know I was fortunate that my general practice doctor listened to us and you know asked the questions and then got us into looking at this seriously long before I would ever have thought about it and I think my life is extremely improved by having that early diagnosis. (Patient)

If we could get the family doctor to more readily refer to the neurologists; you know if the family doctor is not the one that’s actually doing this, but we need to connect them to neurology; I think sometimes they are in a little bit of a box even if they are in the same building. We’ve had experiences with clinics where the neurology department doesn’t talk to primary care and they are in the same hall. You know so I think… if this starts to establish some communication in that process that will improve the diagnostic process and it will improve outcomes. (Advocate)

Only the control group specifically mentioned creation of a shared decision-making tool. Patient representatives (experimental group) mentioned the importance of recognizing that patients will have differing views on whether they want the test or the results:

Everyone’s coming from a different place with regard to this and the passion that some people will have for wanting this information is …it differs on many different levels. (Caregiver-2)

In some ways from the Alzheimer’s Association’s perspective these [guideline group participants] are some unique individuals. Not everybody takes that position of wanting to know. (Advocate)

As noted above, patient representatives participated in crafting plain-language question versions for the protocol. Only the advocate provided e-mail feedback on the guideline protocol prior to public comment posting.

### Conceptual model

**Fig. 1:**
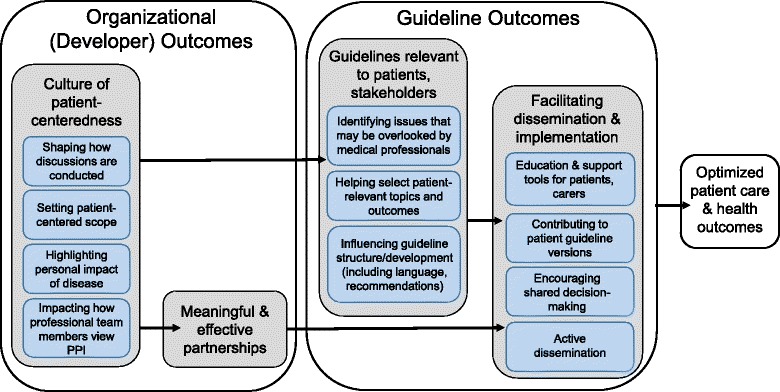
Conceptual model of outcomes of patient involvement in guideline development. PPI patient and public involvement

Based on these study results and the preceding literature review [16, 22–33], the PCORI conceptual model [21] was revised to reflect a conceptual model of outcomes of PPI specific to guidelines (Fig. 1). PCORI’s “near term” and “intermediate” outcomes were reframed as organizational (developer) outcomes and guideline outcomes, with organizational outcomes also influencing guideline development, dissemination, and implementation. The “long-term” PCORI goal of optimal health was reframed as the goal of clinical practice guidelines: optimized patient care and health outcomes [1, 2].

## Discussion

Proposed PICOT questions were largely similar between groups, but the experimental group proposed looking at the future development of cognitive impairment at certain time points (rather than considering development of dementia as a binary outcome) and proposed rate of progression as an outcome. These outcomes relate to the importance of being able to plan for the future—a theme raised by patient representatives—suggesting that this difference relates to the presence of patient representatives and not to other group differences alone. This is also consistent with a systematic review finding that 85% (95% CI 76–94%) of individuals with memory concerns favor disclosure of their diagnosis for reasons relating to autonomy and the ability to plan one’s future [43].

Themes from the experimental and control groups validate the proposed conceptual model of patient and public contributions to guidelines. Patient representatives shaped how retreat discussions were conducted, contributed to a patient-centered scope, described the personal impact of disease, and impacted how physicians in the experimental group viewed the topic and patient involvement, all contributing to a culture of patient-centeredness at the organizational level and within the guideline development process. Patient representatives described issues not raised by participating physicians, identified patient-relevant outcomes, and contributed to discussions of how future recommendations should be framed. Finally, patient representatives participated in crafting of plain-language guideline questions, suggested a broad target audience for the guideline, and identified that patient preferences regarding this topic will vary, all issues with dissemination and implementation implications. Apart from forgetting to develop plain-language guideline questions—something only recently added to the AAN approach—the control group discussion was also patient-centered (e.g., identified benefits and harms largely overlapping with the experimental group, proposal of a decision aid as an important implementation tool), but patient representative participation influenced selected outcomes and group conduct/discussion. These identified PPI contributions to guideline development address barriers to guideline implementation including credibility, stakeholder involvement, and utility/applicability [11–13] and enhance guideline implementability though incorporation of patient needs and preferences [13, 14].

In contrast to prior work describing guideline developer experiences with PPI [16, 23, 24, 28, 31], the results of separate engagement of patient representatives and professionals [22, 27], or feedback from potential or actual guideline group participants [16, 24–26, 33], this study compared the conduct and products of guideline development groups with and without patient representatives, confirming an important role for this population even when physician participants have patient-centered views. This finding is critical, particularly as only 8% of US-based guideline developers require PPI on guideline development groups and only an additional 15% sometimes require it or describe it as optional [44]. A 2008 survey of international guideline developers reported that 39% of responding guideline developers reported PPI on guideline development groups, but only 29% of guideline developers always involved patients or the public and 39% involved them “only if necessary” [45].

Guideline developers describe barriers to PPI in guideline development groups including insufficient resources [29, 46], recruitment difficulties [29], the need for training and support (which is often described as inadequate) [16, 46, 47], uncertainty of how to incorporate patient experiences [33, 46], uncertainty of the patient role [33], patient representatives’ feelings of isolation [29], representativeness of selected participants [29], and patient representatives’ difficulty understanding medical terminology and participating in the systematic review [15, 16, 29, 46, 47]. With increasing research on engagement strategies, however, approaches for successful PPI in guidelines are also identified, including initiation strategies (e.g., diverse recruitment, establishing purpose), building reciprocal relationships (e.g., engaging multiple stakeholders in small panels, equal treatment), co-learning (e.g., training, skilled moderation), practical support (e.g., meeting logistics), and reassessment and feedback [20, 26]. Patient participation in this study followed best practices including involvement of multiple stakeholders with different backgrounds, identifying the purpose of engagement, practical support for meeting logistics, provision of pre-reading and in-person training, equal treatment, and use of skilled moderators. Feedback has not occurred to date as guideline development is ongoing.

The conceptual framework provides a novel model for the impact of PPI on guideline development. It highlights that PPI impacts organizational/developer outcomes as well as individual guidelines. Both outcomes have implications for successful guideline development and implementation. This underscores the need for guideline developers to prioritize PPI in guideline planning (including resource allocation) and further research on optimal methods for overcoming barriers. Research is also needed on the impact of consultation PPI strategies (e.g., reviews of published preferences, surveys, focus groups, public comment) and whether these approaches can meaningfully substitute for active patient participation or only supplement it. Consultation strategies can assist developers in setting patient-centered scope and selecting topics and outcomes but lack the ability to influence outcomes such as shaping discussion conduct, identifying overlooked topics, influencing guideline development, contributing to patient guideline versions, and active dissemination.

### Limitations

This study used a parallel-group design with randomized physician assignment to investigate the impact of engaging patient representatives on guideline development, but results reflect experience from a single guideline, limiting generalizability to other developers/methodologies and topics. The use of retreat group recordings did not permit specific questioning regarding why groups felt certain PICOT elements were of particular importance, but much of this can be gauged from the conversations informing question development. Guideline development groups included only neurologist and patient stakeholders, not other stakeholders (e.g., PCPs, psychiatrists) as is recommended [1, 2, 15]. While content expertise was balanced between groups, observed differences could reflect composition differences other than the presence of patient representatives. Patient representatives accounted for the same proportion of the transcripts as non-experts in the control group, but the “ideal” contribution from patient representatives in guideline development is unknown. Data were acquired solely during question development. This limits conclusions for the impact of PPI at other guideline steps, though retreat discussions touched on these subjects in a manner consistent with the conceptual model. The model outcome of meaningful and effective partnerships could not be fully assessed within the study, though patient representatives were recruited through the local Alzheimer’s Association chapter and this is hoped to promote additional collaborations. It is possible that other researchers would identify additional transcript themes or that researchers’ backgrounds in patient engagement influenced interpretation of results, attributing differences to PPI where others would point to group differences alone. Differences in proposed questions were supported by identified themes, however, and results fit within the referenced model. Finally, research was conducted in partnership with a guideline developer already largely compliant with the IOM standards and with participants aware of the study aims; it is possible that the impact of PPI would be different in other organizations and circumstances.

## Conclusions

This study shows that engaging patient representatives on guideline development groups at the step of question development influences the conduct of guideline development, scope, inclusion of patient-relevant topics, outcome selection, and planned approaches to recommendation development, implementation, and dissemination. The conceptual model showing the impact of PPI on organizations/developers and the guideline development process underscores the importance of engaging patient stakeholders and raises the question of whether guidelines developed without such input are relevant to patients’ needs and implementable. PPI should be considered an essential element of trustworthy guideline development for purposes of development and funding. Further research is needed to identify optimal strategies for PPI involvement in guideline development to address recruitment, panel composition, and mechanisms of engagement.

## Additional files

Additional file 1: Conceptual model of patient and public contributions to guidelines (Complete). Conceptual model table with all examples extracted from literature (not simply sample quotes as provided in table in the text). (DOCX 22 kb)

Additional file 2: COREQ 32-item checklist for manuscript “Impact of Patient Involvement on Clinical Practice Guideline Question Formation: A Randomized Controlled Study”. COREQ checklist. (DOCX 16 kb)

Additional file 3: Qualitative coding from transcripts. Microsoft Word tables showing qualitative coding. (DOCX 183 kb)

Additional file 4: PICOT questions from experimental (Gp1) and control (Gp2) groups. Microsoft Word table of proposed guideline PICOT questions. (DOCX 17 kb)

## Abbreviations

AAN: American Academy of Neurology
AD: Alzheimer’s disease
FDG-PET: Fluorodeoxyglucose-positron emission tomography
IOM: Institute of Medicine
MCI: Mild cognitive impairment
MRI: Magnetic resonance imaging
PCORI: Patient-Centered Outcomes Research Institute
PCP: Primary care physician
PET: Positron emission tomography
PPI: Patient and public involvement
