# Supplementary material for: Impact of patient involvement on clinical practice guideline development: a parallel group study
Source: Implement Sci. 2018 Apr 16;13:55. doi: 10.1186/s13012-018-0745-6 (PMC5902835; doi:10.1186/s13012-018-0745-6)
Supplement: Supplementary file 1 — Conceptual model of patient and public contributions to guidelines (Complete). Conceptual model table with all examples extracted from literature (not simply sample quotes as provided in table in the text). (DOCX 22 kb) [file 13012_2018_745_MOESM1_ESM.docx]

Additional file 1. Conceptual Model of Patient and Public Contributions to Guidelines (Complete)

| **PCORI Conceptual Model – Relevant Outcomes** | **PPI Guideline Contribution** | **Examples from Literature** |
| --- | --- | --- |
| 1. Culture of patient-centeredness | 1.1 Shaping how discussions are conducted | Patients/carers brought “common sense to bear” (Jarrett 2004)  Patients/carers provided a consistent voice on patient-relevant topics (Jarrett 2004)  Patients/carers were a constant reminder of the impact of the guideline (Jarrett 2004)  Patients/carers reminded guideline development group to speak in patient-centered terms (Jarrett 2004)  Patient participation helped keep guideline development patient-focused (van der Ham 2016) |
|  | 1.2 Setting patient-centered scope | Patient representatives elaborated on content and scope of guideline topics, particularly relating to lifestyle and psychological impact of tests, etc. (Tong 2011)  PPI consultations including discussions of social dimensions of illness, beliefs, & behaviors set scope and objectives for guideline (Díaz del Campo 2011)  End users defined scope of systematic review (Coon 2016)  Participants emphasized need for patient involvement in topic selection (Brouwers 2017) |
|  | 1.3 Describing personal impact of disease | Patient/carer presence constant reminder of experience of disease (Jarrett 2004)  Patient involvement helped give “lived experiences” a more central role in guideline development (van der Ham 2016)  Guideline-naïve patients felt they would convey bigger picture of what it’s like to live with the condition (Armstrong 2017)  Contribution of life expertise to accompany technical and educational expertise of physicians (Armstrong 2017) |
|  | 1.4 Impacting how professional team members view PPI | Experience with end-user involvement informed future attitudes (Coon 2016)  Consensus that end-user involvement was worthwhile after initial uncertainty (Coon 2016) |
| 2. Meaningful and effective partnerships | 2. Meaningful and effective partnerships | Individual patient’s participation in guideline development led her organization to discuss how to provide robust input into guideline development (van der Ham 2016)  Patient involvement resulted in developing an implementation plan which included a role for patient organizations (van der Ham 2016)  Patient associations participating in guideline development then incorporated guidelines in educational activities & conferences (Díaz del Campo 2011) (see also implementation/dissemination) |
| 3. Research relevant to patients/ stakeholders (including questions, outcomes) | 3.1 Identifying issues that may be overlooked by medical professionals | **Mentioning patient-relevant symptoms or issues not recognized by professionals**  Mentioning important symptoms not considered by doctors (Jarrett 2004)  Focus groups identified issue not in the literature (lack of anesthesia use when self-harm wounds stitched) (Cowl 2015)  Consultation day with young women with type 1 diabetes identified concern of public weighing in doctors’ offices (Cowl 2015)  Patient input on mental illness guideline emphasized unique topics including vocational limitations, workplace needs, and employment support (van der Ham 2016)  Infertile couples mentioned 8 issues not described by professionals, most relating to patient-centered aspects of care (e.g. taboos, lack of support after treatment) (Den Breejen 2016)  **Clarifying themes that professionals and patients may interpret differently**  Two issues identified by infertile couples & professionals were interpreted differently by the 2 groups (Den Breejen 2016)  **Identifying important sub-populations**  Constantly flagging that children’s issues are different than adults (Jarrett 2004)  **Importance of non-pharmacologic approaches**  Reminding that medication isn’t always an appropriate treatment (Jarrett 2004)  Patient/carer involvement prompted inclusion non-traditional therapies (e.g. aromatherapy) in guideline (Jarrett 2004)  **Prompting holistic approach**  Prompting a holistic approach including psychological and bereavement support (Jarrett 2004)  **Emphasizing importance of role of relatives**  Importance of role of relatives (Jarrett 2004) |
|  | 3.2 Helping select patient-relevant topics and outcomes | **Identifying patient-relevant topics**  Patient/carer involvement strengthened guideline around issue of communication (Jarrett 2004)  Patients/consumers endorsed topic and outcome selection (Tong 2011)  **Selecting and editing guideline questions**  Patient representatives help “feed in” patient and carer issues when developing clinical questions (Graham 2006)  PPI resulted in additional key questions (Graham 2006)  Patients helped write or edit guideline questions (Légaré 2011)  PPI resulted in additional guideline subtopic regarding spectrum and progression of disease (Tong 2011)  Patients helped define key questions, particularly relating to side effects (Díaz del Campo 2011)  End users identified therapies to include in systematic review (Coon 2016)  **Identifying patient-important outcomes**  Patient/carer involvement prompted selection of patient-relevant outcomes (e.g. satisfaction) (Jarrett 2004)  *Patients can help specify all patient-important outcomes and classify the relative importance of those outcomes (Guyatt 2011)*  End users helped put outcomes in a typology (Coon 2016) |
|  | 3.3 Influencing guideline structure/ development | **Impacting guideline structure/approach**  Patients/carers influenced structure of guideline (Jarrett 2004)  Patient/carer involvement prompted inclusion of information specifically for carers (Jarrett 2004)  Patient/carer involvement prompted guideline’s economic review to include funding for self-help, support groups (Jarrett 2004)  Patient/carer engagement prompted guideline section on users’ perspective of treatment (Jarrett 2004)  Patient/carer involvement resulted in guideline chapter on creating services that are acceptable to service users (Jarrett 2004)  PPI resulted in additional guideline chapters on patient issues and on social and psychosocial issues (Graham 2006)  **Participating in systematic review**  Patients involved in synthesizing knowledge, forming recommendations, revising drafts (Légaré 2011)  Patient representatives helped incorporate evidence from grey literature (van der Ham 2016)  End users provided feedback on conceptual model, interim findings (Coon 2016)  End users helped validate and fine-tune  systematic review conclusions, recommendations for future research (Coon 2016)  Patient representatives informed discussion of qualitative research results and helped develop additional considerations relating to conclusions (van der Ham 2016)  Participants described important role in creating and revising draft guideline (and also in creating the final document, while indicating that review only at final draft stage was too late) (Brouwers 2017)  **Influencing recommendation development**  Patients/carers influenced recommendations on issues ranging from responsibility for care to specific therapeutic recommendations; chair felt it was important to test recommendations against patients (Jarrett 2004)  Patients/carers influenced the recommendations about what the National Health Service should provide (Jarrett 2004)  Patient representatives ensure patient/carer views are incorporated into recommendations (Graham 2006)  PPI resulted in guideline recommendations and clinical care suggestions augmented with consumer- focused issues (Tong 2011)  Patient preferences provided context for recommendations (Díaz del Campo 2011)  External review resulted in addition of actions families can take for panic attacks & discussion of need for assessment of family support prior to suggesting lifestyle changes (Díaz del Campo 2011)  PPI resulted in development of consensus recommendations using formal techniques for important issues with no evidence (Cowl 2015)  PPI resulted in recommendations relevant to patient-centered issues (e.g. performing weights in a private room in doctors’ offices) (Cowl 2015)  *PPI in recommendation development can identify whether the problem is a priority, inform meaningful effects, weigh risks & benefits, and assess impact of costs, acceptability, and feasibility (Alonso-Coello 2016, EtD 2)*  **Influencing language used in guideline**  Patient/carer involvement made guideline language more patient-friendly (Jarrett 2004)  Patient/carer involvement impacted guideline wording (Jarrett 2004)  Patients helped make sure guideline used widely understood terminology (Légaré 2011) |
| 4. Use of results in health decisions | 4. Facilitating guideline dissemination and implementation | **4.1 Prompting inclusion of education and support for patients and carers**  Patient/carer involvement prompted inclusion of information and advocacy and support for patients and carers (Jarrett 2004)  Patients and carers noted importance of patient education for self-management (Jarrett 2004)  Patient discussions informed implementation planning including developing interventions to address workplace stigma (van der Ham 2016)  **4.2 Contributing to patient guideline versions**  Patient representatives help draft guideline discussions for patients, carers (Graham 2006)  Patient representatives help select key recommendations for patient guideline versions (Graham 2006)  PPI prompted development of a plain-language guideline version (Tong 2011)  Patients informed appropriate vocabulary and relevant content for patient guideline versions (Díaz del Campo 2011)  Participants described an important role in creating a patient version of the guideline (Brouwers 2017)  **4.3 Encouraging shared decision making**  Patients encouraged patients & health care professionals to partner to make decisions (Légaré 2011)  **4.4 Guiding regarding end-user uptake**  Patients/carers provided guidance on how local services should be involved (Jarrett 2004)  **Actively disseminating**  Patient associations participating in guideline development then incorporated guidelines in educational activities & conferences (Díaz del Campo 2011)  End users helped plan charity event with discussion of findings (Coon 2016) |

PPI: Patient and public involvement

Italics: Policy or development publication rather than research
