# Supplementary material for: Impact of patient involvement on clinical practice guideline development: a parallel group study
Source: Implement Sci. 2018 Apr 16;13:55. doi: 10.1186/s13012-018-0745-6 (PMC5902835; doi:10.1186/s13012-018-0745-6)
Supplement: Supplementary file 2 — COREQ 32-item checklist for manuscript “Impact of Patient Involvement on Clinical Practice Guideline Question Formation: A Randomized Controlled Study”. COREQ checklist. (DOCX 16 kb) [file 13012_2018_745_MOESM2_ESM.docx]

**Additional file 2.** COREQ 32-item checklist for manuscript “Impact of Patient Involvement on Clinical Practice Guideline Question Formation: A Randomized Controlled Study”

| **No. Item** | **Guide questions/description** | **Reported on Page #** |
| --- | --- | --- |
| **Domain 1: Research team and reﬂexivity** |  |  |
| *Personal Characteristics* |  |  |
| 1. Interviewer/facilitator | Which author/s conducted the interview or focus group? | There were no interviews or focus groups for this study; qualitative analysis was performed on retreat group transcripts. All retreat group participants were study subjects, including the facilitators (Methods, page 6-9).  Research credentials are provided on the title page for the reader. The PI introduced herself to the participants during the training at the retreat, but this COREQ section is not quite applicable given that there were no interviews or focus groups. Participants reviewed an informed consent document prior to participating (Methods/ Recruitment p. 6-7) which described the reasons for the research. The main analyst’s positionality is described on page 8. There is discussion of possible impact of the analyst’s positionality on page 22. |
| 2. Credentials | What were the researcher’s credentials? E.g. PhD, MD |  |
| 3. Occupation | What was their occupation at the time of the study? |  |
| 4. Gender | Was the researcher male or female? |  |
| 5. Experience and training | What experience or training did the researcher have? |  |
| *Relationship with participants* |  |  |
| 6. Relationship established | Was a relationship established prior to study commencement? |  |
| 7. Participant knowledge of the interviewer | What did the participants know about the researcher? e.g. personal goals, reasons for doing the research |  |
| 8. Interviewer characteristics | What characteristics were reported about the inter viewer/facilitator? e.g. Bias, assumptions, reasons and interests in the research topic |  |
| **Domain 2: study design** |  |  |
| *Theoretical framework* |  |  |
| 9. Methodological orientation and Theory | What methodological orientation was stated to underpin the study? e.g. grounded theory, discourse analysis, ethnography, phenomenology, content analysis | Methods/Analysis, p. 8-9 (Conceptual model p. 9) |
| *Participant selection* |  |  |
| 10. Sampling | How were participants selected? e.g. purposive, convenience, consecutive, snowball | Methods/Recruitment, p. 9 |
| 11. Method of approach | How were participants approached? e.g. face-to-face, telephone, mail, email | Methods/Recruitment, p. 6-7 |
| 12. Sample size | How many participants were in the study? | Methods/Recruitment, p. 6-7  Results, p. 9-10 |
| 13. Non-participation | How many people refused to participate or dropped out? Reasons? | Results, p.9-10 |
| *Setting* |  |  |
| 14. Setting of data collection | Where was the data collected? e.g. home, clinic, workplace | Methods/Data collection, p. 7-8 |
| 15. Presence of non-participants | Was anyone else present besides the participants and researchers? | Results, p. 9-10 (Each group supported by a methodologist and a staff person) |
| 16. Description of sample | What are the important characteristics of the sample? e.g. demographic data, date | Results, p. 9-10; Table 2 |
| *Data collection* |  |  |
| 17. Interview guide | Were questions, prompts, guides provided by the authors? Was it pilot tested? | N/A (comparing two discussions) |
| 18. Repeat interviews | Were repeat interviews carried out? If yes, how many? | N/A (comparing two discussions) |
| 19. Audio/visual recording | Did the research use audio or visual recording to collect the data? | Methods/Data collection, p. 6, 8 |
| 20. Field notes | Were ﬁeld notes made during and/or after the inter view or focus group? | N/A |
| 21. Duration | What was the duration of the interviews or focus group? | Results/Meeting results, p.10 |
| 22. Data saturation | Was data saturation discussed? | N/A (comparing two discussions) |
| 23. Transcripts returned | Were transcripts returned to participants for comment and/or correction? | N/A (comparing two discussions) |
| **Domain 3: analysis and ﬁndings** |  |  |
| *Data analysis* |  |  |
| 24. Number of data coders | How many data coders coded the data? | Methods/Analysis, p. 9-10 |
| 25. Description of the coding tree | Did authors provide a description of the coding tree? | Supplemental materials |
| 26. Derivation of themes | Were themes identiﬁed in advance or derived from the data? | Methods/Analysis, p. 9-10 |
| 27. Software | What software, if applicable, was used to manage the data? | Methods/Analysis, p. 9-10 |
| 28. Participant checking | Did participants provide feedback on the ﬁndings? | N/A |
| *Reporting* |  |  |
| 29. Quotations presented | Were participant quotations presented to illustrate the themes/ﬁndings? Was each quotation identiﬁed? e.g. participant number | Results, p.12-18  Additional file 3 |
| 30. Data and ﬁndings consistent | Was there consistency between the data presented and the ﬁndings? | Results, discussion, p. 12-21 |
| 31. Clarity of major themes | Were major themes clearly presented in the ﬁndings? | Results, p. 12-18, additional file 3 |
| 32. Clarity of minor themes | Is there a description of diverse cases or discussion of minor themes? | Results, p. 12-18, additional file 3 |
