# Supplementary material for: Impact of patient involvement on clinical practice guideline development: a parallel group study
Source: Implement Sci. 2018 Apr 16;13:55. doi: 10.1186/s13012-018-0745-6 (PMC5902835; doi:10.1186/s13012-018-0745-6)
Supplement: Supplementary file 3 — Qualitative coding from transcripts. Microsoft Word tables showing qualitative coding. (DOCX 183 kb) [file 13012_2018_745_MOESM3_ESM.docx]

**Additional file 3. Qualitative Coding from Transcripts**

Table 1. Introductions

Notes: Introductions in the patient group were much more expansive than in the control group (I’m not copying and pasting all the intros to show that, but I have the transcripts).

| Theme | Gp 1 (Exper, WITH PATIENTS) | Gp 2 (Control, PHYSICIANS ONLY) |
| --- | --- | --- |
| Apologizing for memory by patient representative | **Apology during introduction**  ***, um, I think I mentioned before my background to academic administrator, um, I’ve very interested in this. I won’t be able always to recall all the details. (P-P1)  **Apology later in session**  But it…and I just sense I’m going to repeat myself. I’m going to demonstrate I have this disease (P-P1) |  |
| Personal experience and views as part of introduction | ***, um, I think I mentioned before my background to academic administrator, um, I’ve very interested in this. I won’t be able always to recall all the details. My point really is to try and stress the fact the difference that I feel between memory and cognition and *how we can help people understand that that sense that a person with memory loss has lost memory. They have not lost their sense of self and how important that is in interactions*. (P-P1)  ***, I’m a care partner for my husband *** who’s diagnosed with MCI in 2012 and so we’ve been on this journey for a few years and really happy to contribute if we can. (P-CP1)  I’m ***. I am caregiving for my husband of 52 yrs. He was diagnosed with Alzheimer’s in 2009, um, early stage Alzheimer’s. Subsequently he’s been diagnosed with MCI, subsequently with vascular dementia. Subsequently he has had a spinal tap, the PET scan, the PAT F18, um, MRI, a blood test. He’s APOE3 rather than 4. I have his file here. Um, and most recently the doctor said I’m rather certain its not Alzheimer’s and I don’t know why you’re here. And I fell apart because I’ve been caregiving for a very long period of time and my life is devoted to caring for my husband and my husband, according to him has nothing wrong and is doing just fine and he continues to live as normal a life as possible. And I am his constant companion, my daughter has him today. Um, but it’s been a very long process and actually in conversation with the doctor, he’s going to have the imaging, the (inaudible) imaging next week. We’ve been actually waiting for a bit to have it. So I will be very relieved to have some closure on what is happening with his brain because on a day to day basis, um, life is very challenging. On the moca he has consistently scored 29-30 for 5 years. Happy to be here and be a part of this very important endeavor. (P-CP2)  [Later comment regarding background]  I am the one who is involved and just by the by, I’m also on that um, beneficiary family advisory counsel for Medicare and um, it’s just…it’s a new patient committee that started um in November, um, and they are listening, they are actually listening to…I’m the only one representing Alzheimer’s so I’m already hearing some changes in that. Every meeting we have once a month and now we’re having workgroups, I am um, relentless. (P-CP2)  **Advocate gives more professional (physician-like) introduction**  ***, VP of Education for Alzheimer’s association of Minnesota/North Dakota. | [All one-line intros, specialty/hospital] |
| Describing self as non-expert | I’m definitely not a content expert as most of you noted in this field in any way shape or form (P-Moderator)  I’m not an expert in any way of dementia (P-Method) |  |
| Establishing professional expertise | Just to give you a little bit of my background, I was born and raised in *** and went to Medical School at ***and then dd my behavior neurology residency at ***, my fellowship at *** with internationally renowned behavioral neurologist ***. I was on faculty there for a decade, held a number of education and leadership positions there including running their behavioral neurology division. I’m a card carrying behavioral neurologist and thus one of your content experts for today. I recently moved, literally a year ago to ***. I am the director for aging brain and behavioral neurology for *** which is a large hybrid system, in some ways similar to what you might hear about like with Kaiser that has both a health insurance and a health care system part, it has both in patient and out patient facilities, covers a large patient population. Pennsylvania has the #2 eldest population in the United States. I came from #1, Florida, but unlike Florida Pennsylvania has to import, I’m sorry, Florida has to import their elders, Pennsylvania makes them. And basically we are looking to bend the spectrum on aging brain (inaudible) from prevention and health brain aging to early detection of people at risk for further decline and then precise diagnosis so people know what they are dealing with as early as possible so that we can get the best interventions in as well as have a clear idea of what we’re trying to help with when we do research. And that’s…and help people throughout the care spectrum including later stages, end of life care and planning and all those things. (P-Ex1)  I’m *** I’m a cognitive neurologist at ***. I direct our memory center and I run the clinical core of our Alzheimer’s Disease center and I guess I’m the imaging content expert, um, and I do a fair bit of work in both MR and PET imaging and actually I think authored the first or one of the first MCI papers with (inaudible) initial imaging agent. (P-Ex2) | *** (inaudible) a Behavioral Neurologist at * (C-Ex1) |
| Establishing purpose and role | My job will be just to make sure that we progress through the whole process and end up with deliverables at the end… (P-Mod)  My role in this group is as a methodologist, so in case of our rules questions or how do we do this or what the format should some think be in, um, that’s basically my job and I’ll be writing on the board so we can record stuff. (P-Method)  I’m the guideline development program manager here at the AAN and so I will be working with all of you, the methodologists and the guidelines committee on the development of the guideline. (P-Staff) | [All one-line intros] |

Table 2. Themes and Example Quotes Relating to Clinical Care and Patient-Centered Themes

| Theme | Gp 1 (Exper, WITH PATIENTS) | Gp 2 (Control, PHYSICIANS ONLY) |
| --- | --- | --- |
| Amyloid-PET is not widely available | I really appreciate that when I actually had that on my list of things that I want to talk about as well as these alternatives because I don’t know is the amyloid imaging available everywhere? Is…are these alternatives (P-Mod)  No it isn’t (P-CP2)  So even like regionally or locally may not be available to everybody (P-Mod)  No (P-CP2)  I also wonder if under harm and I don’t know maybe it doesn’t live here, but accessibility is a potential harm. Talking about…because the scenario you just went through makes me think looking at some of the chapters of the association and areas where there is not the ability of a potential patient to access any of this. I mean they’re lucky if they have a primary care physician let alone a neurologist and they man have to travel miles which may or may not be possible, they could be alone, they may not have a primary caregiver. So you know if we make…if we recommend this as a guideline, but PS here’s where you have to live to be able to (P-Adv)  It’s basically um, with the FDA approved tracers anywhere that has a PET scanner would potentially be pretty accessible. I actually think the less accessible thing is actually the memory expert um, in particular areas of the country because even…I mean there are certainly areas of the country where there’s no PET scanner place or several hours away but with the F18 tracers they can be made at manufacturing sites and sent off and so there’s fairly good accessibility but not perfect. (P-Ex2)  But having some sort of standard that you have to have a minimum knowledge and show that you know how to interpret and use these results in a clinical context I think would be important if you’re going to do that for accessibility. (P-Ex1)  **Payor considerations**  I think in the clinic setting when they can you know, this would be a great test, here’s a great tool except you have to pay for it. It’s not an option for us to have Medicare payer an insurance payer however this ends up…however many years down the road this is. (P-Adv)  I mean the problem with that for this particular topic is that if it’s not being paid for it’s not available for the vast majority of the population so we have to be…you know weigh that I think reasonably. (P-Mod) | So only certain hospitals may be able to do this test? (C-GDDI2)  Correct (C-Ex1)  At this point in time I think there’s…and it’s not especially complicated so (inaudible) spect (inaudible) technology (C-GDDI-Ex1)  Anyone who can do an FDG PET for the body should be able to eventually do an amyloid PET. (C-Ex1)  And by any…from a clinical perspective once you have you know some basic knowledge of what’s normal versus what’s abnormal you can get …with a short lead in time you can become pretty familiar with what you’d expect in the right patient population. (C-GDDI-Ex1)  The grading is pretty…it’s not an issue, especially they are a highly reliably graded in terms of what’s positive, what’s negative. I think that’s… (C-Ex1)  (talk over) right and we haven’t been using the scanning technology because it’s otherwise, previously it was $4600 out of pocket. (C-GDDI-Ex1) |
| Challenges to clinical care | **Memory experts are not widely available**  I actually think the less accessible thing is actually the memory expert um, in particular areas of the country because even…I mean there are certainly areas of the country where there’s no PET scanner place or several hours away but with the F18 tracers they can be made at manufacturing sites and sent off and so there’s fairly good accessibility but not perfect. But the memory expert’s more of an issue and actually the Society for Nuclear Medicine/Alzheimer’s Association appropriate use criteria, this was one of the major sort of um, uh areas of conflict was who can order these studies and how do we define, if we do say it has to be a memory expert, how do you define memory expert, what portion of patients do you need to see, what kind of training do you need to have and part of that was driven, uh I think by things that P-Ex1 was saying in terms of it being ordered in the appropriate context… (P-Ex2)  **Diagnosis of Alzheimer’s disease not “respected” by physicians [similar to under diagnosis in Gp 2)**  I am hoping that one of the outcomes of the amyloid testing will be that the diagnosis of the disease will be much more respected with doctors. Because as it stands now we hear different… differences in numbers where 33% of those really who have Alzheimer’s or dementias are actually diagnosed, 46%, but it’s all below 50% and that’s what we keep hearing over and over again and the rationale…P-Adv could speak to this completely, is that doctors don’t want to burden the family with the news when there’s no cure and they don’t really know what to do about it and why should I worry the patient. So…but I’ll tell you having spent…having gone to 7 doctors before [my husband] was kind of diagnosed, it’s better to know than not to know than not to know. (P-CP2)  **Doctors not recognizing something is wrong**  I am caregiving for my husband of 52 yrs. He was diagnosed with Alzheimer’s in 2009, um, early stage Alzheimer’s. Subsequently he’s been diagnosed with MCI, subsequently with vascular dementia. Subsequently he has had a spinal tap, the PET scan, the PAT F18, um, MRI, a blood test. He’s APOE3 rather than 4. I have his file here. Um, and most recently the doctor said I’m rather certain its not Alzheimer’s and I don’t know why you’re here. And I fell apart because I’ve been caregiving for a very long period of time and my life is devoted to caring for my husband and my husband, according to him has nothing wrong and is doing just fine and he continues to live as normal a life as possible. And I am his constant companion, my daughter has him today. (P-CP2, part of her introduction)  **Poor communication between FPs and neurologists**  Well because even if we could get the family doctor to more readily refer to the neurologists, you know if the family doctor is not the one that’s actually doing this but we need to connect them to neurology I think sometimes they are in a little bit of a box even if they are in the same building we’ve had experiences with clinics where the neurology department doesn’t talk to primary care and they are in the same hall. You know so I think even if this starts to establish some communication in that process that will improve the diagnostic process and it will improve outcome. (P-Adv) | **Memory experts are not widely available**  The trouble is, what will affect that recommendation is the feasibility, the availability; you can’t get in to see a lot of the dementia specialists because they’re so busy (talk over) (C-Method)  Shortage and geographical amount distribution.(C-GDDI4)  I think one important thing is looking at if we’re going to say dementia specialist as a category I don’t even know what percent of dementia patients you said gets seen by dementia specialists. We may be talking about an extremely small percentage of people in the world. (C-Ex1)  **Under diagnosis by PCPs**  Or not diagnose. You’re saying once they diagnose them they over treat but the vast majority I’d say under diagnose than (C-Ex1)  **Risk of over treatment when PCPs diagnose MCI or dementia**  So one of the issues that I think is out there but I don’t know if you guys can enlighten us, is when a primary care physician or non neurologist diagnose um, dementia or Alzheimer’s disease often time’s it’s sort of this blanket dementia. Those patients tend to be under treated with um medications, you know you’re run of the mill (inaudible) medications. Um so if they order the test and they got a positive test at what…first of all do you think that’s true or not true. Second thing is if they ordered the amyloid PET and they had a positive, would that you know…I suspect it may make them more likely, here I think it’s because they’re not very familiar with clinical criteria so they are not very sure. Would that make them more likely to treat them and if so is that a good thing or a bad thing? (C-Mod)  I mean I see that when a primary care makes a diagnosis of dementia they’re more likely to treat. (C-GDDI-Ex2)  And over treat. They come in on memantine and cholinesterase inhibitors even though… In the MCI phase… Even though the evidence is questionable (C-GDDI-Ex1)  So probably they should get them a gym membership rather than memantine (C-GDDI4)  Yeah exactly I mean the insurance would be better and (talking over) (C-GDDI-Ex1)  And everything like that but primary care tends to over treat (C-GDDI-Ex2) |
| Ordering test should be limited to specialist | **Who should order test**  So where does that amyloid testing…does that fall into just the neurologist, does it fall into, you know if it’s really effective does it fall into for an internist general practitioner, are they going to have access to that (P-CP1)  **Potential harm from ordering by PCPs/non-specialists**  …in terms of it being ordered in the appropriate context and part of that I think was a bit of an eye towards coverage with Medicare that if we open this up to everyone there would be the primaries who don’t feel comfortable making a diagnosis and routinely send for neuro-psychological testing it’s like we don’t need to do that anymore, we can just order this scan and so, you know harm I think of…of amyloid imaging we have to think about is just a resource harm, societal resource, you know what is the cost of scanning in terms of what recommendations we make, um how…you know what are the sort of economics of it as well which I think is again another part of the question to answer. (P-Ex2)  I think the big harm I was concerned about and most of my colleagues in terms of it being ordered just on…by non-cognitive experts whatever you want to describe that is the interpretation of it because um, basically depending on … we know from um, some very good studies that while amyloid in the brain probably puts you in a high risk category for having Alzheimer’s, it is not destiny. (P-Ex1)  I think this is important but I think that’s also why I’m still kind of pushing for broad rather than specific and also pushing for it being in the had of memory specialists because what I’m going to tell you is the primary care doctor is not going to pick up on those early enough often times and I’m going to tell you an amyloid imaging study is not going to tell you that. (P-Ex1)  But what I’m going to point out is you’ve heard how detailed P-Ex2 and I’s discussion has been about the use of amyloid imaging and what it means, when it means something, when it doesn’t mean something. So my horror is the idea that you get a bunch of people doing these imaging studies and Ok…I mean we spend our lives just looking at this stuff and talking about this stuff and you’ve seen our level of back and forth, now imaging having that done by people in primary care. You’re going to get people who are diagnosed with Alzheimer’s who don’t have any problem, you’re going to have people with other dementias who are told you’re fine, you’re going to have…and I would have a strong fear of having this kind of study in the hands of the general practitioner…just because they, I mean we work hard to put it into context. I think this kind of study has to be a gate kept by the cognitive resource specialist not because Oh I’m afraid we’re going to run Medicare into the ground with costs, but because I’m afraid of the impact it’s going to have on people, not getting diagnosed properly. (P-Ex1)  I absolutely agree and I wasn’t indicating that it should be by a primary doctor by any means… (P-CP2)  I mean if there were a scanner on every corner we’d be in real trouble. (P-Ex2)  **Only specialist should order test**  None of these [tests] can [diagnose dementia] accurately enough to be used in just a primary care setting or in isolation. All of them need to be put into the context of the bigger picture and really starts with this neurologic and cognitive thing. If you don’t have that to put it into context you’re going to be lost. So what I’m going to tell you is no matter what we pick or suggest to highlight I think all of these markers and amyloid included have to be interpreted and put into context by a memory disorder specialist. It cannot be done at the primary care level. (P-Ex1)  I think the big harm I was concerned about and most of my colleagues in terms of it being ordered just on…by non-cognitive experts whatever you want to describe that is the interpretation of it (P-Ex1)  I think that’s also why I’m still kind of pushing for broad rather than specific and also pushing for it being in the had of memory specialists because what I’m going to tell you is the primary care doctor is not going to pick up on those [safety issues] early enough often times and I’m going to tell you an amyloid imaging study is not going to tell you that. Um, so those are very important. (P-Ex1)  But what I’m going to point out is you’ve heard how detailed P-Ex2 and I’s discussion has been about the use of amyloid imaging and what it means, when it means something, when it doesn’t mean something. So my horror is the idea that you get a bunch of people doing these imaging studies and Ok…I mean we spend our lives just looking at this stuff and talking about this stuff and you’ve seen our level of back and forth, now imaging having that done by people in primary care. You’re going to get people who are diagnosed with Alzheimer’s who don’t have any problem, you’re going to have people with other dementias who are told you’re fine, you’re going to have…and I would have a strong fear of having this kind of study in the hands of the general practitioner…just because they, I mean we work hard to put it into context. I think this kind of study has to be a gate kept by the cognitive resource specialist not because Oh I’m afraid we’re going to run Medicare into the ground with costs, but because I’m afraid of the impact it’s going to have on people, not getting diagnosed properly. (P-Ex1)  I absolutely agree and I wasn’t indicating that it should be by a primary doctor by any means,.. (P-CP2)  I absolutely concur, but let me say that does impact accessibility potentially if we really limit it to uh Alzheimer’s disease centers for example, but I mean do you agree completely or do you think there could be some…it could be ordered by a non-cognitive behavioral neurologist? (P-Mod)  So I think it’s… cognitive behavioral neurologist… But also there are, you know, memory experts, psychiatrists and geriatricians and others who have expertise in this area (P-Ex2)  Or somebody like me, so I’m a general neurologist trained through residency most of my practice is epilepsy, I see a few (inaudible) patients in the hospital so I have a hospital practice. Where do I fit. Is there, shouldn’t there be a specify…we’re going to think about general practitioners versus sub specialists with special training and/or knowledge, should there be a middle part or not. (P-Method)  Yeah so I mean that’s a question, um, which I think is an interesting one. My gut is that based on my interactions even with my colleagues at a tertiary care center is that sort of the understanding of what it means to have a positive scan and in what context is limited even within sort of…even very bright people like P-Moderator (laughing over talking) (P-Ex2)  What I was going to say, I know for sure that I should not be allowed to order it (P-Mod)  *Have to be careful that it is not over-ordered*  If we open this up to everyone there would be the primaries who don’t feel comfortable making a diagnosis and routinely send for neuro-psychological testing it’s like we don’t need to do that anymore, we can just order this scan (P-Ex2)  iIf there were a scanner on every corner we’d be in real trouble. (P-CP2)  *Possibility of using a certificate program to gain expertise for ordering*  You know I mean…Ok, here would be the way to sort of split that maybe to increase accessibility without necessarily…the way would be to develop some sort of certification for ordering and using amyloid imaging. People like David and I will pass it with our eyes closed. Neurologists in general practice who want to offer it to their patients and maybe there is…if you’re out there in the middle of nowhere and you’re like the only…I’ve got buddies in Montana and they’re the only neurologist for several hundreds, I don’t know how far out, miles, and getting to a memory specialist ain’t going to happen. They may want to step up and take you know…you know a little extra studying, take that test so they know what to do with it at least at a…you know at the level they can, I think that’s a reasonable…and it’s wouldn’t just be neurologist, it could be psychiatrists who have an interest, geriatric, the rare generalist might do this especially if they are the only generalist out in you know Alaska. They may still go for it. But having some sort of standard that you have to have a minimum knowledge and show that you know how to interpret and use these results in a clinical context I think would be important if you’re going to do that for accessibility. (P-Ex1)  **Who is a specialist**  I absolutely concur, but let me say that does impact accessibility potentially if we really limit it to uh Alzheimer’s disease centers for example, but I mean do you agree completely or do you think there could be some…it could be ordered by a non-cognitive behavioral neurologist? (C-Mod)  So I think it’s…I mean…it’s cognitive behavioral neurologist… But also there are, you know memory experts, psychiatrists and geriatricians and others who have expertise in this area. (P-Ex2)  Or somebody like me, so I’m a general neurologist trained through residency most of my practice is epilepsy, I see a few (inaudible) patients in the hospital so I have a hospital practice. Where do I fit. Is there, shouldn’t there be a specify…we’re going to think about general practitioners versus sub specialists with special training and/or knowledge, should there be a middle part or not. (P-Method)  Yeah so I mean that’s a question, um, which I think is an interesting one. My gut is that based on my interactions even with my colleagues at a tertiary care center is that sort of the understanding of what it means to have a positive scan and in what context is limited even within sort of…even very bright people like P-Mod (laughing over talking) (P-Ex2)  What I was going to say, I know for sure that I should not be allowed to order it. (P-Mod)  **Minimum standards for ordering**  You know I mean…Ok, here would be the way to sort of split that maybe to increase accessibility without necessarily…the way would be to develop some sort of certification for ordering and using amyloid imaging. People like David and I will pass it with our eyes closed. Neurologists in general practice who want to offer it to their patients and maybe there is…if you’re out there in the middle of nowhere and you’re like the only…I’ve got buddies in Montana and they’re the only neurologist for several hundreds, I don’t know how far out, miles, and getting to a memory specialist ain’t going to happen. They may want to step up and take you know…you know a little extra studying, take that test so they know what to do with it at least at a…you know at the level they can, I think that’s a reasonable…and it’s wouldn’t just be neurologist, it could be psychiatrists who have an interest, geriatric, the rare generalist might do this especially if they are the only generalist out in you know Alaska. They may still go for it. But having some sort of standard that you have to have a minimum knowledge and show that you know how to interpret and use these results in a clinical context I think would be important if you’re going to do that for accessibility. (P-Ex1) | **Potential harm from ordering by PCPs/non-specialists**  Getting back to the setting you’re going to get vendors that will push the tests and have a much less selective population from a general physician or general healthcare practitioner so potentially the harm could go up in terms of the false positives. (C-GDDI4)  Yeah it’s interesting that you said that the appropriate use criteria said by dementia specialists. Are most patients diagnosed with dementia or Alzheimer’s disease by dementia specialists? I think not. And so…I mean this has pretty big implications if there’s evidence that um, non dementia specialists are much more inaccurate and the test becomes much less useful. (C-Method)  So then I guess one of the issues that comes up within the scenario is uh I don’t want a situation where um, a confused 70 yr old is admitted to my service and before I go in the next morning, the residence have ordered amyloid PET, you know, it’s overuse. (C-Mod)  No the emergency room will do it (C-GDDI4)  You know you’d be surprised at what happens. You know it will be for those patients that they will get it done from my outpatient clinic I’ll be fighting for someone and it won’t get done. But, no. So what I mean I was just being factious… but overuse. (C-Mod)  I think that’s why the…certain people ordering it in the right context (talk over) (C-Ex1)  Well frankly I’m not worried about amyloid PET in your [dementia specialist] hands, I don’t know I think C-GDDI3 made this point or somebody. I’m worried about it just if it’s out there that a lot of harm will be done because (C-Method)  Even the way it’s written and there’s harm being done now like I said I’ve seen these cognitively normal physicians who have gotten an amyloid PET from a friend you know that’s an issue. (C-Ex1)  **Only specialist should order test**  Getting back to the setting you’re going to get vendors that will push the tests and have a much less selective population from a general physician or general healthcare practitioner so potentially the harm could go up in terms of the false positives. (C-GDDI4)  Yeah it’s interesting that you said that the appropriate use criteria said by dementia specialists. Are most patients diagnosed with dementia or Alzheimer’s disease by dementia specialists? I think not. And so…I mean this has pretty big implications if there’s evidence that um, non dementia specialists are much more inaccurate and the test becomes much less useful. (C-Method)  I think one of the things that they were getting at is this idea of you know if someone has Lewy body dementia which could be very hard to diagnose, uh getting that test is not going to inform you um and so there’s certain subtles to it that require that you know if you’re not a dementia expert but certainly a lot of expertise in the area meaning. (C-Ex1)  So would setting basically end up under clinical context since there probably won’t be studies comparing amyloid PET as ordered by a GP versus a dementia specialist? (C-GDDI4)  Well I think we can catch that…I can see catching that in a recommendation in the rationales’ to the recommendations. Um because if we find evidence and I think you will that if you’re a non dementia specialist you’re really inaccurate and their pretest probability is going to drop and their false positive and false negative rates are going to go up and then we’ll be able to catch that you know, we won’t find any direct evidence related to when…dementia experts doing amyloid PET versus non dementia experts these are the outcomes, we won’t find that but I think we can find the direct evidence. (C-Method)  Well there will be that issue but then the other issue is you’re going to know coming in that the diagnostic performance of the test is going to be a lot less in the non-dementia experts and then we’re going to need…and then…and so a potential recommendation is the only person that should order tests are dementia specialists. Nobody else should be ordering tests. I mean that would be reasonable. You know if that’s what you find. You will be the gate keepers to amyloid PET. (C-Method)  Well the alternative is that we rule that there is an incremental benefit in dementia experts and then we extend that to say think of how big the benefit would be in non experts. This test makes us more accurate, think what it would do for people who start with a lower accuracy. It’s presumably the accuracy of the test is fixed in that (talk over) (G-GDDI-Ex1)  But you’ve got to know how to interpret it, that’s key (C-Ex1)  I know, I agree (C-GDDI-Ex1)  Yeah just the pretest probability rule (talk over ) (C-Method)  **Who is a specialist**  That’s interesting, do you define it as someone who is a neurologists, psychiatrist or geriatric specialist who has 25% or more of their time spent for dementia. (C-GDDI-Ex2)  **Minimum standards for ordering**  That would be no different from genetic counseling for neurodegenerative diseases. I mean you need certain training to be able to do that and we all have disaster stories of people that have test done with no counseling. (C-GDDI4) |
| Importance of screening to detect cognitive impairment in the first place  **(A little off topic but highly patient relevant – could be example of how patients get off track or of how they properly anchor guideline in bigger picture…)** | Yeah I guess I’m going to…back to before the person sees the neurologist. That’s where I see the hardest part is when does the person go to the doctor with a memory problem, that’s when P-P1 and I went and talked to our physician about because we were concerned about memory. But there’s…and there’s people who are in denial, nothing’s wrong with me and so…and that’s where one of the things is like, you know, do you do the mini-cog on a regular basis with your regular physician because not everybody just goes to a neurologist to start with. So when does that start and if there was a better answer like with the amyloid that the regular physician could say, Ok, you know we went through the MRI, blood work, just to rule out any pathological and then we went to the neuro psychological testing and then we were referred to a neurologist after that. (P-CP2)  And as part of that I’ve been talking a lot with primary care providers and what they can do and what they can’t do in their clinical practice setting… So basically what we’ve come up with is the idea that for primary care, really for at risk patients and let’s you know we have arbitrarily picked 65 and older for screening… so those people we are arranging to eventually have them do yearly screenings, you know brief cognitive screens because you’re right. One of the big parts of these…two things with these diseases, !) many of them are insidious, they sneak up on you and 2) a lot of them do have what you are talking about, denial… Um, I think primary care is vitally important for detection, Ok. Detecting who is at risk. But using things like mini cog and stuff, you can use those things and say Ok you’re at risk now, but that doesn’t mean you have a diagnosis, that does not even mean you have a problem it means that we detected that there’s something wrong and that’s the idea of getting early. And then we’re working through a process because there’s a lot of things that can throw off your brain. The brain is very complex, it needs to be in the happy place physically so we have them screening for depressing, we have them screening for sleep apnea, we have them screening for the multiple central nervous system impairing medications that many of our elders get on. And we have a process where they are going to screen for those, if they find any of those correct them, and then reassess and see if it took care of the problem. Then if it didn’t take care of the problem we have reversible medical causes that we have them screen for, Ok, and check those. Anything off? Got a vitamin problem? Got your thyroid’s off? Correct it, see if it corrected the problem. These are all things that actually care are very good at because these are bread and butter medical issues. They are very good at this. Ok, you notice I have not said a word about diagnosis yet. Just you know detect things and correct them if you can. (P-Ex1)  Well one of the things that we’re…one of the things that I’m really, really pushing for with this beneficiary family Medicare is that the wellness visit, um, that the Mini Cog be part of the wellness visit and um, so that would help tremendously. That’s just a few minutes, four minutes. (P-CP2) |  |
| Value of seeing a specialist |  | So I mean and we made those recommendations in terms of you know referring patients to…for instance in the muscular dystrophy guidelines a couple of them are recommendations and said you know these patients can be complicated and before you jump into a whole bunch of stuff, maybe you should just uh…it was, they were nicely worded but we made those recommendations of having them see someone who’s experienced. So I don’t think those are out of the realm of possibility at all. (C-Mod)  In the MCI guideline that’s in preparation will make similar recommendations that you need to be thoroughly evaluated by someone, they don’t say by a dementia specialist, it says someone with experience in assess of cognition. It’s…I mean it’s kind of intuitive. (C-GDDI-Ex1)  You can be an experienced general neurologist (C-Ex1)  Absolutely (C-GDDI-Ex1)  So it’s sort of the… You should be seen by somebody who knows what they’re doing (C-Method)  And that’s a general principal here. (C-GDDI3) |
| Importance of pre-amyloid PET counseling | So one of the things on the recommendation phase is that we can have discussions about the sort of nuanced counseling things that we can incorporate that into that phase (P-GDDI-Ex1)  That’s very important (P-CP2) | in the appropriate use criteria, they also mention you need to counsel them about long term care insurance before ordering the test because that’s (C-Ex1)  Do you give any pretest counseling or is it necessary? (C-Mod)  Yes (C-Ex1)  That way the patients considering amyloid PET scanning does pretest counseling compared to no counseling improve the outcome in terms of how happy they are. (C-GDDI4)  I’m not sure you’re ever going to get someone, ethically, not to counsel. (C-Ex2)  In a test yes, but in real life they do it all the time. (C-GDDI4)  you said that most people will do some form of counseling so should we be looking at do you have references as to what that counseling, previous counseling would consist of. I mean should we counsel them about you know obviously about what the implication of a positive test and a negative test is uh differentiating AD versus ADD, differences…and then sort of what it means in terms of what the diagnosis actually mean, what a positive….so what would you do I mean we sort of have to define it in some way. (C-Mod)  I think the pretest counseling has been defined for the research studies so at best you could say uh if someone’s looked at it does the counseling in this study, how does that affect people Alzheimer’s result versus the counseling in another study rather than…I don’t think you’ll find within a study different counseling. (C-GDDI4)  Actually in terms of counseling other things to look at would be the efficacy of pretest counseling and people doing what they’ve been asked to do. So someone says Ok I’m bit forgetful, I want to have this test, well you better get long term care insurance. Do they actually get it. (C-GDDI4)  I think uh I guess what I’d say is everyone that say you have to counsel in the context of the support system and um I would imagine anyone would order a test on someone with cognitive issues by themselves. (C-Ex1)  I think counseling is going to be a really important recommendation to make because everyone in the room who would order the test has that perspective of how important it is but your PCP might just say Oh yeah we might do this test, see what’s causing your confusion and they might not, I mean not to say PCPs are bad but you know it’s not something they do all the time they don’t think through all these potential application, you know, having a set protocol or even on the dissemination when we get into that , having a patient resource you know. If you’re considering this what should you think about, you know. Maybe some issues that could be something really helpful for patients and families. (C-GDDI3)  I think counseling will be an important recommendation. I mean I think we’re going…but it’s not a question we’ll ask. I think it will…it will…we will infer, you know maybe patients ought to be told this about this test and I think it’s crucial, it’ll be probably be…probably half our recommendations will be counseling them so I think you’re right. But it won’t be something that we systematically review the evidence. Now when we get to the counseling, when they look at related evidence to help us decide how should we think this. But I don’t think we have…because it’s not specifically related to amyloid PET I don’t think we need to…Look what’s the best way to counsel these patients. Unless once the experts say No there’s some evidence or we disagree or. (C-Method)  We believe there’s a lot (inaudible) (C-Ex1)  That would be no different from genetic counseling for neurodegenerative diseases. I mean you need certain training to be able to do that and we all have disaster stories of people that have test done with no counseling. (C-GDDI4)  **Someone with experience should counsel**  Who typically does the counseling, you know like a genetic counselor does all that information, but is it the neurologist, the social worker, the nurse. (C-GDDI2)  I think the neurologist (C-Ex1)  Someone with experience… So I mean it could bee anyone in the care team because many of these patients are cared for in a multidisciplinary environment (C-Mod)  So you could ask the question was there appropriate training for someone to counsel patients with this (C-GDDI4)  **Do people get test after counseling**  Follow through rate, not in terms of did they do their homework but proportion of people that start actually get the test? (C-GDDI4)  After pretest counseling how many of them decide to get the test (C-Mod)  Right. Which actually might be much higher than it is in Huntington’s disease (C-GDDI4)  **Content of counseling**  So the Alzheimer’s Association put out a pre and post scan recommendations for education and counseling. So I’ll just tell you real quick. So they said discuss the clinical value and limitations of the test, assess patient motivation for tests and ability to accept results, discuss potential ethical and social issues, implications for driving, future employability, future insurability. And then post scan disclose the results with another person present in addition to the patient, consider the problem of automatic release of results so that the amyloid PET scan doesn’t go to the record before you see them, provide advice for the family, your care partner, discuss prognosis and plan for continuing care, develop a treatment plan incorporating community resources. So that’s what the Alzheimer’s Association put out. (C-Ex1)  That’s pretty comprehensive care. It would be interesting to know how much counseling actually gets into all that. It may be interesting to look at that. (C-Mod)  You’ll be able to research the literature, what counseling has been done in clinical trials but since it’s barely available commercially you probably won’t be able to do a true gap analysis in the community. (C-GDDI4) |
| Importance of how diagnosis is given/presented  **[This could have implications for counseling recommendations]**  **[Again, putting current guideline in broader perspective of patient care in this area]** | Also another very …a really important aspect of it... When a person receives the diagnosis is how is it presented. The how is very, very important because if the neurologist is supportive and can say it in a way that can be a safe diagnosis, if there’s such a thing for a patient and the caregiver that makes all the difference. We have an expert here on patient centered care, she just gives an outstanding…yes, yes she does, she’s amazing…I’ve taken her course twice just to learn how to be more communicative, politically correct and all the other things… there’s a way of saying things. And that is going to make a whole…a whole difference in terms of how a patient is going to take this diagnosis. What am I going to do with this amyloid diagnosis. Is the doctor going to say well, and we know 55% of communication is body language and 28% is (P-CP2)  38, tone of voice (P-Adv)  Oh 38 tone of voice, yes and 5% are words (P-CP2)  7… UCLA 1967 (P-Adv)  That’s a very important aspect of this… of getting the diagnosis, how it is presented (P-CP2) |  |
| Differences in wanting to know about diagnosis and prognosis | **Patient voicing wanting to know**  I feel that I own the information, I mean it should be my information (P-P1)  **Everyone is different**  hat I’m basically saying is that everyone’s coming from a different place with regard to this and the passion that some people will have from wanting this information is …it differs on many different levels. (P-CP2)  And I think in some ways from the Alzheimer’s associations perspective these are some unique individuals. Not everybody takes that position of wanting to know. (P-Adv)  **Discordance between patient and caregiver in desiring information**  And I totally respect P-P1’s comments and I think he’s probably at the top of the spectrum regarding really caring about his everyday life, but as we know and we hear everyday, if you’ve seen one case, one person, you’ve seen one person and heard one person because I’m thinking that my husband, the reason he’s not here, there’s nothing wrong with him, he’s just living his life just as it is day to day and he could care less about all of this. I am the one who is involved… (P-CP2)  When we have discussions about obtaining additional biomarkers for prognostic purposes there’s often discordance actually between the two individuals, between the patient and the caregiver or informant in terms of who wants that additional information  **Right to know or not to know (with caveat)**  The way I have sort of synthesized these two is that as a patient, you have the right to know and you have the right not to know. But when it comes to dementia I think if you choose the right not to know you have to designate someone to know for you… (P-Ex1)  Well no and it just goes back to what you said earlier about that the patient has the right to know, the patient has the right not to know, but if you pick B this somebody has to know so how do you close that loop up (P-Adv)  **Someone should know**  …When it comes to dementia I think if you choose the right not to know you have to designate someone to know for you so that someone can help interface with the health care team to make sure you’re safe. (P-Ex1)  Yes (P-P1)  Excellent (P-CP2)  And I…to your point I think somebody needs to know that doctor I don’t think can withhold it from either the caregiver family member or the person. I mean somebody needs to know that. (P-P1)  Well no and it just goes back to what you said earlier about that the patient has the right to know, the patient has the right not to know, but if you pick B this somebody has to know so how do you close that loop up and if there would be again something more definitive or more defining in terms of the process, does that make it, I don’t know, easier to deliver that information, does that make it more likely that if the patient says no I don’t want to know but they tell the caregiver that the caregiver is like absolutely I agree, I mean what is the cause and effect of that whole process. (P-Adv)  These are real safety issues. Yeah you may not want to know but if you get behind a wheel and kill someone, you didn’t have the right. Um, if you set your house on fire and kill people in that house, you didn’t have that right. These are important real issues and you would be surprised how many people because the social brain may be hit later than other parts, maybe able to put on a brave front and just on casual conversation you’d never notice. (P-Ex1) |  |
| Importance of engaging spouse/ caregiver | **Caregiver active in medical visits**  The one idea I think that comes out is who’s the patient. Uh because at a certain point in the…it’s transitions to being the family caregiver is really who becomes the person who…who you’re conversing with them and that group of people are very much affected by the disease (P-GDDI-Ex1)  **Caregiver describing husband’s lack of awareness**  I’m thinking that my husband, the reason he’s not here, there’s nothing wrong with him (P-CP2)  No as I said before, there are many others in my situation and um…but…it’s very interesting to have so many people at our church or social situations, most of our best friends have Alzheimer’s, that’s our community. But outside of the community who say you know I don’t really think he has it. He’s very smooth, very, very smooth. And he can socialize very well. How are you doing buddy, how are you doing sweetie. Never Do I know them? You know it will come out, we have somebody new living down the hall from us. I see all of these other behaviors that other’s don’t but I’m not the only one. I mean yeah, I’m sitting here and I’m frustrated and I…I take mega doses of antidepressants and I, you know I’ve a long list of symptoms of being a caregiver but I’m not the only one. Just one that you’re hearing about today. (P-CP2)  **Can be hard to recognize dementia in people socially**  You would be surprised how many people because the social brain may be hit later than other parts, maybe able to put on a brave front and just on casual conversation you’d never notice. (P-Ex1)  Right (P-CP2)  **Patient recognizing caregiver role**  Yes, actually yeah the caregiver and the patient have to be almost inseparable I mean in a sense that if they are not function well together that’s a huge problem. (P-P1) | **Spouse needed for accurate history**  In the memory clinic we take this…I insist on the spouse being there for the appointment or I cancel the appointment. So I take asymptomatic…I don’t listen to the patient. When anosognosia is a core feature of the disease that you’re trying to diagnose, I don’t take the patient…the patient says they have no symptoms that add a whole lot of value and we could look at that, I don’t know how. I’m not sure how that pans out. (C-GDDI-Ex1)  It panned out in the driving stuff and that…if either said that they were a good drivers it doesn’t matter if the spouse said that they were a bad driver, they probably were. (C-GDDI4) |
| Long term care (not as benefit or harm) | This is the problem with long term care and *** and I have given several talks at long term care conferences and uh we’ve been very open about the fine print and how we would really choose not to get it and we actually chose not to get it initially years ago which is very too bad on one hand, but as it turns out um, well and that’s a whole other story but even now we’re now in…because of our journey through this we are…we are um, not eligible financially to get long term care or any type of care. I’m going to be the caregiver, we are going to be in the Medicaid um, lane in the Twin Cities there are three homes, residences that offer Medicaid from home to a facility. Three. (P-CP2)  That are not skilled nursing (P-Adv)  Just where he needs memory care (P-CP2)  Yeah so there’s two levels of…so like assisted living although it’s not called that in Minnesota, there’s very limited access if you’re on Medicaid. Most of them, the vast majority are private pay. (P-Adv)  For two years and then they may or may not allow you to come in for Medicaid but we’re not eligible for…to even begin to talk about that. (P-CP2) |  |
| Patients and physicians may have different views on benefit of testing |  | There might be some benefit to…there might be a patient driven thing. I have some patient who when I said I’m…by my numbers you…I’m 80% certain that you have…in the patient population like this when I say you have Alzheimer’s disease dementia as a pathology, my colleagues and I are 80 to 85% correct and that’s about as good as we’re going to get because there’s a whole lot of host of other pathologies that look just like Alzheimer’s disease dementia and some of my patients still say it’s because that IDEAS study that they are eligible they still say well I want to get the PET scan and I’ll explain to them the, you know the reason that scan could say you have Alzheimer’s disease and you still are not necessarily going to have Alzheimer’s disease dementia. You have another pathology in there that we don’t get it and they’ll still want it. So it may be patient driven that we…that we do it in those groups if it is paid for. (C-GDDI-Ex1) |
| Reasons it is important to have a diagnosis  *MUCH more time in the transcript/ meeting was spent on this topic in Group 1 than Group 2* | **Importance of a diagnosis in general**  I think you’re far better… should be suited to deal with that issue the earlier you hear it (P-P1)  I always want a diagnosis. I’m funny that way. (P-Ex1)  I am, too. I think this conversation is so important because there’s so many… it’s not cut and dried. (P-CP2)  Just understanding dementia. Because I know a lot of people even if they can’t do anything they just want to understand what’s going on. (P-Ex1)  So what I’m hearing from a quality of life, quality of care perspective is for you all, you really do…you want to know…you want to be able to detect if you’re at risk for developing a dementia and you want to know what type of dementia that either you’re at risk for or that you have. Is that accurate? And that that information is important to you both for your peace of mind and for planning your life and your quality of life. (P-Ex1)  And care (P-CP2)  I mean it is really helpful having you here to hear an underlying…the decreased uncertainty, increased confidence in diagnosis and prognosis is so important, you know as doctors we always like well we don’t want to do a test if it’s not going to change management, if there’s not a specific therapy that we’re going to offer based on that test. We’ve very much been taught t like cut that out and uh, so I think that’s great to hear that underlying that… (P-Moderator)  We are benefited by participation of the representatives who told a story, P-CP2 about how frustrating it has been to not have the right answer as to whether this is Alzheimer’s or not. I don’t know. That seems like the benefit. (P-Mod)  Frustrating is just one of the words (P-CP2)  I think this is sort of…it’s been an interesting conversation with you all about how much the label matters and I do think labels matter to people, words matter to people, membership matters to people and so it’s very blithely…you’re not going to be very blithe to sort of say, um, you know it’s all about whether or not in 5 years you’re going to have functional decline, but people…I think people still want to know what is the disease… (P-Ex2)  They want to name it (P-Adv)  …What is the disease I can say I have when my friend or neighbor comes up to me and says I have it so I, uh, I think that that’s important. (P-Ex2)  So again we’ve said a number of times that the actual decision about an Alzheimer’s diagnosis is important for the patients, the patients and outcomes are important. So whether or not you separate it out, somewhere among these seven there should be some reflection of that importance. (P-GDDI-Ex1)  *Provides closure*  So I will be very relieved to have some closure on what is happening with his brain because on a day to day basis, um, life is very challenging. (P-CP2)  *Support is less when diagnosis is unknown*  I think the underlying issue for…as a caregiver is, how do we receive the support from our medical community who you know, rightfully so…I’m a speech pathologist, I know how we go through diagnostic tests and so forth but how do we support the patients with this uncertainty. So there’s just another layer. (P-CP1)  **Validation that something is wrong**  I’ll tell you having spent…having gone to 7 doctors before *** was kind of diagnosed, it’s better to know than not to know than not to know. When you know that something is…there are changes and I don’t want to use the word wrong because my husband really, really objects, there’s nothing wrong with me, there were changes in his behavior and I have to respect that in terms of his mental health and well being but there are so many who know that there’s something and yet aren’t told. So if a doctor knows, all right here we have this Tau, I mean this amyloid imaging, that may be a reason…we can get some answers and maybe others will know because there are many who are living in this twilight zone. Something is not normal. (P-CP2)  No as I said before, there are many others in my situation and um…but…it’s very interesting to have so many people at our church or social situations, most of our best friends have Alzheimer’s, that’s our community. But outside of the community who say you know I don’t really think he has it. He’s very smooth, very, very smooth. And he can socialize very well. How are you doing buddy, how are you doing sweetie. Never Do I know them? You know it will come out, we have somebody new living down the hall from us. I see all of these other behaviors that other’s don’t but I’m not the only one. I mean yeah, I’m sitting here and I’m frustrated and I…I take mega doses of antidepressants and I, you know I’ve a long list of symptoms of being a caregiver but I’m not the only one. Just one that you’re hearing about today. We have a whole (P-CP2)  **Addressing uncertainty, different diagnoses**  He was diagnosed with Alzheimer’s in 2009, um, early stage Alzheimer’s. Subsequently he’s been diagnosed with MCI, subsequently with vascular dementia. Subsequently he has had a spinal tap, the PET scan, the PAT F18, um, MRI, a blood test. He’s APOE3 rather than 4. I have his file here. Um, and most recently the doctor said I’m rather certain its not Alzheimer’s and I don’t know why you’re here. And I fell apart… (P-CP2)  In my conversation with people in the Alzheimer’s Association with the early stage there are many, many who have the same mixed, not only kind of nondescript diagnosis where it could be frontal lobe, it could be vascular, it could be this, it could be that. (P-CP1)  So I’m thinking in a more certainty sounds like you guys have gone through a lot of uncertainty with various tests and doctors and things. So reduction of uncertainty about diagnosis might be something we might want to get out of it, right? (P-GDDI-Ex1)  All right, so again listing potential benefits that we might look for in the literature for the PICO outcomes, reducing uncertainty was spoke of many times this morning as being very beneficial to patients. (P-Moderator)  **Reducing other testing**  If you’re also thinking about paraneoplastic things, other things, and so um, it may reduce the um, the number of tests that we need to get um, so (P-Ex2)  I would add for…I think under the um, early/young onset cases there probably is some reduction of unnecessary testing, um, for clinical based on… (P-Ex2)  **Importance for prognosis**  I guess my point is in prodromal Alzheimer’s Disease or cognitive impairment stage what patients usually want to know is what’s my risk that this is going to go on into Alzheimer’s Disease and so we are not…given that modeled cognitive impairment has moved into the clinical demand, people are getting diagnosed with that, um, routinely in primary care offices and otherwise, um, being able to provide more certainty and what that means for that individual is you know critical.  But I think to have something like that, that would be more readily accessible to deal with you know what is the prognosis, where can you deal with the uncertainties for planning because people once they do know, a lot of times are more receptive the more definitive that plan is. Where they kind of know, Ok here’s…yeah we don’t know exactly how this is going to happen but this is going to get worse. They want to know that information. (P-Adv)  I do also though hope that and think that there is value in prognosis that we as a society value prognosis of all sorts of diseases like even if you have a cancer diagnosis that’s incurable you want to know, you know, how long you are going to live for and so my suspicion is that there will…even if there weren’t some of these treatments available there will be a movement towards using this for prognostic purposes in the future. (P-Ex2)  **Links to services, support**  So another piece though to add then would be to, with a more definitive diagnosis to link people to services and support would be another goal. (C-GDDI-Ex1)  So if they’re starting to have problems, knowing and being able to prepare for it and being able to get them resources and support is extremely important. (P-Ex1)  And then with the diagnosis you refer them to the Alzheimer’s Association so they can learn, be educated, learn about it if there doesn’t happen to be a multi disciplinary support at the doctor’s office, or the clinic, refer them to the Alzheimer’s Association and they in turn teach us to live well. And so there’s a positive aspect to… (P-CP2)  Very definitely (P-P1)  …there is life after diagnosis and we have social…a social community and we support each other and we become involved in support groups which keeps us all going, especially the caregiver. (P-CP2)  Yeah, that’s huge (P-P1)  **Diagnosis allows people to better plan their lives**  I guess one thing I kind of…on there from the patient’s side, learn, you know, what can that knowing what can that impact on living with. Because I mean if I have a better idea of where this is going that helps me plan my life. (P-P1)  And that goes for the caregiver as well. (P-CP1)  Not just the patient but everybody around them, the patient. (P-Mod)  Yes, actually yeah the caregiver and the patient have to be almost inseparable I mean in a sense that if they are not function well together that’s a huge problem. (P-P1)  I guess yeah…I feel that I own the information, I mean it should be my information so I think for me you know you can talk about the pathology but I have a life to live and the more information I can get about what are my likely paths for my life, that allows me more control over my life and so how can you translate the imaging and the other techniques to help me plan my future life. It’s kind of like a business, you know, you want to know the economic climate you’re in, what are your resources, what’s your prognosis and so I’ve got to plan my life based on what you can tell me. (P-P1)  *Quote also included under treatment*  Planning, I mean you know I go to an investment advisor to help me plan how my investments are going to go. I go to my doctor to help me…How’s my health going to be. So I mean those are things that we have to know. (P-P1)  Ok, so that’s a benefit. It encourages long term planning. (P-Ex1)  But if you plan correctly and of course this is giving the assumption that you have somebody that you can say if you save that money yourself and invested it you could probably have a better plan than a long term care insurance. (P-CP1)  Potentially (P-Ex1)  If you started early enough just like all the ads talk about, you know at age 20 if you put away $1,000 a year versus you know starting…put away $1,000 a year at age 40, where are you going to end up with at age 65. I mean it can be that same kind of a scenario, but if that would encourage you to do that long term planning even if it’s personal and it’s not through an actually planning block, if it’s through your 401k or whatever, if you have access to that, then there is the benefit of the amyloid for a pre-clinical. (P-CP1)  Unless you have frontal lobe dementia and you have put your family in bankruptcy. (P-CP2)  *Long term care*  And then the mirror question would be what are the benefits, like getting more people to sign up for long term care. (P-Ex1)  **Advance planning**  Potentially is encourage more people to do advance planning. (P-Ex1)  *Importance of having an early diagnosis for planning*  I guess I’m thinking about that because when P-P1 had…he’s in a trial match and when I was talking to the neuro psychologist with a bunch of questions and we got to talking about early diagnosis and because we see him at the Alzheimer’s and various other places and he said Yeah. He said the hardest part about my job is that is was so discouraging last week, in fact he said every patient I saw referred to me, the physician is saying does this person have dementia? And he said every patient I saw with that I knew in 30 seconds that they were quite away along, they weren’t in mild cognitive impairment. They weren’t in just early diagnosis, they were further along. So you know while I think that this is really important for the neurologist to be looking at, how is it going to impact the life of the patient early enough that they get the help in living with the disease. (P-CP1)  I think my life is extremely improved by having that early diagnosis. And so I mean that’s where I think you back that back to how can we get to that earlier diagnosis or just channeling say this is something to check up. (P-P1)  **Access to disability/employment protection**  If you choose to not diagnose…or if you choose to not disclose though and so in an employment situation for example and you start to have some challenges and end up loosing your job, but you didn’t tell him that there was an issue, now there’s no protection. So I mean that could certainly be something and we have had clients that have been in that situation where they did not share that information and there was a change in their ability to work and the employer didn’t know, they lost their job to the point earlier, about yeah this was a younger person, kids at home carrying benefits, I mean there you’ve given to that whole…because with that younger onset that’s a whole other animal versus that 80 something. (P-Adv)  **Safety planning**  But when it comes to dementia I think if you choose the right not to know you have to designate someone to know for you so that someone can help interface with the health care team to make sure you’re safe. (P-Ex1)  These are real safety issues. Yeah you may not want to know but if you get behind a wheel and kill someone, you didn’t have the right. Um, if you set your house on fire and kill people in that house, you didn’t have that right. (P-Ex1)  **Diagnosis impacts treatment**  The question really is, Ok can you answer questions that will help me live a better life or you know help me reduce the impact of the disease? So I mean part of it is just tell me I…you know I have six months to live or six years. And the other one is, you know, what is the progression going to be and is there anything I can do to intervene in that? But it…and I just sense I’m going to repeat myself. I’m going to demonstrate I have this disease, but I mean how do I, you know, both treat it and live with it? (P-P1)  And or you know, management? (P-Mod)  I’d go on to say it’s not only can you recognize it say in your spouse, I can recognize what I think is this behavior in other people just in group settings and so I mean there is a certain, well you use the term pathology, I mean there are certain characteristics of the behavior that I think you start to just recognize in people around you and you feel like going to people, I think you ought to go get checked. I mean because really it’s…and if we can send out the message that getting checked, getting on medication slows the progression. You want to find this out instead of this is a death sentence, you will not…you know be able to function in six months. If you go our there and say you know we can help treat this, that’s when you start getting the people out of the woodwork. I want to get on this treatment. (P-P1)  Knowing amyloid status might push you more towards wanting to treat with Alzheimer’s disease drugs, there’s some that at least that acetylcholine inhibitors, one of the main drugs like Aricept that we use in Alzheimer’s actually has negative effects in frontal temporal dementia causing behavioral um symptoms. (P-Ex2)  **Diagnosis impacts care more generally**  And care (P-CP2)  We really try to encourage people you know to get identified early, to go to the doctor if there is a problem, we’d like to see physicians doing the…with the wellness exams, doing the cognitive screens at wellness exams, if this was another mechanism where they would get a more definitive picture sooner, I think that would…it would certainly help us to tighten that loop up a little bit so that they would get into the system sooner, get the support that they need, be in the trials, use the treatments. I mean all of those things start feeding one another. (P-Adv)  **Gives control**  I guess yeah…I feel that I own the information, I mean it should be my information so I think for me you know you can talk about the pathology but I have a life to live and the more information I can get about what are my likely paths for my life, that allows me more control over my life and so how can you translate the imaging and the other techniques to help me plan my future life. It’s kind of like a business, you know, you want to know the economic climate you’re in, what are your resources, what’s your prognosis and so I’ve got to plan my life based on what you can tell me. (P-P1)  **Access to research**  How about access to research… So if you know you’re pre-clinical it might get you involved in clinical research which helps…just being involved in clinical research can have a positive outcomes so even if you are in a placebo group, uh getting early access to potentially risk modifying treatments and helping society which some people get value out of, you know to understand and fight these diseases. (P-Ex1) | **Benefit of knowing in general**  So first there’s the benefit or harm of knowing, just knowing. (C-Method)  One of the questions is you know do people want to know if the have Alzheimer’s disease and that’s been looked at and they overwhelmingly do. Over 90% of people when asked want to know. (C-Ex1)  So I wonder if…how mature that literature is. I just don’t know when I’m sitting next to a Huntington’s disease expert and I think when you first ask the question to somebody at risk for Huntington’s disease that many of them say Yes I want to know but then when you sort of explain to them the implications and what it means, then I think a large chunk of them and maybe the majority don’t want to know. (C-Method)  70% versus 4% (C-GDDI4)  But there is sort of that, the initial reaction is yes, but then they think about it, the answer is no. Have they…Have they (C-Method)  But these are…the difference there being these are people at risk for Huntington’s disease. We’re talking about patients who already, they’re the…usually the ones that…they and their loved ones are the ones complaining that they have a disease just no one has spoken what it is. So it’s sort of like walking around with hemiparesis and saying I don’t want to know what caused this. (C-GDDI-Ex1)  *Desire to know may be different in pre-symptomatic individuals*  A good analogy to the Huntington’s would be someone at risk of Alzheimer’s disease saying what is my status. Not the first one. So I don’t know if that questions has been asked as much. (C-Ex1)  But that’s been answered through some of the autosome dominate and the (inaudible) studies but the autosomal AD which is very analogous to Huntington’s disease. We’ve got kids of parents who have the same 50% risk of inheriting that causal gene and the DIANE trial, DIANE study offers that ability to the kids to find out and I have to find the percentages for which of them take it with counseling but it’s a pretty high percentage but that’s different in a way because they have access to the potential treatment trial so if they (C-GDDI-Ex1)  … I have to pull up the exact references but um the same for the family members they want to know too. (C-Ex1)  So that’s a good…potentially a good question… Do the patients want to know (C-Method)  And then the impact on them after they find out the answer. In terms of behavior, quality of life, uh I mean every domain you can think of, employment, uh independence, social interactions. (C-GDDI4)  Well the way that you explained it to me the reason that they commonly say no when they are pre-symptomatic is because everything that they notice then becomes Oh my god this is the first…and I can see that being prevalent you know if you’re in…of course this would be the asymptomatic, but maybe even somebody with MCI, any blocking for coming up with their 2nd cousins name it then becomes a big deal when before you’d say Ah just getting a little bit forgetful. (C-Method)  **Addressing uncertainty**  All of these things we listed under harms there are potential benefits, qualify of life and patients actually…patients want to…usually by the time they are in my clinic they want to know, they know there’s a problem they want to know what it is and if I don’t tell them they’ll see another neurologist, they’ll get another FDG PET, they might have CSF done and analyzed and… (C-GDDI-Ex1)  **Family wants to know**  And it’s not just the patient, it’s the family (C-GDDI-Ex2)  **Reducing other testing**  They know there’s a problem they want to know what it is and if I don’t tell them they’ll see another neurologist, they’ll get another FDG PET, the might have CSF done and analyzed and (C-GDDI-Ex1)  What about…is there a way to look at ordering less tests downstream? (C-Ex1)  So (talk over)… That’s the benefit of knowing and so it’s, yeah looking for a…looking for or not looking for alternative causes, it’s potentially a harm or benefit. If it’s a false positive it’s a harm because you’re not looking when you should be, um, but yes that’s important… Do we have pictures of all this? (C-Method)  **Diagnosis allows people to better plan their lives**  Care planning (C-GDDI3)  Planning against… future planning…It would be interesting I think to know how they use it for planning. I mean, does that include buying long term insurance? (C-Mod)  You have the social consequences listed, right? The insurance (talk over) (C-Method)  Yes. Work, financial (talk over)… There’s two lists of social consequences (C-Mod)  Besides long term care insurance do we have advance directive planning and that kind of stuff for this? (C-GDDI3)  So, preparations. (C-Mod)  …I’ve got that as a separate one although you can argue where it goes. Counseling regarding hospitalization, you know living wills and we can probably list more. And then there are social consequences which would be harms or benefits and you can argue whether it’s related or…so employment, driving, financial independence. Ok? (C-Method)  *Long term care*  Long term care (C-Mod)  **Advanced planning**  DPOA [durable power of attorney] would go under anticipated (C-GDDI4)  Living will, DPOA (C-Method)  **Access to disability/employment protection**  It’s important for people that are working because I’ve seen people get fired or they don’t get their long term disability but actually they had posterior cortical atrophy and so the reason they are fired is because they couldn’t manipulate their spreadsheet but it’s due to a disease not to their laziness or incompetence and if they had known earlier…I’ve counseled people that you have a disease and you need to be accessing what you’re entitled to so that you don’t…your employer doesn’t build a case against you that says you’re unable to do your job, we’ve fired you and you don’t qualify for disability. (C-GDDI-Ex1)  But the diagnosis occurs after so they won’t retrospectively (talk over) (C-Ex1)  That’s right. So that’s a big issue in some of the younger (C-GDDI-Ex1)  I have a physician like that (C-Ex1)  **Diagnosis impacts treatment**  Our underlying assumption is that we can do something about it whether it’s appropriate treatment or potential for new therapy (talk over) (C-GDDI3)  I think there’s enough evidence that in you know like distinguishing AD from FTD, diagnosing dementia early there’s enough benefits that we can make those things (C-Ex1)  So you sort of follow through after diagnosis, so, “what now?” even if there isn’t a specific treatment. (C-Mod)  **Reducing hospitalization**  What I do is when I’ve made a diagnosis I talk to them about the risks of hospitalization in someone who has dementia. (C-Ex1)  Oh, in terms of preparing for it. Got it. (C-Method)  And so I say you know sometimes you have to go to the hospital if you have chest pain and those things but if there’s things that can be managed urgently as an out patient, do whatever you can to make sure that’s done and you try and prevent them because once they get hospitalized the expenses go astronomical and the amyloid PET is the last little drop in the bucket because they are there for weeks. (C-Ex1)  If you say you have Alzheimer’s disease, you know, does it reduce hospitalization by 10%? (C-Ex1)  It doesn’t hold for only Alzheimer’s disease, alright, it holds for most dementias (C-Mod)  **Summary of advantages of testing**  So all these other outcomes are what is the advantage of knowing that you’ve got Alzheimer’s disease. What is the advantage if you’re not certain of looking for alternative causes or the harms, what are the advantages of early treatment, what are the advantages of the anticipating preparing for Alzheimer’s disease, what are the advantages of dealing with these social consequences, Ok. These questions are not specific to amyloid PET, Ok. These questions are specific to whether you have dementia or you have Alzheimer’s disease. (C-Method) |
| Some things focus on importance of diagnosis only for treatment | you know as doctors we always like well we don’t want to do a test if it’s not going to change management, if there’s not a specific therapy that we’re going to offer based on that test. (P-Mod) | But the appropriate use criteria is all based on the premise that we don’t have a treatment for AD where as our guideline doesn’t have to be based on that premise. (C-GDDI-Ex1) |
| Value of patient view in discussions | It is really helpful having you here to hear an underlying…the decreased uncertainty, increased confidence in diagnosis and prognosis is so important, you know as doctors we always like well we don’t want to do a test if it’s not going to change management, if there’s not a specific therapy that we’re going to offer based on that test. We’ve very much been taught t like cut that out and uh, so I think that’s great to hear that underlying that… (P-Mod)  So one of the things on the recommendation phase is that we can have discussions about the sort of nuanced counseling things that we can incorporate that into that phase (P-GDDI-Ex1)  That’s very important (P-CP2)  So it’s especially a critical element and often comes from you guys (P-GDDI-Ex1)  Yes, very important (P-CP2)  Ok what…where would people that have traumatic brain injury, concussions, where they fit in? Should they fit into this risk category? (P-Adv)  Boy, that’s a loaded question (P-Ex1)  Well it’s a very good question (P-Mod) |  |

Table 3. Disagreements present within transcript

| Theme | Gp 1 (Exper, WITH PATIENTS) | Gp 2 (Control, PHYSICIANS ONLY) |
| --- | --- | --- |
| Disagreement between patient representatives | I guess yeah…I feel that I own the information, I mean it should be my information so I think for me you know you can talk about the pathology but I have a life to live and the more information I can get about what are my likely paths for my life, that allows me more control over my life and so how can you translate the imaging and the other techniques to help me plan my future life. It’s kind of like a business, you know, you want to know the economic climate you’re in, what are your resources, what’s your prognosis and so I’ve got to plan my life based on what you can tell me. (P-P1)  And I totally respect P-P1’s comments and I think he’s probably at the top of the spectrum regarding really caring about his everyday life, but as we know and we hear everyday, if you’ve seen one case, one person, you’ve seen one person and heard one person because I’m thinking that my husband, the reason he’s not here, there’s nothing wrong with him, he’s just living his life just as it is day to day and he could care less about all of this. I am the one who is involved… (P-CP2) |  |
| Disagreement between physician representatives | Well *** I think we’ve got to be careful when we’re saying those numbers. So when you look at, you know regular diagnosis in the community, that’s on that 50% spectrum. When you look dedicated memory centers that’s where you’re getting up to that 90-95%. (P-Ex1)  Yeah I disagree actually I think that much of that data is based on autopsy based studies… (P-Ex2)  I think just…I think there’s um, sort of equipoise about whether…I don’t think, I don’t know if it’s clear that there are people that have amyloid who would not get Alzheimer’s disease if they life long enough to get the disease. (P-Ex2)  I’m going to disagree with that statement. I think we’ve got enough from the religious order study to say there are some people. Unless you’re talking about in their hundreds. (P-Ex1)  That’s all we’re measuring. That’s all we’re going to measure is cognitive impairment. We will have that answer and how you interpret that whether you’re going to call it Alzheimer’s disease or you’re going to say its probably Alzheimer’s disease. Nobody cares. If the only thing that matters is that we have the cognitive impairment answer for patients, right? (P-Moderator)  And I don’t like it but I will bow to the wisdom of the group. (P-Ex1) |  |

Table 4. Themes and Exemplary Quotes Relating to Implications of Amyloid Testing

| Theme | Gp 1 (Exper, WITH PATIENTS) | Gp 2 (Control, PHYSICIANS ONLY) |
| --- | --- | --- |
| Potential benefits of amyloid testing  ***[See separate section about benefits of having a diagnosis in general]*** | **Doctors may be more willing to diagnose**  Going back to my simplistic thinking, um, in terms of the positives for the imaging I mentioned um, more diagnoses so that doctors would be more cognizant and willing to diagnose (P-CP2)’  **Spur more research**  Also I am thinking that in looking at all of these brain images, hopefully it will help more with research that there will be more information about the amyloids that will help determine more about the disease and other dementias. (P-CP2)  **Informs prognosis**  So I think in that…sort of to amplify the uncertainty the other big issue is prognosis, um, which is um I think where amyloid imaging potentially has its greatest value particularly in the context right now of um, not having real disease modifying medications and so um, sort of knowing what to expect over the next 5-10 years is important and amyloid imaging can have a place for that. | **Allows preparation from AD**  And then anticipate and preparation for Alzheimer’s disease.  **Negative scan prompts additional evaluations**  We’re not going to find that and so can we find the evidence that it’s diagnostically accurate and uh and then can we find the evidence that you know if there’s no amyloid that looking for something else has a reasonable yield to improve their outcome so that you can connect the dots and some up with… (C-Method)  So can it enhance our ability to detect reversible treatable… (C-Ex1)  And it’s an obvious need in the future and the more testing that we do, the more this cohort that C-Method, you’re after we’ll build. You’ll have more and more people that are negative, then they could still have bad clinicians. (C-GDDI-Ex1)  You get the patient with the negative test that you though had Alzheimer’s dementia you then dig deeper to uncover potential treatable alternatives. So those are directly meaningful patient outcomes that kind of seem like harms in a way you know but actually it could be helpful… If there is a treatable alternative cause that you uncover because you got the negative test and realized Oh we need to look further. There’s something else happening. (C-GDDI3)  You’ve got the benefit of looking for alternative causes. (C-Method)  **Avoiding unnecessary treatment**  And these are all people that in the clinicians office are leaving on donepezil and other cholinesterase inhibitors… But meaning in a negative scan and not population could really actually be helpful in avoiding potential harms or unnecessary treatment… (C-GDDI-Ex1)  The potential patient benefit of avoiding unnecessary treatments you know patients that come in inappropriately on mematine or whatever um is something that you know a patient has a negative beta amyloid thre not going to be treated inaccurately with…and all the side effects and implications of that. (C-GDDI3)  **Getting early treatment**  I mean there is some debate about…I mean there is some evidence from a couple of randomized control trials that in MCI you put someone on a cholinesterase inhibitor, you can slow the progression of the disease. It’s not great evidence but it’s not like…but it is a potential benefit. Early treatment. (C-Method) |
| Discussion of implications of abnormal test in someone with normal cognition | So, it also sort of brings up the potential harm, misdiagnosis or misprognosis if you want to talk about the pre-clinical, the otential harm in that gets to well just how good a test it is (P-Ex1)  Because you have the amyloid that doesn’t go into dementia. There’s no way of predicting that. (P-CP1)  I think just…I think there’s um, sort of equipoise about whether…I don’t think, I don’t know if it’s clear that there are people that have amyloid who would not get Alzheimer’s disease if they life long enough to get the disease. (P-Ex2)  I’m going to disagree with that statement. I think we’ve got enough from the religious order study to say there are some people. Unless you’re talking about in their hundreds. (P-Ex1)  I’m not suggesting that it’s unknown answer. I’m just saying that there are plenty of people who die with evidence of Alzheimer’s pathology in their brain. The question is would they have developed symptoms if they lived long enough and we don’t know in an autopsy based study how long they’ve had amyloid for and to what degree they’ve had associated memory deterioration with it. I think it’s unknown. I mean this is why longitudinally having a marker like this in following someone, are there people…and maybe there are, who have amyloid for 30 years. We have patients in our cohort that I’ve got CSF studies in the 90s that haven’t yet developed cognitive impairment with evidence of amyloid. Now maybe that CSF test that we did wasn’t, you know accurate or whatever, but um, I think it’s unknown. You know and there probably are modulators that are like who’s more likely to develop (P-Ex2)  Amyloid is probably necessary but not um, sufficient for the development of Alzheimer’s dementia is my suspicion but we need more research to prove that. (P-Ex1) | There is a high percentage of people over 75 who are cognitively normal will have an abnormal PET scan. 25% or something… and all evidence will point to the fact that they have a higher risk if they live long enough to getting Alzheimer’s dementia but they can be cognitively normal with a positive amyloid PET. (C-Ex1) |
| Future of amyloid testing | **Amyloid imaging will be more important if there is an amyloid-based therapy**  It is a bold, um, bold questions. I mean you know I think a whole lot of um, so much of how we’re going to use amyloid imaging will be dependent on what drugs are available so I mean if there are drugs, you know there are various drugs in the pipeline, one that will be completing it’s um, another phase three trial (inaudible possibly drug name) in the very near future in mild Alzheimer’s disease as well as in mild cognitive impairment that is very targeted towards a beta and so if that is a positive study um, then you know we will be using amyloid imaging quite a bit in people with very mild symptoms and those studies seem to be…you know there’s also…we’re beginning a phase 3 study with another opinion based drug that’s directed at amyloid developed by BioGen and it seems like the models for those studies are that they are much more effective is you get amyloid imaging to define the group that you’re studying to increase diagnostic accuracy. So if those studies are designed in that manner then that, then amyloid imaging will inevitably be part of the criteria in clinical practice to use it. I do also though hope that and think that there is value in prognosis that we as a society value prognosis of all sorts of diseases like even if you have a cancer diagnosis that’s incurable you want to know, you know, how long you are going to live for and so my suspicion is that there will…even if there weren’t some of these treatments available there will be a movement towards using this for prognostic purposes in the future. (P-Ex2)  If an amyloid targeted treatment proves itself effective at any stage. It could be pre…pre MCI, it could be MCI, it could be Alzheimer’s, then amyloid imaging will actually become very important for clinical practice. (P-Ex1)  **Amyloid imaging will be supplanted by tau imaging**  I predict that 10 years from now in terms of uh you know symptomatic folks at the very least Tau imaging is going to supplant amyloid imaging. So I don’t think it’s going to be used in that domain. I think that it may, but I don’t know this, even to prediction, you know. If you’re going to do a…you know a detection modality targeting amyloid pathology or Alzheimer’s pathology I think Tau is going to supplant it because Tau is much more…kind of goes with where the damage is in Alzheimer’s… I’m still very concerned that 10 years from now we will not have a treatment directed at amyloid for anything just because of the fact that it’s just…that nothing has really stood out… (P-Ex1) |  |
| Limitations of test | **It’s not destiny**  And it’s only if you’re going to get worse and then the answer is maybe, you know if definitely will, some of the people with pre-clinical amyloid it puts you in a higher risk for developing Alzheimer’s disease, but not necessarily destiny. (P-Ex1)  **Other tests may better predict progression**  For predicting like in a mild cognitive impairment patient whether in 5 years you’re going to develop more significant cognitive symptoms. I think there’s…it’s not a clear cut victory for amyloid. I think that there’s a lot of data that, markers of nerve degenerative change maybe even better monitors of…they might not tell you really what disease the person has or they are more predictive of outcomes which you know as we were talking about earlier that might be what’s more important to (P-Ex2)  But again if the only thing you cared about was getting dementia and you didn’t care about what type, you’d get a marker of neuronal injury. (P-Ex1)  I mean I actually I mean I would say from a um…just from an educational standpoint, um, that this comparisons is useful for people to know about because I do think there’s a misconception that amyloid PET tracks the disease. I think what we would find if you looked at the literature is that amongst um, Alzheimer’s disease patients markers of neuro degeneration better predict change overtime than amyloid does and so I think from a standpoint of providing new information for people that I can imagine a useful way of framing the question. (P-Ex2)  **Doesn’t tell you risk of non-amyloid cognitive impairment**  It tells you nothing about your risk of developing other cognitive impairments. (P-Ex1)  **Tells you about kind of dementia, not stage**  I think what’s the value of more for…so in amyloid one of the things that’s complicated about amyloid and I guess when you…when we start delving into the literature depending on the question we want is it’s not a very good marker of where people are in the disease um, so there’s a bit of a sort of plateauing of amyloid in the brain probably in the mild cognitive impair stage. But even more complicated than that people seem to plateau at different levels so you can’t just look at someone’s amyloid in the brain and say you’re at this stage and you have this far before you’re going to develop symptoms and so some of these other markers as complements, um, to amyloid may provide more precision and knowing the sort of time core so Tau imaging is one possibility but there are others. (P-Ex2) | **Amyloid deposition is common with aging and non-AD processes**  Yeah I mean getting to the false positive, um one of the issues with amyloid imaging is you know it can’t be substituted for the history because the DLB second most common cause of dementia, 60% will be positive on amyloid imaging so if you’re…you can’t answer the question is the AD or DLB because it’s going to be positive. (C-Ex1)  And even getting back to your original point that because there’s so many people…because the prevalence rate is so high of Alzheimer’s disease neuropathological change in the brains of people over the age of 80, you could have somebody walking to your clinic with Huntington’s who would have a positive amyloid scan. I mean do they have Alzheimer’s disease, possibly, do they have Alzheimer’s dementia almost certainly not, they have Huntington’s. And we see that in other studies that look at patients who have phenotypic corticobasal syndrome and a big percentage of those would have a positive amyloid scan so that has to be (talk over) (C-GDDI-Ex1)  Chronic traumatic encephalopathy, same thing? (C-GDDI4)  Not…so that’s been starting to be looked at but I do not…In the same underlying prevalence of, I mean age related prevalence of amyloid but by amyloid definition does not increase in head trauma. (C-GDDI-Ex1)  But CTE doesn’t protect them from amyloid deposition either (C-GDDI4)  No, they can get both (C-Ex1) |
| Challenge of amnestic presentations without amyloid positivity |  | so is there a subgroup of patients with… (C-GDDI1)  …amyloid negative Alzheimer’s? (C-GDDI-Ex1)  There’s tangle-predominate dementia which would not be Alzheimer’s disease. But there’s also people who are 75 and older, who have hippocampal atrophy, present amnestically, have either argyrophilic grain disease, hippocampal sclerosis and they have very long amnestic MCI prodromes and then get a mild dementing illness. And you can’t, right and they look exactly like Alzheimer’s disease and you miss it every time. Yup another one, you know, and they’re not, for us they are not unusual. (C-Ex1)  We have a…that’s a big chunk of that 15% that we didn’t diagnose correctly. (C-Ex1) |
| Combination of multiple tests likely best | In terms of limitless tests, I do think in dementia um, there may be a different suite of tests I would get depending on what clinical scenario I’m dealing with. With mild cognitive impairment or one we’re trying to predict whether someone is going to go on and develop disease in a limitless world I’d probably get you know more than one marker. I would probably want to get a marker of…some marker of amyloid which would tell me some specificity as to what label is likely to appropriate be placed onto it and some marker of brain injury which would tell me a little bit more about the timing of when we can expect to see symptoms in the future and I think that those two offer complements and I think the data, you know we have a study where we look at amyloid positive mild cognitive impairment patients and um they are at greater risk of progressing than amyloid negative mild cognitive impaired patients. But within that group if you take ones that have cortical thickness abnormalities in AD regions, um, those with more abnormalities are likely to progress in one year, those with less abnormalities are likely worse case stable for a year, or more years and so I think (talk over) (P-Ex2)  Well I think in any field you don’t want to look at just one test. (P-CP2)  That’s what P-Ex2’s been saying all along and I concur. (P-Ex1)  It’s very hard for us in guidelines to look at multiple tests because there’s no mechanism to really…unless somebody’s done a study that’s set up that way to say well are two tests better and how much better are three test better…carefully do that. (P-GDDI-Ex1)  Right (P-CP2)  We actually at least in the MCI we do have some literature saying that combinations are better predictors. (P-Ex1)  One can ask that question. You’re allowed to ask that question.(P-GDDI-Ex1)  I think it can come out again in the clinical context section to pull it together for recommendation. (P-Mod)  No one test should be relied upon to make a final prognosis and diagnosis (P-Ex1)  Exclusively… Yes (P-P1)  That’s kind of a given with diagnostic testing with psychologists, speech pathologists, (P-CP2)  It should be but it’s not always I have people who have gotten one test from a provider and been given a diagnosis off that one test. (P-Ex1) |  |
| Looking at using combination of tests | So there’s a long list there but I think we need to talk a little bit about not just amyloid imaging and isolation but the different potential availabilities especially if we’re comparing it to other, you know, who if you look at that PICO, you know it’s like what else can you compare it to. (P-Ex1) | Yeah you could say in patients with an abnormal FDG PET scan, what was utility of amyloid PET?... In patients with abnormal FDG-PET, what is the utility large term of the amyloid PET scan. (talk over) abnormal FDG-PET would capture both Lewy body and Alzheimer’s and so if the…they had abnormal FDG-PET, normal amyloid then it’s more likely to be Lewy body dementia. (C-GDDI4)  We could look at that (C-Mod)  Did I get the reasoning right? (C-GDDI4)  Well you could… so 60% of Lewy body patients will have an abnormal amyloid PET… but the FDG-PET characeristics could distinguish AD from DLB. (C-Ex1)  Do the…just a question for you guys. So is there other studies that look at or are we likely to find evidence that look at uh amyloid PET along with other things say amyloid PET and CSF with CSF alone or amyloid PET with clinical (C-Mod)  There’s one study in neurology that addresses that issue pertaining to CSF biomarkers that show that having both does not increase your sensitivity and having one or the other they’re both…they’re not significantly different from each other. (C-GDDI-Ex1)  And there’s amyloid PET versus FDG in the context of AD versus FTD but the outcome is the clinical diagnosis. (C-Ex1)  So…yeah, so I mean I think that we should just make sure that standard evaluation means uh amyloid PET added with other evaluation, compared to other evaluation (C-Mod) |
| Amyloid imaging will be more important if there is amyloid-specific treatment | If we have a amyloid specific treatment then it may be more important um to get that thing right (P-Ex2)  Iif there are drugs, you know there are various drugs in the pipeline, one that will be completing it’s um, another phase three trial (inaudible possibly drug name) in the very near future in mild Alzheimer’s disease as well as in mild cognitive impairment that is very targeted towards a beta and so if that is a positive study um, then you know we will be using amyloid imaging quite a bit in people with very mild symptoms and those studies seem to be…you know there’s also…we’re beginning a phase 3 study with another opinion based drug that’s directed at amyloid developed by BioGen and it seems like the models for those studies are that they are much more effective is you get amyloid imaging to define the group that you’re studying to increase diagnostic accuracy. So if those studies are designed in that manner then that, then amyloid imaging will inevitably be part of the criteria in clinical practice to use it. (P-Ex2)  And the second question I have is how likely in your opinion and you know we’ve been watching studies treating targeting amyloid for probably a decade and a half now, how likely do you think it is we will have an FDA approved treatment that targets amyloid that works? (P-Ex1)  I would say medium likelihood. I actually think that we’ve been operating, you can take all the studies we’ve been doing in Alzheimer’s disease pre like 2012 where we didn’t have…we didn’t include amyloid markers in inclusion criteria (P-Ex2)  I think that however, if an amyloid targeted treatment proves itself effective at any stage. It could be pre…pre MCI, it could be MCI, it could be Alzheimer’s, then amyloid imaging will actually become very important for clinical practice. (P-Ex1) | But you can imagine if we had a treatment for Alzheimer’s disease or could prevent Alzheimer’s disease we’d be doing one another treatment trial, the A4 study is doing and that’s enrolling asymptomatic people over 65 because they are a higher risk group but that age group is going to drop in a subsequent trials, enrolling them into a research study where the first step in the study is they get an amyloid PET to see if they have pathology in the brain. These are people who are otherwise cognitively normal. They score normal on screening tests, they walk around without symptoms, we tested them for amyloid and if they are amyloid positive then they can be enrolled in a treatment trial using an anti amyloid agent. So they…that theory there being that that’s the highest risk group for people that over the next 5, 10, 15 years might convert to develop symptomatic dementia. (C-GDDI-Ex1) |
| Amyloid is important for clinical trials | Can I have one more thing to add to the why you’d want to do it is if you…if there was a clinical trial that you could get into that was based on having amyloid pathology it would help you identify as being a candidate for trails and that’s certainly a benefit potentially. (P-GDDI-Ex1)  Yeah that is a common reason why our patients want to get additional biomarkers as they have interest in trials. I’ll just…as a caveat to that is that many trials now are requiring amyloid imaging for enrollment in the study. (P-Ex2)  and argue that 20% of all of the Alzheimer’s studies…of all the participants in Alzheimer’s studies at least don’t even have the disease that we’re trying to treat which creates noise in a very difficult disease to see if…to measure outcomes because of the fact that it moves so slowly and we’re looking at drugs that modify the course of the disease and so you’ve got a disease that’s moving slowly that you have to follow people at a minimum 18 months to see if there’s a difference and then you add in the noise of another 20%...I would argue that our prior studies have not been designed…so some of it could be drug, but also there are major sort of design issues. (P-Ex2)  And I would agree with that (P-Ex1) | [Quote above partly relevant] |
| Implications of negative amyloid scans | **Clinical implications of negative amyloid scan in MCI**  If we were to actually know the amyloid pathology presence or absence, you could chart out a prognosis, it would be different between those two groups. Amyloid positive or amyloid negative among MCI patients. (P-Mod)  We have a study where we look at amyloid positive mild cognitive impairment patients and um they are at greater risk of progressing than amyloid negative mild cognitive impaired patients. (P-Ex2)  the problem is when you go into your doctor with those symptoms and you have an amyloid negative scan I can tell you have a…or an amyloid positive scan I can say you have a 60% chance that you’re going to develop Alzheimer’s or you’re going to develop dementia which I think will like be due to Alzheimer’s disease. If you have an amyloid negative scan you still have a 25% chance of developing dementia due to something else. (P-Ex2)  **Implications of negative amyloid scan in dementia**  If you have a dementia and you have a negative scan it doesn’t mean you don’t have a dementia it just means it’s probably something other than Alzheimer’s. (P-Ex1)  In trials when we’ve looked at patients with Alzheimer’s disease, with mild Alzheimer’s disease, those we are amyloid negative tend to have a much slower rate of progressing. Now maybe they’ve been misdiagnosed… (P-Ex2)  Wait, yeah I was going to say amyloid negative Alzheimer’s disease? (P-Ex1)  Yeah and so maybe they’ve been misdiagnosed, um, and so one could argue that there is still some prognostic value within (P-Ex2) | **Negative scans are always helpful**  So the positive predictive value and negative predictive value is what we’re trying… (C-GDDI-Ex1)  I hadn’t looked at it that way or thought about it that way… that’s very important (C-Method)  If your father though had sleep apnea and he was on a sleep medication and he was having progressive memory problems and your real question was does he have Alzheimer’s disease causing this, a negative scan would be very helpful. (C-GDDI-Ex1)  Negative scans are always helpful (C-Ex1)  A positive scan in that age group does not remove the clinician’s responsibility to investigate absolutely every alternative. You can’t hang your hat on that and say Oh this is classic AD. (C-GDDI-Ex1)  Why do you say a negative scan is always helpful? (C-Staff)  Because it is so reliably predicts Alzheimer’s pathology and you be pretty certain that person doesn’t have Alzheimer’s. (C-Ex1)  If we took C-Method’s dad and scanned him and it was negative… (C-Staff)  Even if he’s not symptomatic he’s very unlikely to develop Alzheimer’s dementia, that’s what you’d conclude. (C-GDDI-Ex1)  In the next ten years he’s unlikely… (C-Ex1)  **Negative scans prompt reconsideration of diagnosis**  So I’ve enrolled people who have dementia, who I told them have Alzheimer’s disease dementia by all clinical criteria. Their imaging looks like it, everything else and their amyloid PET is negative and that patients population that’s a hugely important finding because now I have to really go back and unearth any possible disease to follow up on that. Do they likely have DLB, probably, or could they have…so I’ve seen people for polysomnography that had no symptoms before to investigate for it. You know just thinking about other causes that…I mean I’ve already done my pre work up before the amyloid scan, I did what’s indicated for them and I’ve appropriate screened for symptoms but now I’m back at square one because that negative scan is very important in that population. The positive scan would not have had any incremental value for me. (C-GDDI-Ex1) |
| No information on re-testing |  | And do you have to talk people out of getting like a second scan or a follow up scan after a period of time to say look the likelihood is pretty low (C-staff)  We haven’t come across that. We haven’t been doing it long enough to (C-GDDI-Ex1) |

Table 5. Themes and Exemplary Quotes Relating to Population for Guideline (“P” in PICOT)

| Theme | Gp 1 (Exper, WITH PATIENTS) | Gp 2 (Control, PHYSICIANS ONLY) |
| --- | --- | --- |
| Testing people who are asymptomatic or presymptomatic/at-risk | **What does “at risk” mean**  So can we then take down the at risk question also? Would that be justifying by age alone or a familial Alzheimer’s or family history of Alzheimer’s at a young age or dementia at a young age. What else would be considered at risk? (P-Mod)  Well the at risk would be age. Age is the biggest risk, second risk group would be people who have…I would argue would be people who have first degree relatives with early onset of dementia or who have a known genetic risk. (P-Ex1)  So, um the other risks, um, so we’ve got age, we’ve got genetics, we’ve got family history, you know there are, one of the big risks is cognitive symptoms right? (P-Ex2)  Although to me that takes us out of pre-clinical at least into prodromal potentially (P-Ex1)  There has been literature that traumatic brain injury is a risk factor for Alzheimer’s disease and so I know that that…and there are other risk factors are you know vascular disease (P-Ex2)  Diabetes (P-Adv)  All the vascular risk factors, essentially, metabolic syndrome… (P-Ex2)  Sleep apnea is a risk for cognitive impairment (P-Ex2)  So another…I think another, I mean some of those are risks along the spectrum like we were talking about with cog, so you know depression actually, late life depression in particular, but depression is a risk factor, um, for the disease, um, you know other sort of late life psychiatric symptoms are almost certainly, you know sort of behavioral symptoms are at risk for the disease because sometimes that’s how people manifest. I mean you can get very granular with this like weight loss late in life is associated with risk of Alzheimer’s disease, delirium is associated with risk of Alzheimer’s. (P-Ex2)  Then the thing I’m wondering about population just so I don’t lose my thought, is does gender need to be here? Because of the significantly greater increase for women? (P-Adv)  It’s a typical significance so what 60/40 split roughly, but you know 40% is still pretty large amount. I don’t know if I’d get too much there. (P-Ex1)  think it’s another risk factor I mean if we’re listing vascular disease it’s you know there’s plenty of people without vascular risk factors that get Alzheimer’s as well and so you know one could imagine um a confluence of risks that would maybe obviate into get an amyloid scan in certain people or not, um you know, certainly like for example when we were talking about this in terms of like genetic risks. If you’re APOE 4 positive and you’re 75 with cognitive symptoms you’re risk of being amyloid positive is probably on like the 90%, 95% and our accuracy of the diagnosis is much higher clinically in that context and so risk can certainly influence you know the utility of the test for answer the questions. I so think it’s a reasonable… (P-Ex2)  In this case in patients at risk for Alzheimer’s disease… We talked about all those risk factors, we have lots of risks. (P-Ex1)  The first would be relative with early dementia, the known…other known (inaudible) factors (P-Mod)  I would actually get rid the “without symptoms” and make it without cognitive impairment (P-Ex2)  That will also capture that subjective cognitive complaint category if you are definitely at risk of (P-Ex1)  **May end up saying don’t test at risk/preclinical people**  I think you’ve got the asymptomatic folks who might be at high risk for Alzheimer’s disease that have to be considered at least to…and even if it’s being considered to say don’t do it. (P-Ex1)  And maybe that’s what we will say is in the end if there is ongoing research into this question but until those results are available we have to recommend against its use in regular clinical practice. (P-Ex1)  **Some preclinical people are going to ask for test**  So we still haven’t answered the question does this need to have you know preclinical and dementia level patients. (P-GDDI-Ex1)  I agree with P-Ex2 that the most important question might be in that mild cognitive impairment group, I mean that might be the most important answer, but I think the other two are valid questions that people are going to ask and come across if we can get guidance for this. (P-Ex1)  I heard from our non neurologist colleagues here that people want to know if they’re at risk for getting this disease. Is that accurate even if they don’t have symptoms yet, people want to know or at least have that option available? (P-Ex1)  I would think so (P-P1)  I think some people would but there’s so many people who are in denial already (P-CP1)  I think it’s the option, I think it’s to have the option available (P-Adv)  So I think we do have to make some statement about asymptomatic (P-Ex1)  Having that certainty or having that knowledge being able to plan is important. (P-Mod)  I would agree with that. I think there are some people that might find that depressing and have that…I mean I wouldn’t say necessarily everybody should do that, I mean, be prepared for that, but I think in ordering your life your career paths, your family things and that having some idea of what might happen. (P-P1)  **When available people will do test if guideline doesn’t comment**  I think if you remain silent on this and the others go through and amyloid imaging becomes available, people are going to be doing pre-clinical amyloid imaging unless we make some comment. (P-Ex1)  **Patients with a family history of dementia may want test**  - Let me ask about a clinical scenario. Maybe there’s a patient with a family history of early dementia, we don’t know really what it was and the patient’s 40 or 35 and they want to know if they are at risk of Alzheimer’s. Is that appropriate to…would it ever be appropriate to look for the amyloid pathology in a 40 yr old, 45 yr old with a strong family history that’s really uncharacterized. (P-Mod)  - Yeah I mean you know I think if they…I mean I think they are already at…so I guess there’s the question, Yes I could imaging if there was an evidence base about that but I don’t know how right now we know enough to say (talk over) (P-Ex2)  - I can tell you what I counsel people in my clinic if they ask that and a lot of caregivers or family members do. And I say I do not recommend doing it clinically, I recommend it you are interested in pursuing this to get in a good, you know, FDA approved research trial to look at that question if you’re going to go down that route because I do not feel that we have enough to offer you clinically to make this, you know something…I think the harm outweighs the benefits unless you are doing it as part of research. That’s what I tell my families of patients that. (P-Ex1)  - Yeah I guess since I don’t know outside of the…I mean I just…I agree, I mean in terms of how we would address that other than to say, you know like you said, that there’s insufficient evidence to show there’s any value in you know obtaining this data and I don’t…I think that’s almost like a…that’s sort of common knowledge. (P-Ex2)  - No that’s what I’m going to tell you, it is NOT common knowledge. That’s the problem P-Ex2 it’s not. Among you and I it is, several neurologists it is, but in general practice world it is not common knowledge and we need that guideline to say something even if it’s to say we don’t know because sometimes people don’t know what we don’t know. (P-Ex1)  - Yeah so I mean I think um, we certainly can pose the question and we would certainly be able to easily say there’s not enough evidence to suggest that there’s any um, clinical impact and that we don’t know the risks and harms associated with pre-clinical diagnosis. (P-Ex2)  **Testing people who are asymptomatic should be done only on a research basis**  You know I think for creating, I mean just from a utility standpoint, I think creating guidelines for pre-clinical Alzheimer’s disease, I mean unless we are trying to make a research recommendation strikes me as a thorny path that we don’t necessarily want to move down with regard to amyloid imaging. Um, and I just…and I don’t know what even literature base…I mean we can answer a question like how well does it predict eventual development of Alzheimer’s disease of clinical symptoms I suppose, but I’m not sure from a guideline…in terms of like bring this into clinical practice, A) there’s the clinical base to completely address it, and B) I’m not sure there’s the clinical need right now in the context of what’s available to intervene (P-Ex2)  I agree (P-CP2)  But I think that is a guideline, you’re guideline is this is not ready for regular clinical practice, it should be research…available. It needs more research. That would be for the…what you’re saying for the pre-clinical. I think not…I think the (talk over) is a problem (P-Ex1)  My point is that we just don’t…so there is too much uncertainty about what the actual… yeah, how to interpret what that means (P-Ex2)  I think in this day and age pre-clinical, us screening for pre-clinical disease is beyond what we do clinically, it’s a research…it’s only been done in that kind of research. (P-Ex2)  Well that could be the question we ask is, is there enough, Ok, evidence for making a determination whether it should be only research versus available in clinical practice at this point. I mean they can come up with insufficient evidence and call for research. (P-Ex1)  **More research is needed to understand impact of results**  And there is actually an emerging literature, but just studies being done now actually some of it by a colleague of mine looking at the impact of disclosure on cognitively normal older adults and how that impacts them psychologically, how that impacts them with regard to planning, who wants to know, why do they want to know and so it just…but I know it’s such a (inaudible) issue that’s it’s just um…  **Test will predict future cognitive symptoms but value of that unclear**  W e can say it [a positive amyloid PET] improves the likelihood that they’re going on and development cognitive symptoms in the future. We can say that with certainty, the question is what the value is of that. And that I think is to me the more important question and I don’t think we have an evidence base to say that. (P-Ex2)  So really there’s two questions there, it’s you know…and one is sort of outcomes and the other is validity. You know 1) does it provide an accurate enough prognosis and 2) does knowing that prognosis improve or diminish the quality of life for the individual. (P-Ex1)  **Implications of age on presymptomatic testing**  And I think the other thing that is a factor is early onset versus late onset when we start talking about prodromal. Even for preclinical I mean if you’ve got someone doing these tests who is age 30 versus age 70 and is asymptomatic I think that is a slightly different question. (P-Ex1)  It might have different clinical prediction, but if we’re talking about pathology I don’t think it’s necessarily different. (P-Ex2)  **Potential harms**  I think so especially if you’re going to frame this in the context of pre-clinical disease. Just like with any kind of genetic…you know if these conversations about like you know Huntington’s disease and all these other things we’ve gone through and I think the harm is just as critical to predict as the…or should be emphasized as well for potential… (P-Ex2)  **Appropriate Use Criteria recommend against testing asymptomatic individuals**  Well we could include pre-symptomatic um, but I think that you know again not that we need to necessarily go by this, but you know the Society for Nuclear Medicine Appropriate Use Criteria specifically is not for pre-clinical disease um and so it just comes down to how we define our terms, but pre-clinical…I think in this day and age pre-clinical, us screening for pre-clinical disease is beyond what we do clinically, it’s a research…it’s only been done in that kind of research.  **A positive preclinical scan doesn’t change a physician’s recommendations too much**  pre-clinical use the amyloid scan tells you your risk but it doesn’t actually, other than go get in a clinical trial, it doesn’t tell you to do anything different and Oh plan ahead it doesn’t tell you to do anything different that I would tell to someone who had a negative scan which is you should all have your advance planning and long term care and you should be exercising, keeping kind of actively socially engaged so it may help to people who are at higher risk for doing it earlier to get on the ball so that would be the benefit there. (P-Ex1) | **What does “at risk” mean**  But everybody is at risk, right? … So we won’t specify them, we’ll just go by the literature. A question is going to be asymptomatic, would you like to further define asymptomatic? In what way? Clinically asymptomatic, patients as asymptomatic, families as asymptomatic, I say I’m asymptomatic and everybody thinks I’m crazy? (C-Mod)  No cognitive complaints and are neuropsychologically normal. (C-Ex1)  And at the end of the day it may be a judgment call when we look at the studies that we pull saying do we really think that they’re asymptomatic or not or we may, you know, sort of ask if there are criteria of what they call asymptomatic and then look at the literature from that too. (C-Mod)  But I think the definition of asymptomatic is going…I mean if they’re in your [dementia] clinic they’re probably not asymptomatic. If they’re in a PCP office, that’s different, I mean our guidelines are applied broadly. Our…the guidelines are going to apply broadly to all to all physicians care, you know not providers. Not just the specialists. (C-GDDI3)  Is this question really just trying to get a the rescreening people at a population level. Because if that’s what you’re trying to get, you know, then I think we just leave it as asymptomatic so everything else is going to fall under subjective. (C-GDDI1)  **May end up saying don’t test at risk/preclinical people**  Yeah at risk with family history or over age…I don’t think…we’re not going to make a recommendation testing that population, right? (C-GDDI-Ex1)  **Implications of age on presymptomatic testing**  Would you like any sort of age criteria for the asymptomatic? (C-Mod)  Probably… (C-GDDI-Ex1)  **Importance of knowing diagnostic (screening) value in general population if asx individuals might have test**  If we ran into an asymptomatic study that would be helpful. Now if we don’t run into an asymptomatic study what we want to be able to do in and this gets to the next question, is that we want to make some inference to what the predictive value of the test would be in a lower prevalence population and so do that I think we need to ask a 4th question which has to do specifically with the specificity of the test. So it’s not diagnostic accuracy… this is a screening question… and it’s for patients at risk but this is mostly going to be asymptomatic patients. Um…so and it’s screening so doesn’t…if you do amyloid PET and then this is if you…put it in parenthesis because it…assuming you didn’t do amyloid PET but you wouldn’t find anybody. How often…so the outcome is how often do you identify amyloid, right. So you were giving us numbers if you were 30 yrs old how often…that’s, that’s critical information for us I think to fill in this populations. I think I’m anticipating based on what I heard, we’re not going to find a whole lot of data in this population and we want to be able to tell people, you shouldn’t be doing this, you know, willy nilly on asymptomatic people because this is what’s going to happen, you’re going to find the amyloid in this percentage and it’s not going to mean anything. So this is…diagnostic accuracy question but it only has to do with specificity it’s a specificity question and the search strategy I think is going to be different so I think this should be a separate question… We’re only….we won’t be able to build a 2x2 table we’ll just be able to tell what proportion of patients that we know doesn’t have ADD because they are normal, have amyloid. So we’ll just have a numerator and denominator. (C-Method)  Oh yeah, you will find a lot of this (C-Ex1)  Yeah and that will be useful because we’re going to want to fill in the gaps I think on these patients uh and it’s going to really have strong implications for (C-Method)  Informing people why not to get this (C-Ex1)  **Testing asymptomatic individuals may eventually have implications for treatment (research now, clinical eventually)**  Or take an asymptomatic group and see if they could have amyloid pathology underneath in their brain and therefore sort of decide treatments based on that. (C-Mod)  There is…so no because there’s no treatments available right now, so nothing clinically so the one is sort of decidedly research, scanning asymptomatic patients would be a lot like doing colonoscopies on people if you have nothing, Oh you’ve got a polyp in 10 years you’ll probably have a cancer. With the analogy to that we do it for a different reason because we can treat it. So that’s very much in the research trial scanning asymptomatic people but the primary clinical use and thinking is trying to figure out the cause of people’s complaints usually or the cause of what we as clinicians determine to be cognitive impairment. (C-GDDI-Ex1)  **Appropriate Use Criteria recommend against testing asymptomatic individuals**  The areas where they said it would be inappropriate would be one um, in people who are asymptomatic but have a family history of Alzheimer’s disease in people who um, don’t have subjective cognitive impairment but don’t have any objective testing findings (C-Ex1)  **Positive amyloid imaging may be the first indication that something is wrong**  Yeah so the way they’ve done it is they’ve done it based on the NIA Alzheimer’s Association Criteria for pre-symptomatic so you have different stages. Um so if based on biomarkers so uh stage zero would be people who are amyloid negative and are negative what’s called neuro-degeneration which would be hippocampal atrophy or FDG PET abnormality or elevated Tau on the CSF. And then you have stage one which is amyloid but neuro-degeneration negative. Stage two amyloid positive neuro-degeneration positive and stage three is the nebulous stage of amyloid positive, neuro-degeneration positive with some slight changes in the neuro-psychologic testing not quite meeting MCI criteria. And as you go through those stages the, you have a higher and higher risk of developing cognitive impairment but certainly if you’re amyloid positive or if you just take people who are amyloid positive versus amyloid negative, they have a significantly higher risk. If you take people who are stage 1, their risk of developing cognitive impairment at 5 years it’s about 11% and then it will go up as you go through a half dozen other biomarkers. (C-Ex1)  **Meaning of a positive amyloid scan if cognitively normal**  But when it comes to practical applications there will be people that are cognitively normal that would get imaging and have a positive test and they are going to ask well does that mean… Is that a false positive? Or is that pre-symptomatic? (C-GDDI4)  **Important to prepare (eg buy insurance) before testing**  you may have to have people have a pre-symptomatic testing protocol that includes many steps before they step foot into the clinic and that is get everything lined up. Take care of your life insurance, long term care insurance, make sure your finances are in order, anything that can be messed up by this test result either way you need to get taken care of. (C-GDDI4)  Yeah because the genetic non discrimination act is only applicable to genetic testing, it does…and even that is not uniformly followed. It is supposed to be but it’s not. (C-Mod)  It hasn’t been tested (C-GDDI4)  It does not apply to this kind of testing (C-Mod) |
| Testing people with subjective cognitive complaints | [Group 1 included this in at-risk/presymptomatic] | **Defining subjective cognitive complaint population**  - So they are coming into your office complaining of something but they have no… normal exam, normal neuropsych, (C-GDDI2)  - They’re maintaining their job as an AAN methodologist, they are contribution to new guidelines and their wife says that No this is…sure he forgets an occasional name but no different now than three years ago or four years ago. No different than the other people that hang out with. (C-GDDI-Ex1)  - Shouldn’t subjective be kind of defined though as like after routine evaluation? (C-GDDI1)  - Yeah, I’d say neuropsychological normal (C-Ex1)  - Without impairment. So subjective cognitive decline. (C-Mod)  They’re the subjective group so they are not asymptomatic they are symptomatic but didn’t meet a diagnostic…didn’t meet a diagnostic criteria for MCI or AD. (C-GDDI-Ex1)  **Excluded from testing by Appropriate Use Criteria**  That’s really important so in terms of the subjective group, um, and that was why the appropriate use criteria excluded them because it’s a mixed bag and it looks like people who have subjective cognitive impairment are at increased risk of developing MCI and dementia later compared to people who don’t but it’s not a certainty and so um, the appropriate use criteria excluded them because of that. (C-Ex1)  But the appropriate use criteria is all based on the premise that we don’t have a treatment for AD where as our guideline doesn’t have to be based on that premise. (C-GDDI-Ex1) |
| Testing people with mild cognitive impairment (MCI) | Then there’s the prodromal, these MCI folks and I think we need to make a statement about that. They have some sort of behavioral and cognitive change, by definition but it’s mild. They’re not yet crossed the borderland into dementia yet. (P-Ex1)  **Helpful in MCI because more uncertainty of diagnosis**  So the more mild you are the more uncertain you are about the diagnosis. (P-Ex2)  **Establishing pathologic cause of MCI**  There are criteria um, you know through the Alzheimer’s work group for mild cognitive impairment with regard to its sort of clinical syndrome and then prodromal Alzheimer’s disease is based on the presence of biomarkers including both amyloid and neuro degeneration that make it more likely so what I guess you could say is, you know people in the mild…patients with mild cognitive impairment, um, what it’s likelihood of predicting change because it’s not really…at that point when they come in they’re not prodromal AD, they’re not prodromal AD until they get an amyloid scan, um when we think that they have, you know. So does that make sense? (P-Ex2)  Well the patients coming with memory or cognitive trouble and they still function you call them MCI whatever terminology you use, one of the things you want to know is do you think this is Alzheimer’s or something else. That’s a clinical question. Whether or not that’s useful or not that’s a different discussion. (P-GDDI-Ex1)  **MCI is common clinical presentation**  I think the clinical scenario that’s most relative is the mild cognitive impairment where um that’s where we’re all…as clinicians… To me this is like the question that I think is of the most disseminable um value (P-Ex2)  Again I agree with P-Ex2 that the most important question might be in that mild cognitive impairment group, I mean that might be the most important answer (P-Ex1) | Then you can ask the question, does amyloid PET accurately distinguish patients that meet clinical criteria for MCI related to AD versus you know MCI related to something else, some other disease pathology. Or does amyloid PET accurately identify patients with MCI that are destined to develop AD and progress. (C-Method)  **Prognosis in MCI**  So in terms of MCI, you’re diagnosed, your accuracy is going to be…how many of those MCIs actually have AD… That sort of again takes on a prognostic flavor, right? (C-Mod)  it makes you also wonder um you know you could have MCI for a long period of time, is it also useful in predicting the conversion to dementia. So if you are MCI amyloid positive will you convert in two year or is it if you’re MCI amyloid negative 10 years. You know what I mean? So is it useful for prognosis. (C-Ex1)  It could also be if your MCI had amyloid negative what is your chance of reverting back to normal? Or not progressing either way. (C-GDDI4)  **Use whatever MCI definition is in study**  MCI? Do we want criteria or shall we go by the criteria that we use in studies in the pass those out as we go? (C-Mod)  Yes (C-Method)  I think that’s the better way to go because there’s so much variability (C-GDDI-Ex1)  MCI as defined (C-Method) |
| Using test in populations with dementia | **Will tell about pathology more than progression in these patients**  symptomatic stage of dementia where we have a…where someone already…our level of certainty that they have something significantly going on, um, I think it’s more about what rather than timing. (P-Ex2)  **Usefulness when atypical presentations**  Um, do we want to say anything atypical characteristics of cognitive disorder maybe make it more valuable to know? (P-Mod)  So I think um, one thing that captures that a little bit is uncertainty in general so you know I think more broadly. So I mean I think to me that is again, depending on how you’re framing the question and what the goal is in terms of getting the diagnosis right, atypicality versus atypicality of the symptoms of the disease alter the value of potentially amyloid imaging. So um, and if you broadly say uncertainty then I would…I think atypicality as well as mildness. So the more mild you are the more uncertain you are about the diagnosis. There’s a lot of great data that neurologists do better at predicting autopsy, um, at the last visit they saw the patient than at the first that they saw the patient and (talk over) you know the patient better but you’ve also…they are not at a later stage where’s it’s more obvious what’s going on. (P-Ex2)  But if I have a cloudy picture it’s just a…it could be in part A, part B, part C it could help as a tie breaker to determine which type which may affect your treatments that we just mentioned which in terms of at least risk benefit. (P-Ex1)  [Use of amyloid for] distinguishing dementia is uncommon but you know it comes across a fair amount of we’ve got a messy picture. What’s our best bang for buck to try and figure out which dementia we’re dealing with. (P-Ex1)  **Not very useful for typical presentations**  If you have a dementia and we are having trouble…and you have the classic picture and demographics, I already know what the amyloid scan is going to show me, it adds very little. (P-Ex1)  Who have symptomatic dementia, what rule do you use that would or would you use if you had sort of resources for beta amyloid imaging? (P-Method)  Basically if I could make the diagnosis based off your clinical picture, none. (P-Ex1)  **Useful in young onset dementia**  We know early on some dementias while rarer are often times even more heartbreaking for families. It’s always heartbreaking but when you have, these people have young children, these people are still in the work place and often times might be the primary breadwinner for a family. So if they’re starting to have problems, knowing and being able to prepare for it and being able to get them resources and support is extremely important… It would be helpful to determine whether they have Alzheimer’s developing or not in the early onset crowd. (P-Ex1)  I mean…so I mean I think it’s…first of all a lot of young onset, meaning young age of onset cases are a bit more atypical with AD and Alzheimer’s disease in general so there can be clinically more overlap, um, knowing amyloid status might push you more towards wanting to treat with Alzheimer’s disease drugs, there’s some that at least that acetylcholine inhibitors, one of the main drugs like Aricept that we use in Alzheimer’s actually has negative effects in frontal temporal dementia causing behavioral um symptoms. Um, where I think that is a population in which its particularly useful for in the sense that it’s a population where you’re also…you’re, the things you’re thinking about are very broad if you’re also thinking about Para neoplastic things, other things, and so um, it may reduce the um, the number of tests that we need to get (P-Ex2)  In my young cases having a more definitive diagnosis is um, is helpful. You just…there’s much more of this tier that you’re missing something else um, that could potentially be treatable. (P-Ex2)  I would agree (P-Adv)  I think we also have to talk about early onset versus late onset because I think that changes the value or at least changes what to highlight in making the determination. (P-Ex1) | **Usefulness in atypical presentations**  What features you know what atypical features would lead one to want to do amyloid imaging. Is there identification of sort of the best. I mean so you know identification of the best or most…the patient population in which this would be the most useful. (C-GDDI-Ex2)  So the unusual disease course, so not gradual and then non-amnestic, so people with executive behavioral language presentations (talk over) with the possibility of having Alzheimer’s disease. (C-Ex1)  Can I ask a question about this not gradually progressive? Because that…immediately in my mind you know I mean raises the possibility that it’s everything else going on so is it better sort of look for all those things you know like paraneoplastic antibodies, look at your MRI picture, do a CSF analysis, I mean, we all do all of that right? (C-Mod)  Well in certain cases so if your like a truly rapidly progressive dementia then you’re not going to use an amyloid scan to knock through the work up, but um you could still have…you know there’s people with a sudden onset and are stable so you’re thinking vascular or sudden onset and more gradually so maybe there’s vascular and Alzheimer’s or (C-Ex1)  Gradual that turns rapid. So that’s a common one (C-GDDI-Ex1)  And that’s the most common one. It’s a little bit and then you’re going Oh my gosh they’re really bad. The other stuff is negative. So if you’re going to do the other stuff (talk over) (C-GDDI-Ex2)  Just like we were talking about how C-Method’s father had a negative PET scan, it would be helpful over 90…so it’s not the expectation, you know in younger people where their rate of positive PET…PET amyloid is 10%, a positive scan in somebody who has no family history and are 55 and they have memory problems it would be…it’s very compelling and very helpful. Because that’s someone who you’d be otherwise investigating pretty much through every other domain. You would send perioplastic markers probably (talk over) (C-GDDI-Ex1)  *Appropriate Use Criteria recommend use in atypical presentations*  And just to give some background, in 2013 Keith Johnson wrote a paper called basically Appropriate Use Criteria for Amyloid PET and uh since Medicare wasn’t covering it but it was an FDA approved test they took experts and came up with certain scenarios where it would be appropriate and inappropriate to ordering amyloid PET tests. And so what they said is the first thing is you had to have objective cognitive impairment. It could be mild cognitive impairment or dementia and after an evaluation by a dementia specialist which would be (inaudible) say neuro psychologic testing, MRI scan there was significant…If there was significant uncertainty to that diagnosis but they met possible AD criteria, um, then they could be a candidate for an amyloid PET scan or if they had a prolonged say mild cognitive impairment phase, they weren’t progressive but clearly had impairments testing that would be another group that could considered for an amyloid PET. Um, and then in people with dementia under age 65, um that would be the third group where it wasn’t clearly AD after a routine evaluation by a dementia specialist (C-Ex1)  **Incremental benefit probably not very high in typical presentations**  So I think gets in an important distinction when we’re looking at diagnostic accuracy. So regardless of the reference standard you can look at diagnostic accuracy just from the perspective of the test itself it would probably be pretty good for patients with typical Alzheimer’s dementia defined clinically. Um, and it’s worthwhile asking that question because if it wasn’t good then we wouldn’t do the test. But then you’re going to want to do the comparative diagnostic accuracy, is it more accurate combined with when you add it with the you know clinicians evaluation to the clinicians evaluation alone. Um and there I think when you look at patients with typical dementia, typical that…that…I mean we’ll have to see but I suspect that the incremental value is pretty darn low, but in the other populations it’s much higher. (C-Method) |
| Importance of considering how age impacts population being studied | I’ve brought it up multiple times and I’ve heard it confirmed that we should be looking at the way age affects these results, early onset versus late onset. How do we phrase that so that we’re giving that guidance for the guideline. (P-Ex1)  You’ll follow up if there’s data, it’s present… If it’s present in the data it will follow, in my thinking… We don’t have to put that into the PICO question (P-Method)  I mean theoretically it does impact again in mild cognitive impairment your age is also a pretty big predictor or the likelihood that you’re mild cognitive impairment is due to Alzheimer’s. (P-Ex2)  Yeah it’s like considering special populations, so on guidelines you think about special populations and the ones we always think about are gender and age and then we identify whether there are other special populations we care about for that it could be early versus late onset. (MJA)  I was going to ask a question about age. Is there a lower bound where you think it’s really helpful because it’s just possible that they would have Alzheimer’s developed (P-Mod)  So again I think it depends on if we’re talking about, um, sort of pre-clinical symptoms, if you wanted us to screen, you know I mean (P-Ex2)  Would you do it for 30 yr old, I’m saying if you have a 30 yr old with cognitive disorders you wouldn’t even order the amyloid imaging because there’s just no way, like (P-Mod)  There are more mutation carriers of people who like maybe didn’t have um enough family history to know about their autosomal dominant brain or a (inaudible) mutation where people can develop the disease in their 20s and 40s (P-Ex2)  …That being, you know, so um, that being said I mean again you’re dealing with a 30 yr old with cognitive symptoms is just tremendously lower than you will in a 60 yrs old or 74 (talk over) (P-Ex2)  So is there a lower range, range? Um, that you would (talk over) Also is there an upper age? (P-GDDI-Ex1)  Um, so for amyloid imaging you know it’s a reasonable question, uh the lower age I think I agree that you know in the absence…if you’ve got someone who’s got a clinical pattern that looks like Alzheimer’s who’s very young and you have inadequate family history to make a determination I think the amyloid imaging would be reasonable. (P-Ex1)  I think it’s hard there are some, there’s some percentage of people who get into their 90s and the oldest old that don’t have a lot of amyloid in their brain and so given that there’s something to discriminate people who have amyloid versus not, I think it’s hard to draw…again it’s just like the 25, you know talking about like the question we can answer (talk over) (P-Ex2)  I would concur with putting No upper limit but again just like I was talking about that sweet spot for diagnostic and you know I think with age there’s a sweet spot, you know, the younger you get it’s going to be much less likely to be useful. I think the older you get it’s going to be much more and there’s a sweet spot really around that, I would say that 45 to 65 range is really the sweet spot where it becomes you know pretty useful. (P-Ex1)  Well I’m just, I was struggling a little bit with not having an upper age limit and this is too much of my history in working with long term care is if you’ve got the 90 yr old person who maybe is now starting to show symptoms and looking at some of the things that we wrote over here in terms of diet would be beneficial to have it as an option. Are those benefits going to apply to somebody that’s already in a modified care situation where the symptomatic treatments are an interesting conversation at best as to whether or not there’s going to be any impact and then if you’re thinking about cost. So that’s just my comment. (P-Adv)  So P-Adv if you’re going to throw that in I do not like ageism, I would not base that off of age, I would base that off…at least not chronologic age. If you will I would almost base it more off biologic age. If someone has multiple medical (inaudible) their life expectancy based off of that is like short, I don’t care if you’re 95 or 75 with that, you know. Because I have seen 95 yr olds who are still playing golf and I’ve seen 75 yr olds who are in a nursing home and not from dementia. (P-Ex1)  It strikes me that again sort of getting back to the spirit of asking a question that you know, that we’re asking a question about where is it legitimate to think about amyloid and the presence of amyloid or not and then what we could answer is what’s the value or what…does it…is it predictive in a 90 yr old versus a 70 yr old and I think for reasons that…I mean I think it may not be helpful, ma not discriminate a 90 yr old in terms of their likelihood of progression versus not. (P-Ex2) | Over the age of 65 you said? (C-GDDI2)  That’s just because they are trying to narrow the risk group and so at 65 I think they say that’s…we’ve got to enroll between 14 and 15 people to find that one person who’s walking around with a positive PET scan which we think means they have amyloid underneath probably Alzheimer’s disease pathology. (C-GDDI-Ex1)  So is sounds like….is there a profile of person whether it’s age or work history or whatever where the testing is not appropriate or would not be useful like if they are over 80, if their history is DLB, is…are there (C-GDDI3)  Yeah, exactly. Yeah I would agree that age group where it’s much less useful (C-Ex1)  Oh that’s interesting. So like my 92 year old father who’s perfectly functional… (C-Method)  40-50% chance of that being a positive amyloid test (C-GDDI-Ex1)  Right (C-GDDI3)  And so it would be very unlikely to be helpful (C-Method)  So the positive predictive value and negative predictive value is what we’re trying (talk over) (C-GDDI-Ex1)  I hadn’t looked at it that way or thought about it that way… that’s very important (C-Method)  At what age typically do amyloid levels naturally increase like say they would cross that threshold, just age related. Do we know that? (C-GDDI3)  Yeah, I mean they are continually increasing (C-Ex1)  There’s population based studies available in imaging so you get exact numbers for percentages. (C-Ex1)  But like the positive predictive value is reduced after age 80, what is the negative predictive value? (C-GDDI3)  We only have a 10 year horizon though so someone’s tested at 65 and it’s negative you can say they probably won’t develop Alzheimer’s by 75 but beyond that you really can’t say. (C-GDDI4)  Right but they can still get Alzheimer’s (C-Ex1)  And our guidelines do often include that kind of population based data and I think that would be informative for at least form the clinician standpoint to sort of have that. (C-GDDI-Ex2)  I was just going to say I have just some prevalence rates just to give everyone where we’re looking at. So at age 50 cognitively normal um about 10% will be amyloid positive, um, age 60 15%, 70 23%, 80 33% and you know 45% at 90. (C-Ex1)  So 80 I mean they have a 60% chance of being negative and not having the disease. (C-Mod)  Yeah, these are normal, not MCI (C-Ex1)  Right so cognitively normal they have a 60% chance that they will not, they will have negative scans. (C-Mod) |
| Most useful for moderate symptoms | I’m going to argue there’s a “U” shaped curve to that. It…depending…a U shaped curve for the first time you see them (P-Ex1)  Yes (P-Ex2)  Because you know if they’re too mild it can be hard but it they’re too severe all these diseases start to blend together and it would be hard. And the sweet spot is somewhere in that late MCI mild dementia state that you know you get the crisps patterns of deficits and you can really start to make a clinical statement. I agree with the longer too you know the longer you’ve know them the more likely you are to have a good gun on that. (P-Ex1) |  |
| Importance of assessing for treatable causes of dementia before amyloid testing | So in people at risk for developing Alzheimer dementia or who may already have Alzheimer dementia who have already undergone basic dementia screening… (P-GDDI-Ex1)  [Group agrees that basic screening must be done pre-amyloid, wrote it into question]  We will have to be careful in figuring out how far the guidelines, we’ll have to be careful in defining basic dementia screening. [P-Ex1] | My sense was that you would do the scan only after you look at all these things, right? Isn’t that the…isn’t that the at least the criteria right? If I did it right now, so you look at everything and then you don’t find anything, the picture is atypical, that’s when you look right? (C-Mod)  But the scan isn’t covered by insurance. How would that change if you had similar coverage if it were covered? Would you jump straight to the scan? Or would that be the temptation? (C-GDDI3)  I don’t think you would ever avoid checking a B12 and the thyroid because they could be contributors, because I’ve never seen cases where they’re the only cause. I mean that’s extraordinarily rare… like a once in a lifetime thing. But um, I see lots of cases where people have mild cognitive issues that are exacerbated by a thyroid problem. (C-Ex1)  So before you order amyloid PET, um, do you in all of these patients who clinically look like AD get TSH and B12? (C-Mod)  Yes (C-Ex1)  And some form of structural imaing (C-GDDI-Ex1)  So like a typical person if they’re mild they would get an MRI, neuropsychologic testing, a B12, a TSH, and (C-Ex1) |
| Interaction between genetic testing and amyloid imaging |  | I did have a questions about genetic testing. So is it predictive at all in comparison to like the amyloid PET like if you have the APOE4 or any of that. I mean what does that prove if anything? (C-GDDI2)  So APOE4 is a risk for Alzheimer’s and therefore for amyloid deposition but it’s not a causal…a cause of mutation but there are causal mutations and so if you have that gene then you will 100% get Alzheimer’s disease dementia if you live to be 50 or 60 or whatever age for that mutation. But I don’t…and that’s probably separate from what we’re from what we’ll cover here, but (C-GDDI-Ex1)  So if you are cognitively normal and you’re APOE4 negative, you’re much less likely to…you’re significantly less likely to be amyloid PET positive and if you’re cognitively normal and APOE4 positive but it’s not a huge gap between the two. (C-Ex1)  And from my understanding about the other genes you know is that that’s still a small subset of Alzheimer’s disease on the whole, so it’s like 10%, it’s like…total number I think is like 10% of the total amount of Alzheimer’s disease that actually have genetically…a genetic mutation that is positive. (C-Mod)  1% (C-GDDI-Ex1)  1%? (C-Mod)  It’s miniscule (inaudible) 1% and (inaudible) 2. (C-GDDI4)  And 1% so that is a positive mutation not APOE 4. And so in those patients I mean the…my understanding is that if you do amyloid PET they’re going to have a positive amyloid PET imaging but in them it doesn’t really change anything, right, I mean because of they have the genetics. (C-Mod)  And so we use those studies of those families, a large number of families with this…with these mutations to better understand the potential time for Alzheimer’s disease. So you can do amyloid imaging in a 20 yr old from that family and say is it positive, someone that you know will develop the disease at some point and so that’s been informative for some of the biomarkers, biomarker studies but it’s…I don’t think we’re at…I don’t know if we have…there may be some evidence on APOE4 rates and predictive rates and maybe we’ve used that to inform our decision making but otherwise it’s not…we don’t routinely recommend genetic testing in clinic unless they have a strong enough family history. (C-GDDI-Ex1)  And also atypical features, I think about my own patients, like with Downs Syndrome, would I do this in a patient with Downs Syndrome because they are at increased risk for Alzheimer’s. (C-GDDI2)  Well you’re right they have causal mutations (C-GDDI-Ex1)  Well they will develop this amyloid deposition. (C-Mod)  So is worth asking sensitivity, specificity question other than enriched population, people that are homozygous or heterozygous for APOE4? (C-GDDI4)  Yeah I mean that was my only question about genetic issues whether the APOE4 is it worth asking but not in any of the other genetic, (inaudible) the actually causal genes but in the APOE for risk factors is it worth it. (C-Mod)  So I guess among Caucasians about 25% of people will have an e4 APOE allele. With that being said it’s also e4’s an independent risk factor for Lewy body dementia in addition to Alzheimer’s disease. (C-Ex1)  Not informative (C-GDDI4)  It’s probably informative, but it’s narrowing again down to a population that already by virtue of having had that testing done has some increased risk presumably unless they just to 23&Me which you’re patients may… (C-GDDI-Ex1) |
| Physicians as study subjects +/- PICO population | Yeah so the IDEAS study was so Medicare when they um, decided not to provide coverage for amyloid imaging did…I think you mentioned it earlier was that they did provide avenues for um, paying for amyloid scan in the service of learning more about their impact on outcomes and I should say also like Medicare’s decision was not based on…their coverage decision was not based on the accuracy of the test or the, you know the, um, even the clinical it was based on the economics to some extent of it and its impact overall on outcomes and so the IDEAS study is being paid for by Medicare to obtain amyloid imaging in 18,000 some odd people across the country, um, with the FDA approved agents to mimic what clinical practice would look like um, for using amyloid imaging following the appropriate use criteria which was essentially criteria who we feel amyloid imaging will make the most impact on and whether looking at…they’re actually studying so like for us to do the study let me just say we actually have to sign a consent because they are actually studying us and how it impacts our behavior. (P-Ex2)  We’re the tests subjects (P-Ex1) | Are physicians a population that we’re interested in? So IDEAS is structured to…so it’s not the patients that are the participants, it’s the physicians that are. So we’re consented as study participants… [In] the IDEAS study, so we consider physicians as a patient population in terms of their decision making ability or whether they’re…I guess we’re not going to have…there will be no studies that are looking to my knowledge that look at physicians satisfaction whether this provides them better confidence in their ability to make that diagnosis or anything like that. (C-GDDI-Ex1)  It would be…and it’s sort of two questions. Does it increase their confidence and should it increase their confidence. And it probably does the real question is should. (C-Method)  So we had one of the populations, I think maybe…I don’t know who brought it up, and the populations was physicians and their confidence or they’re treating differently based on a positive or a negative scan and so I still think that perhaps some of the question 2 and question 3 may answer that but I’m just wondering. (C-Mod) |

Table 6. Themes and Exemplary Quotes Relating to Comparators for Guideline (“C” in PICOT)

*In general, Group 2 spent a lot more time discussing the nuances of comparators/reference standards than Group 1*

| Theme | Gp 1 (Exper, WITH PATIENTS) | Gp 2 (Control, PHYSICIANS ONLY) |
| --- | --- | --- |
| Discussion of reference standard in general |  | Then when we say…when we talk about diagnostic accuracy and reference standard, we’re just going to say a reference but we mean all those things we talked about. I won’t list them. But we mean the presence of dementia is clinically determined, the development of dementia over some specific period of time, the pathology, the presence of amyloid alone, the presence of amyloid plus other changes that you would expect to see in ADD. So all those things is what we mean by the reference standard. (C-Method)  **Different standards will provide stronger or weaker evidence**  So it could potentially be any of those things and some of them will provide stronger evidence, some of them will provide weaker evidence based on which ones they selected. Like if they did the volumetric MRI based on what you said it’s not going to be all that great of the evidence because it’s not a great reference standard. (C-Method) |
| Clinical evaluation alone as comparator |  | Well it’s going to…it’s going to have to be the clinical evaluation. I think usually it’s going to be some evaluation by dementia experts although we may be neurologists or geriatricians or something, but I think it’s going to be, we sent them and they did their thing and this is what they thought, they thought they had it, they thought they didn’t and we also did PET and then we showed them the PET and then they changed their mind or didn’t change their mind and then we compared that to some of the reference standards that we mentioned. (C-Method)  **Development of dementia as reference standard**  So I think there are two um issues to the clinical evaluation: the presence of dementia or the development of dementia. That could be two separate reference standards that we might run into. They have dementia now or (C-Method)  So you’re talking diagnostic and prognostic? (C-Mod) |
| Not doing scan as the comparator | Ok so the question is it doing it versus not doing it? (P-Ex1) | The “C” the comparator intervention is the No amyloid PET, it’s not nothing but it’s…because people still are going to be diagnosed with AD, they’re going to be down here they’re just not going to have the benefit of the PET, they are still going to be diagnosed. And then the “O” is all the outcomes listed, Ok. So it’s all these things, Ok. The only study, and then it’ll probably be out I don’t know by the time we’re done with this. I don’t think it even approaches (talk over), is the IDEAS study. But who knows maybe we’ll be surprised and we’ll find something down in Eastern Europe that is exactly, you know answers this question. So then…but if we don’t want…we have to explicitly say this is the kind of evidence that’s needed and we don’t have it yet. So this is question 1 Ok. So now we…we’re not going to…if we found question 1 and we had study evidence for that you know you could be done. So this is…that’s called the utility question basically it’s the utility question because we’re using a diagnostic test but we’re trying to see the benefit of actually using or not using the diagnostic test so the randomized controlled trial on prostate specific antigen for example, is an example of a utility question. And they are evaluated by the therapeutic criteria, Ok, that we have. All right. We probably won’t find evidence for this. (C-Method) |
| Pathology as the reference standard |  | - So in terms of pathologic confirmation I mean this is sort of two questions. Does PET actually identify amyloid pathologically I mean you could do that. But then is that sufficient for the pathologic diagnosis of Alzheimer’s disease. Do you need more than that? (C-Method)  - And we’re going to be challenged because in 2012 the criteria for the pathologic diagnosis of Alzheimer’s were modified and now are ultra sensitive at that…it’s a range so between low, moderate, and high…low, intermediate, and high and no probability of AD neuropathologic change causing dementia. It’s very…it’s a bit of a nuance criteria so that’s from 2012 to…now that we use immunohistichemical techniques to detect amyloid I suspected if we repeated the study we would find that there…sometimes we were detecting amyloid when the amyloid PET scan doesn’t. In that earliest group now is that…those people have Alzheimer’s neuropathologic change but probably would be a low probability that that change predicted the clinical state. So it’s going to get a bit messy. (C-GDDI-Ex1)  - I think we’ve just got to keep it, like does it detect amyloid, you know what I mean? (C-Ex1)  - Well you can do both because we can kind of see what we find, uh but it would…I see one question that could be settled relatively rapidly is that this technology is very accurate at detecting the presence of amyloid, period. And then they’ll have some probably lessor accuracy of protecting or of detecting Alzheimer’s disease pathology, uh, amyloid plus something else. Something sufficient and probably even less if you added dementia to that pathology and then you know you get various results. So I think it’s good to break it down this way because when we’re looking at the studies we can do that and… (C-Method) |
| Comparing to other tests | So far we’ve been talking about amyloid imaging versus no amyloid imaging. Then you’ve got the question of amyloid imaging versus other ways of answering these questions such as you mentioned the CSF. So you know do we need to do a comparison of that because Ok what if we…what if we come up with it in a finalizer, yeah well amyloid imaging you know does one or more of these but what it if turns out one of the other alternative tests is better, cheaper, less harmful. (P-Ex1)  More accessible (P-Moderator)  I mean if you do an LP anywhere effectively it seems so. I that’s that in important… I mean so that’s a separate question (P-Moderator)  **Amyloid alone might be predictive but not as good; need to consider in context of other tests**  There are these multi modality studies now where there are more direct comparisons, um, but um, that’s more limited… There are actually sort of a three or four candidate biomarkers that have been used like you know I would argue, you structural hippocampi binders is the first raw study so you can sort of…if you want to compare it against… Yes I do think it’s an important question though because I do feel like if you come down on some sort of a recommendation on amyloid imaging in isolation, whether this is the actual question or something that would be in a discussion, um, I do think it’s…it’s definitely a real issue like would an MRI that showed hippocampal atrophy in a mild cognitive impairment patient provide you with the same relative prediction of decline as amyloid imaging. Um if you looked at amyloid imaging alone you’d say Yes it definitely predicts, I mean there’s lots of data that predicts outcomes. When you get in to hippocampal volumes there’s lots of data that say it predicts outcome, so you know from a cost (P-Ex2)  **Limit guideline scope by considering just head-to-head comparisons**  So methodologically one way to limit that and to make it manageable is to only accept head to head studies instead of like here’s this cohort said that, and here this other cohort said that, two separate cohorts and now we’re going to compare it, that becomes impossible in my opinion (talking over) (P-Method)  I think if…I think that’s a good uh compromise, you know including head to head studies or finding that there are none and calling for them, uh as opposed to trying to look at every other study which will probably be under the dementia one that’s going to go for a decade anyway. (P-Ex1)  **Combination of markers may be better than one alone**  The reality is what the head to head studies show is that the combination of mile markers was better than any one alone generally and that the more that you have that or (inaudible) the higher the risk is, but there are some…I mean you know there’s a strong argument that for example the FDG PET actually is a better predictor of decline than amyloid imaging is. It might not be as specific with, you know if you had to pick which is the one that’s really going to tell you have Alzheimer’s disease and there are head to head studies but it is a big thing to chew on. I was just thinking whether even just in a discussion that that would be something that would be brought up as opposed to the actual question you’re trying to (P-Ex2) | I like the analytical frame work in that sense that maybe you know that compare your patients with MCI does amyloid imaging versus you know neuro psychological testing lead to any difference in health outcomes or amyloid imaging versus the standard, you know whatever standard clinical evaluation you know which would include all the stuff to do. Just add, you know does that lead to any difference. (C-GDDI-Ex2)  Well, that’s, you know that’s the big utility question (C-Method)  [In discussing standard evaluation as comparator]  So the standard evaluation could be anything. Uh that the study says. It could be one of those reference standards, it could be a combination of the reference standards. C-Moderator  **Comparing to CSF**  I guess what would be is…I guess you’d want to compare it to…you’re looking at what predicts the amyloid. I would…the Tau is not necessarily, you know sorry to say that you know the CSF a beta versus amyloid PET as the comparison not take into account…because then you would want to look at amyloid PET plus FDG PET or CSF a beta and total Tau, you know what I mean? Because you’re looking at a neuro-degeneration marker and an amyloid marker and so I think you want to compare amyloid to amyloid. (C-Ex1)  Well but here it’s not as…not so much comparing amyloid to amyloid as it is the total CSF picture that sort of suggests to you that this person has AD versus anything. So sort of the reference standard for the diagnosis of AD rather than what does amyloid there mean in terms of amyloid PET imaging. (C-Mod)  So that means it’s all in how we phrase that, you put the precursor that in patients with dementia suspected to be Alzheimer’s disease and because what’s more sensitive is it CSF biomarkers or versus amyloid PET. Because the view point of that would be…amyloid can be decreased for a number of reasons that aren’t necessarily related to Alzheimer’s disease. Maybe brain inflammation probably decreases amyloid levels as well. So you might take it in a bigger…in a broader context. (C-GDDI-Ex1)  *No standards for CSF*  We may have to do it as in studies because there’s no INR for CSF (talk over) and every lab is different.  **Comparing to other imaging**  Volumetric MRI (C-GDDI4)  **Possibility of combo reference standard**  So a related question. Are you likely to see evidence that look at only one of these as a reference standard? I have a feeling that you may have clinical, perhaps, I see only one over here that would be the sole reference standard in some studies. Otherwise maybe biopsy but CSF I have a feeling may be combined with clinical, I mean would there be studies that look at CSF alone as a reference standard? (C-Mod) |
| Different tests may be better for different things | What do we care more about in terms of prognosis for functional decline versus getting the pathology right and I think that that always comes up when we’re kind of comparing these different types of biomarkers, um, is that it could very well be the case that for example structural MR maybe more related to where someone is, what their likely future is with regard to symptoms but it might not predict what the underlying pathology is when a, you know someone looks and does an autopsy. So what’s more important to the patient and what’s best at sort of making a prediction and some of that is in the context of just what our current treatments are. If we have a amyloid specific treatment then it may be more important um to get that thing right where as if may be more important now that we don’t have that in the context of the patient to know what is the thing that’s going to best tell me am I going to have functional decline a year, two years, three years from now and maybe there’s the kinds of comparisons that we can think about trying to make. (P-Ex2)  So I mean I do think that there’s difference between how well these tests do in predicting cognitive impairment due to Alzheimer’s versus just cognitive impairment at large and I think that again you know I can imagine this is why I brought up that sort of third question which I don’t want to add, I do think it is too many questions but is that one biomarker may be better at predicting the likelihood that in 5 years you’re going to develop symptoms and that might be what structural imaging. And amyloid imaging might do worse in that but of the people who…that it predicts to develop dementia it will be enriched to people who it’s due to Alzheimer’s disease, um, but I would just do it as cognitive impairment. Especially in controlled…and I think that…I mean I don’t know. (P-Ex2)  We’re talking about the specifically measures here which are single things so I, um, I’m not…I think that um, if you’re talking about predicting developing dementia due to Alzheimer’s pathology then there’s do doubt that amyloid imaging and CSF A-beta will do better than anything else. Because you’ve already defined to that group as having amyloid in their…you know having Alzheimer’s disease pathology, um, and if they decline it’s going to be very hard for most clinicians even very savvy ones if they decline and it looks atypical for them to say that that’s enough for them to say it’s something else that’s going on other than Alzheimer’s disease. (P-Ex2)  Yeah. So like you know so there’s a lot of data again that neurodegenerative markers like structural changes and even FTG PET are not good at predicting amyloid pathology. We have a paper that specifically looks at those um in preclinical Alzheimer’s disease and amyloid. So amyloid pathology was much better. In later stages of disease when there’s more manifest neurodegenerative change these other kinds of markers which are really marking the down stream affects of the disease do better at predicting whether or not there’s amyloid pathology. So stage does influence that. (P-Ex2) | So the amyloid imaging changes occur before the CSF biomarker changes. (C-GDDI4)  CSF occurs first, but it’s harder to measure unless you measuring serially too. That’s the other thing. For CSF. (C-Ex1)  That may be a questions I…for predictive purposes which may be better, serially imaging or serial biomarkers. (C-GDDI4)  There’s a study that was in Neurology not that long ago but that’s enrolling patients that you think have dementia and doing both CSF and amyloid PET and comparing them, cross sensitivity. So I mean the most common one is going to be you do it cross sectional analysis of the biomarker CSF or amyloid and then you do a longitudinal clinical follow up to establish whether they continue to meet the description of AD dementia or where they develop dementia or whether they resolve and go on to something else and then you see how did that, you’re cross sectional…your look with your test, how did that predict. (G-GDDI-Ex1)  So your reference standard is a clinical reference standard and you’re comparing these two diagnostic (C-Mod) |
| Unacceptable reference standards |  | Are any of these reference standards so bad that you wouldn’t want to use them to validate the diagnostic accuracy of the amyloid PET…that they’re just so unreliable. For example clinical evaluation so unreliable that it’s not a valid reference standard, um, you know should we decide that up front. I mean you have to make that decision independent of (C-Method)  Right. I think the ones that we picked are ok. The least reliable would probably be volumetric MRI. (C-Ex1)  Right. And then FDG PET probably the least. (C-GDDI-Ex1) |

Table 7. Themes and Exemplary Quotes Relating to Outcome for Guideline (“O” in PICOT)

| Theme | Gp 1 (Exper, WITH PATIENTS) | Gp 2 (Control, PHYSICIANS ONLY) |
| --- | --- | --- |
| What are the most important outcomes | [After P-Mod mentions diagnostic test performance] I guess though, I can understand the questions about testing, but I turn that around and say Ok what are you testing for. In a sense to me, the question really is Ok can you answer questions that will help me live a better life or you know help me reduce the impact of the disease. So I mean part of it is just tell me I…you know I have six months to live or six years. And the other one is, you know what is the progression going to be and is there anything I can do to intervene in that. (P-P1)  One of the things I guess to think about is um, maybe how you…in terms of how you might formulate the question is: What do we care more about in terms of prognosis for functional decline versus getting the pathology right and I think that that always comes up when we’re kind of comparing these different types of biomarkers, um, is that it could very well be the case that for example structural MR maybe more related to where someone is, what their likely future is with regard to symptoms but it might not predict what the underlying pathology is when a, you know someone looks and does an autopsy. So what’s more important to the patient and what’s best at sort of making a prediction and some of that is in the context of just what our current treatments are. If we have a amyloid specific treatment then it may be more important um to get that thing right where as if may be more important now that we don’t have that in the context of the patient to know what is the thing that’s going to best tell me am I going to have functional decline a year, two years, three years from now and maybe there’s the kinds of comparisons that we can think about trying to make.(P-Ex2)  I would um, pick a non amyloid outcome that you’re looking for, that you’re not trying to predict amyloid or Alzheimer’s, you’re trying to predict clinical outcomes, cognitive decline or progression to dementia if we’re talking about people who are…and to me that is a very relevant question for the field. (P-Ex2)  So again I agree that it’s a different, it’s just what question you want to ask. So do you want to ask about what biomarker is best at predicting Alzheimer’s pathology and we know the answer to that, it’s you know either CSF abeta or amyloid path or what’s the best at predicting…I guess there’s three choices right. So what’s the best at predicting pathology. So that’s going to be…that’s one question. What’s the best at predicting a functional or cognitive outcome which um in this day and age of not having specific treatment for these different conditions is not an unreasonable question to pose. What’s the one that if you’re seeing the patient I can say to you in three years you’re going to start to have problems with your function, you’re going to need more help at home, you’re not going to be able to drive or something of that nature. So that’s another question. And then the third would be what’s the likelihood of having Alzheimer’s dementia which is dementia due to Alzheimer’s pathology and I think you could ask any…those are all completely legitimate relevant points. I would argue the first one is the least interesting because I think that it’s…the data…I mean I think we…there’s very good data with regard to…uh and it’s not that it’s not data we could summarize but there’s a lot of data showing the concordance between (inaudible) measures and pathology. (P-Ex2)  Which test is best at detecting Alzheimer’s or amyloid pathology and then which test is best at predicting progression of dementia and both of those are important both in terms of knowing what is causing the problem or knowing what you’re, you know, future risk is in planning. Both of those are important. But then the other question brought up was which is best at predicting a dementia due to Alzheimer’s which I think is the least…I think that’s the least one. (P-Mod)  No because the first one is for the risk issues we’ve sort of talked about that you can have Alzheimer’s pathology and you know we can debate how many years it would take or whether everyone would go on to develop the disease, but I can…if I tell a patient you have amyloid pathology in your brain and I can’t tell them whether or not they’re going to progress to dementia within their life span (P-Ex2)  But that’s answered by the first question right? If they don’t have the amyloid pathology, if those tests are very good at detecting amyloid pathology you can say you are very unlikely to get Alzheimer’s disease, right? I don’t know, and if it’s positive we don’t know when you’re going to get it. That’s fine, but it’s still…right I mean knowing if that…So I think the first question is the most important and the second questions is definitely important, but I…maybe that is the third question that we don’t have to address because we can’t or it’s at least part of the first question. (P-Method)  I’m just saying, so the second question would address whether or not you’re going to progress to dementia but it’s agnostic as to what the dementia is. The third question. So that there is like, I mean it’s…which is fine. Again to me the most relevant in this day and age right now it’s the second question which is what’s the likelihood that you’re going to develop more symptoms. I think from a management perspective is the most relevant. Um, I think the first question is very answerable, it would be nice to summarize like how well do these tests do in terms of actually detecting amyloid pathology. To get FDA approval that’s basically the process that these tests went through, um, is the autopsy based measures. (P-Ex2) |  |
| Predicting underlying AD pathology | I guess the first question is just how accurate is the test at predicting underlying Alzheimer’s pathology… So the first question is a pathologic one. It’s how well do these tests do at predicting underlying pathology. To me that is the juice [not?] worth the squeeze for that. To me that’s not…I mean I guess I accept all the data that is out there that I’m not sure that creating guidelines that answer… That would be a softball question, I mean that would be one that, there’s a somewhat limited but…or constrained literature on address that particular question. Um, it’s a very tractable question to ask if that of value. (P-Ex2)  You’re asking does it…how well does the test do at predicting Alzheimer’s pathology. I don’t know if that needs to be broken down based into pre-clinical, prodromal, or AD dementia. It’s more just how well does an in vivo test of the scan match up to pathology. (P-Ex2)  Does the amyloid imaging predict amyloid pathology, yes. Does the amyloid imaging predict, detect Alzheimer’s pathology, well now we’re expanding to amyloid and Tau potentially. (P-Ex1)  I thought we were just asking a simple question of how well does amyloid imaging do for predicting amyloid pathology. That to me is irrespective of stage. (P-Ex2)  In dementia it would be relative to what…or to…for prediction of AD pathology. Is that what we’re predicting? (P-Ex2) | I mean you can ask the question, Does amyloid PET accurately identify patients with brains with amyloid. All right? And I suspect the answer is yes. (C-Method)  I see one question that could be settled relatively rapidly is that this technology is very accurate at detecting the presence of amyloid, period. And then they’ll have some probably lessor accuracy of protecting or of detecting Alzheimer’s disease pathology, uh, amyloid plus something else. Something sufficient and probably even less if you added dementia to that pathology and then you know you get various results. (C-Method) |
| Amyloid does not tell you about dementia severity | And I think amyloid imaging is helpful in determine…in context in giving you a little bit more clarity on you know prognosis and specific diagnosis. But it’s going to miss the boat on you know severity. (P-Ex1) | Or screening for severity I think or assessing severity (C-Mod)  Right, right so assessing the severity of dementia since amyloid levels plateau in the MCI phase of the disease so you can’t ever get a severity with amyloid burden and also for work related questions so they also said that would be an inappropriate way to use it so if someone’s having issues at work or something like that. (C-Ex1) |
| Value in differentiating types of dementia |  | Is it necessary to repeat the question about, or any of the questions about differentiating the types of dementia? Because if you could take that our of the guideline it would be very easy. I mean it would save a lot of work because why do we have to look again at Alzheimer’s versus Lewy body versus vascular? (C-GDDI1)  Well I think it’s an important question because that’s one of the reasons for that FDG PET is paid for by CMS is to distinguish its (talk over) (C-Ex1)  It sounds like it sucks (inaudible) (C-GDDI1)  But it does, so what FDG PET pays for at CMS is to distinguish AD from FTD which is very important for families, even though there is not a specific treatment a proper education as to the disease has saved many families, but um, that’s where amyloid PET will be most helpful I think and where it should be looked at is AD versus (talked over) (C-Ex1) |
| Accurate diagnosis | **Improving diagnostic certainty**  Outcome is improving diagnostic and I don’t know what the right word was, certainty for present Alzheimer’s pathology. And then we’re probably trash that. And/or certainty about prognosis to progressed Alzheimer’s dementia in a time frame. (C-GDDI-Ex1)  **Debating outcome of dementia in general vs AD dementia**  It could be that one test is very good at predicting…could be that hippocampi lines is the best test for predicting whether or not you’re going to progress to dementia if that’s the questions. But it may be that the amyloid test is the best test at predicting whether or not five years you’re going to progress to Alzheimer’s dementia… Even though it might not do as well in predicting whether you’re going to progress to dementia because it’s more specific (P-Ex2)  **Diagnosing cause of dementia**  I think we get at the diagnosis…to me the diagnosis….where we get at the diagnosis is in the dementia question, Alzheimer’s dementia refining versus something else. (P-Ex2)  It can tell you are you at risk for getting Alzheimer’s, are you not at risk for getting Alzheimer’s compared to the general population, do you have Alzheimer’s if you’re already demented or do you have something else, but it can’t tell you what else. (P-Ex1)  for dementia we should have questions asking for the head to head does amyloid scan head to head versus other tests better predict Alzheimer’s…or better determine type of dementia (P-Ex1)  **Debating outcome of AD pathology vs AD dementia**  The second half of this do we want to say it predicts developing cognitive impairment due to Alzheimer’s pathology or just Alzheimer’s disease dementia? (P-Mod)  The only thing I guess, how would…I don’t even know how one would answer developing cognitive impairment due to Alzheimer’s pathology. How would you know that…if I got an amyloid test now, um, and it’s positive and in 5 year, from now I develop dementia, it could be something else. (P-Ex2)  Well in the review you’re looking at how well it does defining Alzheimer’s pathology, I mean (P-GDDI-Ex1)  That’s right and then clinically you take that information and say its Alzheimer’s dementia (P-Mod) | **Diagnosing cause of dementia**  So is it fair to sort of summarize what you said into two big baskets. So one is take the symptomatic group and figure out whether they have amyloid pathology or they don’t (C-Mod)  The cause of their dementia (C-GDDI-Ex1)  Right, exactly. So figure out whether they have amyloid pathology which will say they have amyloid disease dementia. (C-Mod)  …accurately diagnose AD, ADD Ok. This will be most of the evidence that we look at, is going to be related to that. (C-Method)  the primary clinical use and thinking is trying to figure out the cause of people’s complaints usually or the cause of what we as clinicians determine to be cognitive impairment. (C-GDDI-Ex1)  **Asking a diagnostic accuracy question**  So that’s the diagnostic accuracy. So in patients who are already symptomatic whether it be dementia, whether it be atypical dementia or MCI, do you want um…and dementia here will include ADD, right? So I’ll put that down here as a separate category so we are assuring including those as well. So patients who have clinical AD, what is the diagnostic accuracy of amyloid PET in patients who have dementia, what is the diagnostic accuracy, atypical dementia and MCI. So we are looking here at sensitivity, specificity, PPV and PV. (C-Mod)  **What is the incremental benefit of adding amyloid PET for diagnosis**  Now there is a closely related question to that which I hope we find some studies for that will compare the incremental value of adding PET to this so this we’ll call question 3. So look at the incremental increase in accuracy you get if you add PET and I’ve seen studies like that I think we’ll find stuff like that. Um and the question there is…so this is Q3 so for patients at risk for ADD does amyloid PET added to standard evaluations, compared to standard evaluation alone, increase diagnostic accuracy as compared to some reference standard. There’s always an independent reference standard. So this is very closely related and some of the articles that will meet these criteria will answer that question as well. (C-Method)  We don’t know at least in patients with…maybe we don’t know for any population, but at least the patients that seem to have typical Alzheimer’s disease clinically, we don’t know whether there’s added information provided by this. And the interesting thing about that is when you come to a diagnostic test then if you don’t know that there’s add information, there is no added information. That’s just sort of a… (C-Method)  *Incremental benefit may be more from negative test result than positive test result*  So I’ve enrolled people who have dementia, who I told them have Alzheimer’s disease dementia by all clinical criteria… and their amyloid PET is negative and that patients population that’s a hugely important finding because now I have to really go back and unearth any possible disease to follow up on that… now I’m back at square one because that negative scan is very important in that population. The positive scan would not have had any incremental value for me. (C-GDDI-Ex1)  **Pathology vs clinical**  The PET scan is detecting we think pathology but it’s the clinical manifestation that we’re interested in as neurologists (C-GDDI-Ex1) |
| Accurate prognosis | How many…how good are they at detecting risk of cognitive decline and there are going to be different answers obviously but I think that…and we don’t have to ask it for every subtype of patient population. (P-Mod)  What is, I’m a patient I want to know what’s the likelihood I’m going to develop dementia in a few years. Give a suite of tests that you can get but amyloid is one of them. What is, how much…how well does amyloid predict whether or not I’m going to get dementia in the future. (P-Ex2)  **Linear or categorical cognitive decline**  So often times it’s looking for either cognitive decline in a kind of linear way or continuous fashion or looking at cross the threshold into mild cognitive impairment or Alzheimer’s disease. (P-Ex2)  **Prognosis in cognitively normal individuals receiving test**  So really there’s two questions there, it’s you know…and one is sort of outcomes and the other is validity. You know 1) does it provide an accurate enough prognosis and 2) does knowing that prognosis improve or diminish the quality of life for the individual. (P-Ex1)  All right, so for pre-clinic…this is what I’d say. For pre-clinical being able for amyloid to predict progression to Alzheimer’s disease because I don’t think it’s going to predict anything else is reasonable and the evidence is already there. Um, so it’s…in a way it’s a softball. Um, you know again we have to say look at, you know harms and risks and benefits for that. (P-Ex1)  **Prognosis in people with MCI**  So and let me take that one step further, if we were to actually know the amyloid pathology presence or absence, you could chart out a prognosis, it would be different between those two groups. Amyloid positive or amyloid negative among MCI patients. (P-Mod)  Well there is data saying that the combination of looking at the pattern of deficits plus a biomarker does add to your sensitivity and specificity so it could be amyloid in some cases, it’s volume metric, uh, MRI, um you know others using CSM biomarkers, um so combining those two because while they both are predictive they are not one to one, they overlap so combine the two increases your prognostic accuracy. (P-Ex1)  I think the biomarkers as well as cognitive testing increase your prediction accuracy for um, the likelihood that someone is going to progress to more significant clinical impairment (P-Ex2)  For predicting like in a mild cognitive impairment patient whether in 5 years you’re going to develop more significant cognitive symptoms. (P-Ex2)  A patient is, you know how many years will it give you before I’m going to have significant functional decline. (P-Ex2)  If you have a positive amyloid scan and you’re amnestic mild kind of impairment I’ve not got a very high likelihood that within a few years you are going to have Alzheimer’s disease. In fact as far as I am concerned, you probably have Alzheimer’s disease you just haven’t crossed into the dementia stage yet where as if you have a negative amyloid scan, I’m not sure. I’m not sure now where you’re going to end up, you know you might get another type of dementia or you may never get a dementia, we’re just not sure. (P-Ex1)  **Prognosis in people with dementia**  I think that the two choices are predicting rate of progression if you want to do a clinical one or pathology if you want to do a…you know which one is more... I mean I actually I mean I would say from a um…just from an educational standpoint, um, that this comparisons is useful for people to know about because I do think there’s a misconception that amyloid PET tracks the disease. I think what we would find if you looked at the literature is that amongst um, Alzheimer’s disease patients markers of neuro degeneration better predict change overtime than amyloid does and so I think from a standpoint of providing new information for people that I can imagine a useful way of framing the question. (P-Ex2)  **Can we know if cognitive decline is due to AD versus other contributors if the amyloid scan is positive [which is appropriate outcome, cognitive impairment in general vs cognitive impairment due to AD]**  Accurately predict development cognitive impairment… That is the right question. (P-Ex1) [question also said “due to Alzheimer’s disease”]  So how would you know if I get…if you get an amyloid scan on your patient then five years later developed cognitive impairment, what would be your test to know that it was due to Alzheimer’s disease as opposed to that they developed something else? (P-Ex2)  Well the first test would be their cognitive history, exam picture, what problems are they having, do they fit the clinical picture of Alzheimer’s disease and that’s how you answer for it. How would you predict it? (P-Ex1)  No I would say I wouldn’t…I would say I’d be…I just think the due to Alzheimer’s pathology is…what any of these studies are going to look at is whether there’s been cognitive decline. We’re not going to know if the Alzheimer’s pathology is what’s driving their symptoms 5 years form now so that’s just part of the noise of any of these measure is that there are lots of things that cause cognitive…I mean we see this all the time in mild cognitive impairment. There are patients who are amyloid positive who don’t progress, don’t have a pattern of Alzheimer’s neuro-degeneration and it’s likely was that they just had pre-symptomatic Alzheimer’s disease and that’s not what’s driving their cognitive symptoms. They also might have something else and so…I don’t know just from a sort of scientific standpoint and like a question that’s addressable scientifically I just think it’s hard…now if you want to define it as clinical Alzheimer’s dementia then I’m with you, then you could say (P-Ex2)  Look this is what I’m going to tell you. You can get cognitive impairment for other things, they may get hit by a bus, but that’s not what you’re getting the amyloid study for. You’re trying to predict their risk of developing cognitive impairment due to Alzheimer’s (P-Ex1)  But that’s what we’re going to get. We’re going to get the answer about cognitive impairment and then you can intuit what you think the cause was, but the only answer we have is whether they get cognitively impaired or not and they you can call it Alzheimer’s which is probably is, Alzheimer’s disease dementia for now but the point is we’re only going to look at cognitive impairment and then whatever you want to do with it (P-Mod)  I mean that’s what you can measure at that point. I don’t know… To me that is the noise that I mean, this is the noise of the neuro degeneration is that there are lots of things that cause cognitive decline so any tests that we have, part of the issue of it’s predictive value and our specificity is the fact that there are other things that also can be concomitant with it. And so to me that is why you know it’s not a 100% when you’re amyloid positive and you have MCI that you’re going to go on to develop Alzheimer’s disease within a couple years because there are other things that cause it so I just think it if safer and more answerable to just say future cognitive impairment (P-Ex2)  That’s all we’re measuring. That’s all we’re going to measure is cognitive impairment. We will have that answer and how you interpret that whether you’re going to call it Alzheimer’s disease or you’re going to say its probably Alzheimer’s disease. Nobody cares. If the only thing that matters is that we have the cognitive impairment answer for patients, right? (P-Mod)  And I don’t like it but I will bow to the wisdom of the group (P-Ex1)  I think that um, if you’re talking about predicting developing dementia due to Alzheimer’s pathology then there’s do doubt that amyloid imaging and CSF A-beta will do better than anything else. Because you’ve already defined to that group as having amyloid in their…you know having Alzheimer’s disease pathology, um, and if they decline it’s going to be very hard for most clinicians even very savvy ones if they decline and it looks atypical for them to say that that’s enough for them to say it’s something else that’s going on other than Alzheimer’s disease. I just…I think we get into where it becomes hard to answer the question with scientific data. That’s really not…we’re biased and so in my mind I guess, I still feel like saying…predicting decline to dementia rather than defining (talk over) (P-Ex2)  Really again it’s specific to developing Alzheimer’s dementia. (P-Ex1)  Having the Alzheimer’s pathology and seeing somebody decline, when do you decide that’s Alzheimer’s dementia? (P-Mod)  …I think it’s hard… (P-Ex2)  You have real life impairment, you have a cognitive pattern that looks like the types of places that Alzheimer’s hits typically and you’ve got a positive scan you’re going to call it Alzheimer’s disease unless you get an autopsy later on that proves you wrong. (P-Ex1)  **Multiple things can drive decline**  No I just…again I…to me the…yeah I feel like um that is hard to disentangle um and so I think…and that is part of the noise of the measure is that it is because sometimes there’s other things that drive declines and to me it’s like part of why its accuracy will be limited in predicting rate of decline is because it’s hard to know how much the vascular disease that person has is also contributing, how much that…(inaudible) and pathology for that patient. (P-Ex2)  **Amyloid may not be best marker for progression to dementia**  The ability of amyloid to predict progression to dementia, it’s going to be a mix and there’s probably other markers that will be better for all cause dementia. Um, so there I think it’s going to be less usable um, now for the ability of…what was the third one now…so amyloid to predict progression to Alzheimer’s disease, amyloid to predict progression to all cause dementia  **Is amyloid PET necessary if we just want to know about progression to dementia in general**  Why are we doing any of these biologically specific tests at all if all we want to do is see if people get to dementia. I’m just raising the devils… (P-GDDI-Ex1)  I think that’s a great question right now and that’s why, you know in this day and age, um, without treatments from a clinical management standpoint, it may…one could argue um, that uh…that it’s better to predict dementia and/or cognitive decline than it is to predict Alzheimer’s disease dementia. (P-Ex2)  **Debating outcome of predicting AD dementia vs any dementia**  I think that um, if you’re talking about predicting developing dementia due to Alzheimer’s pathology then there’s do doubt that amyloid imaging and CSF A-beta will do better than anything else. Because you’ve already defined to that group as having amyloid in their…you know having Alzheimer’s disease pathology, um, and if they decline it’s going to be very hard for most clinicians even very savvy ones if they decline and it looks atypical for them to say that that’s enough for them to say it’s something else that’s going on other than Alzheimer’s disease. I just…I think we get into where it becomes hard to answer the question with scientific data. That’s really not…we’re biased and so in my mind I guess, I still feel like saying…predicting decline to dementia rather than defining (talk over) (P-Ex2)  So what questions can you ask about the mild cog impairment. Are you going to get Alzheimer’s dementia, are you going to get any dementia, and how can I tell which dementia I have. Ok. The amyloid imaging it not going to…is going to only tell you the part of your risk due to Alzheimer’s disease and it’s not going to tell you what other types of dementia you might have or if you will get a dementia, period. (P-Ex1)  So I think that that’s right and that it’s…so I think again, three…the problem is when you go into your doctor with those symptoms and you have an amyloid negative scan I can tell you have a…or an amyloid positive scan I can say you have a 60% chance that you’re going to develop Alzheimer’s or you’re going to develop dementia which I think will like be due to Alzheimer’s disease. If you have an amyloid negative scan you still have a 25% chance of developing dementia due to something else. So to me, I don’t know so it depends on what…how you want to (talk over) (P-Ex2)  I mean as a patient I just want to know am I going to get dementia, memory loss. (P-P1)  I mean that is the sense that I get from a lot of patients and so that…and that is the discussion I have with a lot of patients is (P-Ex2)  Because the type depends on how you treat it, but the response to me is the same, I’m losing my mental ability. (P-P1) | **Prognosis in cognitively normal individuals receiving test**  *Dementia as outcome vs AD dementia as outcome*  We are looking at the outcome and the outcome here is how many of them become AD and how many of them don’t become AD no matter what that group is and then we part the other group out. (C-mod)  I guess I would keep it broader for this really, how many get dementia, commonly convert to dementia and then a subset of that will be AD just you a minor difference. (C-Ex1)  So progression to dementia… So in an at risk cohort does amyloid PET imaging predict progression to dementia.(C-Mod)  And then they’ll capture those with Lewy body as well (C-Ex1)  I mean in a useful world amyloid imaging would predict a higher risk for progressing to AD we’ve predicted…had no predictive value we wouldn’t find it a very useful test. (C-GDDI-Ex1)  you scan all these guys, you have positive/negative, you follow them, some in each group will convert to AD others won’t and to your answer is going to be proportion our relative risk of, you know or odds ratio if you will of converting to AD and you know in those guys who have a positive scan. (C-Mod)  **Prognosis in people with MCI**  it makes you also wonder um you know you could have MCI for a long period of time, is it also useful in predicting the conversion to dementia. So if you are MCI amyloid positive will you convert in two year or is it if you’re MCI amyloid negative 10 years. You know what I mean? So is it useful for prognosis. (C-Ex1)  Because the MCI group has people that revert, it has people that progress to dementia. We say like 10% per year convert (talk over) (C-GDDI-Ex1)  It could also be if your MCI had amyloid negative what is your chance of reverting back to normal? Or not progressing either way. (C-GDDI4)  Right but your outcome is going to be (C-Mod)  Clinical progression (C-Ex1)  The question will sort of be the same it could be what happens to them (C-Mod)  What is the progression (C-Ex1)  What is the progression to ADD (C-Mod)  I guess what I was getting at is, yeah it may not be AD they are converting to (C-Ex1)  **Predicting AD dementia vs any dementia**  I guess what I was getting at is, yeah it may not be AD they are converting to. (C-Ex1)  I guess I would keep it broader for this really, how many get dementia, commonly convert to dementia and then a subset of that will be AD just you a minor difference. (C-Ex1) |
| May be better for diagnosis than prognosis  *(see also section that different tests do different things)* | one biomarker may be better at predicting the likelihood that in 5 years you’re going to develop symptoms and that might be what structural imaging. And amyloid imaging might do worse in that but of the people who…that it predicts to develop dementia it will be enriched to people who it’s due to Alzheimer’s disease (P-Ex2)  from an educational standpoint, um, that this comparisons is useful for people to know about because I do think there’s a misconception that amyloid PET tracks the disease. I think what we would find if you looked at the literature is that amongst um, Alzheimer’s disease patients markers of neuro degeneration better predict change overtime than amyloid does (P-Ex2) |  |
| Implications of test results  ***[See separate section about benefits of having a diagnosis in general]*** | **Quality of life**  Does knowing that prognosis improve or diminish the quality of life for the individual. (P-Ex1)  And does that knowledge um, improve or diminish quality of life. (P-Ex1)  Does the outcome affect the quality of life as that’s sort of incorporating both benefit and harm potentially. (P-Mod) | Which you know IDEAS I think you’re sort of trying to get at although I think they could have designed it much better but you know the ideal study, um, and we can ask this question, is really…does amyloid PET make a difference in terms of outcome quality, anything you want, quality of life, cognitive functioning, preparing for decline and you know so a broad number but it would be a utility test you compare if you take a cohort of patients and they had amyloid testing versus if they didn’t have amyloid testing, what difference does that make 5 years later. That’s the over arching question I think. (C-Method)  **Appropriate limits on independence**  Other harms to think of would be employability uh not just for money but also volunteer. Could someone be taken out of volunteer position because of a test result. Impact on driving, making financial decisions. Does the spouse say Ok we’re going to change your ATM card to a $300 a day limit. (C-GDDI4)  That could be considered a benefit, too (C-Ex1)  Benefit for the family perceived as wrong by the patient perhaps (C-GDDI4)  If you have dementia then the [loss of] driving becomes a benefit… (C-Ex1)  Driving cessation becomes a benefit, employment or financial management cessation becomes a benefit. (C-GDDI4) |

Table 8. Themes and Exemplary Quotes Relating to Time Frames (“T” in PICOT)

| Theme | Gp 1 (Exper, WITH PATIENTS) | Gp 2 (Control, PHYSICIANS ONLY) |
| --- | --- | --- |
| Needing a time frame for PICOT questions where prediction is an outcome | I really hate time frames (P-Ex2)  How about we say 5, 10 (P-Mod)  15 (laugh) (P-CP2)  7.25… (P-Adv)  Yeah, no, no. And I know that answer was going to come up because some people… (P-Ex1)  Although in that case I would probably, for the…at least the prodromal and the symptomatic mention one year time frames as well because yearly does have a, you know meaning and even literature in some cases. (P-Ex1)  The literature for…for uh, at least in prodromal disease like the common numbers are like 1 yr, 3 yr, 5 yr and 10 yr are basically the…what generally is studied, um, you know as a frame work, but (P-Ex2)  **Prediction may not be as good for longer time frames**  Yeah I can imaging something like that in the case here that your prediction for 20 year outcomes is going to be less accurate than your prediction for 5 or 10 year outcomes just based on the type of physiology of the disease. (P-Ex2)  **Longer time frame may be meaningful for young onset patients**  If you’ve got a 30 something year old that for the pre-clinical let’s say, in your clinic, they may want to know 20 years from now am I going to be demented and that may be, that’s still early onset, that may be a meaningful question for them. (P-Ex1) | [Group 2 did not write a time frame into any of their questions, but they had some discussions about time frames]  We only have a 10 year horizon though so someone’s tested at 65 and it’s negative you can say they probably won’t develop Alzheimer’s by 75 but beyond that you really can’t say. (C-GDDI4)  And people put in the timings I mean this is the way you know we came up with the question like that, the timing would be really limited. We would have that, uh, population of people we would have the timing um confidence built into that question. (C-Mod)  Progress to AD would be important. (C-Mod)  And then you need a time in there. (C-GDDI4) |

Table 9. Themes and Exemplary Quotes Relating to Other PICOT Question Decisions

| Theme | Gp 1 (Exper, WITH PATIENTS) | Gp 2 (Control, PHYSICIANS ONLY) |
| --- | --- | --- |
| Assuming that amyloid accurately predicts pathology | So do we need just the first question, does beta amyloid accurately predict amyloid pathology in the brain as like a foundation or can that be in the background of the paper? (P-Mod)  I think in the background. Because it is so self evident and (inaudible) (P-Ex1)  So we can do it as a background? (P-Ex2)  I think that’s fine (P-Mod)  Yeah I mean I think it’s certainly useful background information because I think people don’t know sensitivity and specificity of these different measures, but I don’t necessarily think…I mean it was approved on the basis of performance. (P-Ex2) | Is it a given that the scan detects amyloid? I mean is that something that we need to look at again. Because everything that you guys have set, you say if that scan doesn’t show amyloid they don’t have Alzheimer’s pathology, they don’t Alzheimer’s. You know they don’t have amyloid in their brain. That’s already given that we need to just state that or should we look at it again? (C-GDDI1)  I think we should prove it (C-Method)  I think what the given is they see it, it’s there right? It’s not because its really binding to something else? (C-GDDI-Ex2) |

Table 10. Themes and Exemplary Quotes Relating to **Harms** of Amyloid Testing

| Theme | Gp 1 (Exper, WITH PATIENTS) | Gp 2 (Control, PHYSICIANS ONLY) |
| --- | --- | --- |
| Categories of harm |  | So we have the direct harms of the test, we have the diagnostic accuracy of harms, the false positive, false negative. And then we have the you know more indirect harms if you’re false positive, false negative you can do all these things in appropriately. So that’s all of them. (C-Method) |
| Harms may be different by population tested |  | I think that’s…and it changes depending on what your population is. So if you’re asymptomatic those are very relevant but if you have dementia then the driving becomes a benefit, you’re not… (C-Ex1)  Driving cessation becomes a benefit, employment or financial management cessation becomes a benefit. (C-GDDI4) |
| Direct harms of testing |  | **Radiation exposure**  What’s the radiation exposure of an amyloid PET scan compared to say a CT scan?  I know that we only allow our research participants to have one every six months…We don’t encourage it but if they…for research purposes if they’ve had a full body PET scan for some other investigation for malignancy in the past six months we would exclude them from their scheduled PET scan. We do PET scans once every three years in our cohort but that’s not to do with radiation exposure. (C-GDDI-Ex1)  And there will be information on that, I just don’t…I don’t know if we get that from a radiologist and we can compare it to a CT brain to put it in reference for them. (C-GDDI-Ex1)  What about radiation up there (C-GDDI-Ex1)  Yes… Radiation… Those are all important direct harms (C-Method)  **Allergic reaction**  Are there rare allergic reactions to the isotopes? (C-Method)  I think there are some rare… QT prolongations for amyloid (C-GDDI-Ex1)  For tau (C-Ex1)  For tau, so not amyloid PET necessarily but I’m sure there’s I’m sure there’s an FDA list (C-GDDI-Ex1) |
| Harms associated with positive test results | **Worsened quality of life as potential harm**  You know I always say forewarned is forearmed, but when I went into fellowship with [my mentor], you know one of his statements that he made often times in counseling was that sometimes foreknowledge is fore-sorrow and that gets to, you know, is sometimes knowing either you’re at risk or knowing what you have more harmful to persons…to a person’s quality of life than helpful (P-Ex1)  We had a patient who had mild cognitive impairment and had a CSF study and it was amyloid positive and she became severely depressed and actually cognitively she was incredibly steady for a number of years afterwards but functionally and in just her sort of quality of life had diminished significantly (P-Ex2)  **Increased worry/constantly thinking that symptoms are AD-related after possible test**  Some patients don’t want to know the results of these tests because of their psychological reasons that knowing that they have amyloid in the brain or that their likelihood that they’re going to develop dementia is much higher if they’re for example mild cognitive impairment that um, depending on your sort of personality, you’re psychological makeup, um, some people want to know as much as they can, other people would rather say let’s let time take its course and not have to…everytime I forget my coffee then that’s Oh my God my Alzheimer’s is coming on now or something. (P-Ex2)  That’s for sure (P-GDDI-Ex1)  **Depression as potential harm**  Certainly in terms of harm you could have a diagnosis of depression. Somebody could get that diagnosis and go the other way, not take the proactive approach. (P-Adv)  We had a patient who had mild cognitive impairment and had a CSF study and it was amyloid positive and she became severely depressed. (P-Ex2)  **Suicide risk as potential harm**  I’ve had some people say if I’m going to get Alzheimer’s I’m just going to kill myself. (P-Ex1)  Oh I’ve heard that many times. Just take me down to the river. (P-CP2)  Now I’ve yet to see anyone act on that but I’ve heard it. (P-Ex1)  We’ve had some who have acted on that (P-Ex2)  There you go (P-Ex1)  There’s a few recently that had hit the… (P-Adv)  **Discrimination based on test results**  What about discrimination? I mean I know we’ve got genetic non-discrimination laws on the book but if you are pre-clinical and you get this is it now pre-existing condition? All those are supposed to go away under (inaudible) as well so maybe that’s less of an issue now. (P-Ex1)  **Losing access to insurance**  With ApOE there’s a literature base on this… with APoE actually I believe there was…I think in both cognitively normal and in patients there were not a lot of negative effects and actually the biggest change was in people getting long term insurance or additional insurance. (P-Ex2)  Thinking about long term care, if you have a pre-clinical, can long term care go back to that and look at that and say well you have a pre-clinical that’s why you signed up and now we’re going to reject you because of that, because you know P-P1 and I looked into long term care. He already had a pre MCI or the MCI by that time and I deliberately asked the question, he said Oh yeah you can still get it. You go back to your thing, you’re just telling me that, you go back, look and ask and he called me back he said No you are not eligible. (P-CP1)  The potential pitfall is it might prove a barrier to accessing those advance plans (P-CP1)  For example I can see it impacting your ability to get life insurance or increase your life insurance (P-Ex1)  **Not telling employer about results and then losing job without ability to get disability**  But if you choose to not diagnose…or if you choose to not disclose though and so in an employment situation for example and you start to have some challenges and end up loosing your job, but you didn’t tell him that there was an issue, now there’s no protection. So I mean that could certainly be something and we have had clients that have been in that situation where they did not share that information and there was a change in their ability to work and the employer didn’t know, they lost their job to the point earlier, about yeah this was a younger person, kids at home carrying benefits, I mean there you’ve given to that whole…because with that younger onset that’s a whole other animal versus that 80 something. (P-Adv)  *No other discussion about empoloyment/volunteer in P group; maybe because lay participants older?* | **Worsened quality of life as potential harm**  Quality of life (C-GDDI-Ex1)  **Anxiety and depression as potential harms**  And then the psychosocial issues of anxiety, depression, all that (talk over) (C-GDDI-3)  **Discrimination based on test results**  The genetic non discrimination act is only applicable to genetic testing, it does…and even that is not uniformly followed. It is supposed to be but it’s not. (C-Mod)  It hasn’t been tested (C-GDDI4)  It does not apply to this kind of testing (C-Mod)  **Losing access to insurance**  It would be interesting I think to know how they use it for planning I mean does that include buying long term insurance. (C-Mod)  Yeah it does, they can’t… (talk over) No, no. I looked at long term care insurance about 15 years ago and the first question was in the last 5 years have you seen a health provider for the possible diagnosis of Alzheimer’s, MS, Parkinson’s Disease. Not that you’ve been diagnosed with the possible diagnosis that kicked you out of the system. (C-GDDI4)  Yeah then you really wonder then about the implications of amyloid testing and a possible diagnosis on those issues. (C-Mod)  So this is a harm (C-Method)  It’s a harm, yeah (C-Ex1)  **Loss of employment and volunteer opportunities**  Other harms to think of would be employability uh not just for money but also volunteer. Could someone be taken out of volunteer position because of a test result. (C-GDDI4)  So what are your harms in terms of your outcomes, what can… what bad stuff can happen because you did or didn’t do the test? (C-Mod)  Well domains would be like work, be it pay or volunteer… (C-GDDI4) |
| Positive test won’t tell you if/when you’ll get symptoms | I think there’s um, sort of equipoise about whether…I don’t think, I don’t know if it’s clear that there are people that have amyloid who would not get Alzheimer’s disease if they life long enough to get the disease. (P-Ex2)  I’m going to disagree with that statement. I think we’ve got enough from the religious order study to say there are some people. Unless you’re talking about in their hundreds. (P-Ex1)  I’m not suggesting that it’s unknown answer. I’m just saying that there are plenty of people who die with evidence of Alzheimer’s pathology in their brain. The question is would they have developed symptoms if they lived long enough and we don’t know in an autopsy based study how long they’ve had amyloid for and to what degree they’ve had associated memory deterioration with it. I think it’s unknown. I mean this is why longitudinally having a marker like this in following someone, are there people…and maybe there are, who have amyloid for 30 years. We have patients in our cohort that I’ve got CSF studies in the 90s that haven’t yet developed cognitive impairment with evidence of amyloid. Now maybe that CSF test that we did wasn’t, you know accurate or whatever, but um, I think it’s unknown. You know and there probably are modulators that are like who’s more likely to develop (P-Ex2)  While amyloid in the brain probably puts you in a high risk category for having Alzheimer’s, it is not destiny. (P-Ex1)  And it’s only if you’re going to get worse and then the answer is maybe, you know if definitely will, some of the people with pre-clinical amyloid it puts you in a higher risk for developing Alzheimer’s disease, but not necessarily destiny. It teslls you nothing about your risk of developing other cognitive impairments. (P-Ex1) | A positive scan in that age group does not remove the clinician’s responsibility to investigate absolutely every alternative. You can’t hang your hat on that and say Oh this is classic AD. (C-GDDI-Ex1) |
| Harms associated accessing test | **Lack of accessibility to test as potential harm**  I also wonder if under harm and I don’t know maybe it doesn’t live here, but accessibility is a potential harm. Talking about…because the scenario you just went through makes me think looking at some of the chapters of the association and areas where there is not the ability of a potential patient to access any of this. I mean they’re lucky if they have a primary care physician let alone a neurologist and they man have to travel miles which may or may not be possible, they could be alone, they may not have a primary caregiver. So you know if we make…if we recommend this as a guideline, but PS here’s where you have to live to be able to… (P-Adv) | Cost burden, inconvenience (C-Method)  **Need for pre-test counseling (which people may or may not get)**  Do you give any pretest counseling or is it necessary? (C-Mod)  Yes (C-Ex1)  I guess it’s clinic-dependent, right, it’s not (inaudible) (C-GDDI-Ex1)  So should we be talking about it? (C-Mod)  I think it’s in the harms (C-GDDI2)  It’s part of the harms, yeah (C-Ex1) |
| Risk of getting test results automatically rather than from doctor | And also now in our day and age of where verything gets returned to the patients before the doctor sees it, you know there’s definite psychological [risk]. But you know that also, those are things that if it became more accessible maybe there’d be better education… (P-Ex2) |  |
| Overuse of test/ inappropriate ordering |  | **Inappropriate and overuse as harms**  So then I guess one of the issues that comes up within the scenario is uh I don’t want a situation where um, a confused 70 yr old is admitted to my service and before I go in the next morning, the residence have ordered amyloid PET, you know, it’s overuse. (P-Mod)  No the emergency room will do it (C-GDDI4)  You know you’d be surprised at what happens. You know it will be for those patients that they will get it done from my outpatient clinic I’ll be fighting for someone and it won’t get done. But, no. So what I mean I was just being factious… but overuse. (C-Mod)  I think that’s why the…certain people ordering it in the right context (talk over) (C-Ex1)  I’m going to put overuse in the harms page (C-Mod)  Anecdotally I’ve seen 10 physicians who have buddies order a PET scan for this, PET was abnormal and they are cognitively normal. It’s one of the hardest cases you can imagine, practicing physicians. (C-Ex1)  There’s harm being done now like I said I’ve seen these cognitively normal physicians who have gotten an amyloid PET from a friend you know that’s an issue.(C-Ex1)  **Could over testing find people where amyloid/AD was never going to impact them clinically**  Do you talk about the concept of over diagnoses like they do in cancer? Um for example in cancer uh there are a certain percentage of women that are screened and pathologically confirmed to have cancer but it…they know from the numbers that that cancer was never destined to impact them clinically. (C-Method)  I think if you…we take this people who have…because…it’s a good question. I think you start with people who have objective cognitive impairment and so there is enough objective uh, clinical change in that person that is brought to a clinic. Uh if you start scanning people who have no symptoms then you could run into that issue where you have pathologic AD in the brain but… That’s really important so in terms of the subjective group, um, and that was why the appropriate use criteria excluded them because it’s a mixed bag and it looks like people who have subjective cognitive impairment are at increased risk of developing MCI and dementia later compared to people who don’t but it’s not a certainty and so um, the appropriate use criteria excluded them because of that. (C-Ex1)  **Repeating test inappropriately**  Epeat imaging could be a harm (C-GDDI4)  Or prevention of repeat imaging would be a benefit (C-Ex1)  Right. So someone says Ok, well it looked but we’re going to repeat it in a year or someone who’s not understanding the test. (C-GDDI4) |
| Societal resource harm | [A] harm I think of…of amyloid imaging we have to think about is just a resource harm, societal resource, you know what is the cost of scanning in terms of what recommendations we make, um how…you know what are the sort of economics of it as well which I think is again another part of the question to answer. (P-Ex2)  So it’s a potential population either depending on how we frame it could be large and economic costs could be large as a result of ordering this on everybody over 70, you know. (P-Mod) | It’ll be population or you know societal or those things so that’s here (C-Mod)  So costs are (C-Ex1)  Overuse or resource use I guess (C-Mod)  Well cost (C-Method)  Can resource sort of be a whole umbrella of costs, personnel, whatever (C-Mod)  Diverting funds to (C-Ex1)  And so that’s harms at the patient level. Do you want to look at over use as a harm at the patient level or more of a societal level. (C-GDDI4) |
| Over-reading test findings | I do think harm is…I think that the harm…I mean I…it’s…you know I even get…even radiologists are reading MRI scans as atrophy… I have had consults just for…and someone who’s totally normal who got the MRI because of a headache or something you know and so um, there is a risk to it (P-Ex2) | For whatever reason a lot of SPECT scans are done at our institution and I’ve never seen a normal SPECT scan and people are you know walking around with these diagnoses that are fine. (C-Method)  We went away from SPECT for that reason because I think the rest of the people said there’s never been a normal one so we had to stop doing that. But yeah so we stopped. (C-Ex1) |
| Incorrect interpretation of meaning of test results | **Incorrect interpretation because (a) it’s not destiny and (b) interpretation depends on age of patient**  I think the big harm I was concerned about and most of my colleagues in terms of it being ordered just on…by non-cognitive experts whatever you want to describe that is the interpretation of it because um, basically depending on … we know from um, some very good studies that while amyloid in the brain probably puts you in a high risk category for having Alzheimer’s, it is not destiny. There are plenty of people who get out of this life with brains chucked full of amyloid who never get even memory problems let alone full blown Alzheimer’s dementia. Um, so it’s risk but not absolute. So that’s one problem, um and the other you know, is basically um, Ok so you’ve got…it now…I will say this much, in early onset it becomes much more sig…you know powerful because young folks should not have a huge amount of amyloid in their brain. Older folks you know it starts getting messy. So I think that was one of the big concerns with the amyloid imaging. (P-Ex1) | **Incorrect interpretation because people make actually have other primary dementing diseases even if they have a positive amyloid scan**  One of the issues with amyloid imaging is you know it can’t be substituted for the history because the DLB second most common cause of dementia, 60% will be positive on amyloid imaging so if you’re…you can’t answer the question is the AD or DLB because it’s going to be positive. (C-Ex1)  Because the prevalence rate is so high of Alzheimer’s disease neuropathological change in the brains of people over the age of 80, you could have somebody walking to your clinic with Huntington’s who would have a positive amyloid scan. I mean do they have Alzheimer’s disease, possibly, do they have Alzheimer’s dementia almost certainly not, they have Huntington’s. (C-GDDI-Ex1) |
| Test error – wrong results (false positive, false negative) | So it also sort of brings up the potential harm, misdiagnosis or misprognosis if you want to talk about the pre-clinical, the potential harm in that gets to well just how good a test is it. (P-Ex1)  There are no diagnostic test that are 100% and you recognize that. (P-GDDI-Ex1) | Misdiagnosis or false positive diagnosis, right. And then because all of these you can actually put them to domains in the categories of either misdiagnosis or false positive diagnosis. So really the major harms of this is I missed it or I said he had dementia when he didn’t. (C-Mod)  Of course in missing it, it may just be not Alzheimer’s dementia. (C-GDDI4)  Sure, but we are considering here the harm of actually missing it, true missing. (C-Mod)  I think those are two harms that we should list. False positive, false negative. (C-Method)  I say having the false positives and false negatives actually isn’t a harm in a way…well…yes false negatives those are harms (C-GDDI3)  **False positive result as harm**  No if they know but they know incorrectly it’s a harm. They’re told that they have Alzheimer’s disease and they don’t have it, that’s a harm. (C-Method)  If it’s a false positive you shouldn’t be on early treatment so all of these things are, as you pointed out, are harms or benefits based on whether it’s a false positive (C-Method) |
| Appropriately negative test giving false reassurance | So if you’re pre-clinical and…a positive amyloid scan will tell you you are at increased risk for developing Alzheimer’s disease and probably sooner rather than later compared to someone with a negative amyloid scan. The person with the negative amyloid scan is not out of the woods. They live long enough they might turn positive. (P-Ex1)  So pre-clinical if you get a negative amyloid scan it does not necessarily tell you your risk of all cause dementia, Ok, and it still doesn’t 100% get you out of the woods for someday developing Alzheimer’s, again it depends on the timing, you know if you’re 30 something, you’ve got a negative scan that don’t mean like when you’re 70 you ain’t getting Alzheimer’s. Um, in terms of the prodromal MCI, if you have a negative scan it doesn’t promise that you will never go on to dementia because there are other causes there, but it just makes it much less likely that it will be that Alzheimer’s flavor. And again, if you have a dementia and you have a negative scan it doesn’t mean you don’t have a dementia it just means it’s probably something other than Alzheimer’s. (P-Ex1) |  |

Table 11. Themes and Exemplary Quotes Relating to Converting PICOT Questions to **Plain Language**

**Group 2 forgot to do the plain language question summaries**

| Theme | Gp 1 (Exper, WITH PATIENTS) |
| --- | --- |
| Patients are people/persons | All right. So we can replace patients with people. (P-Ex1)  Persons (P-CP1)  Persons? You like persons better? (P-Mod)  I think people is understandable (P-Ex1)  Group with patients does not use term "patient" as much as other group; can do stats on this |
| Prediction terminology | Does amyloid imaging…do you want to get rid of accurately just say predict? (P-Mod)  Just predict (P-Adv)  Help predict (P-Ex1)  Worsening of memory (P-Ex2)  Help predict getting (P-Ex1)  I like the help (P-Adv)  Help predict? You think that’s helpful? P-Mod  Yes because if you say predict they are going to think it’s the end all, be all (P-Ex1)  How about improve prediction? (P-P1)  Help (P-Ex1)  Plain language (P-CP2)  Help predict (P-Mod)  Getting (P-Ex1)  You know for plain for like you know somebody who’s (P-CP2)  Not all of us have a PhD (P-Adv, to P-P1)  Getting is very plain English (P-CP2) |
| Do people understand term risk | So you think “at risk is meaningful? P-Mod  I think people understand the term risk (P-Ex1) |
| How to describe amyloid imaging | Do we need to translate beta amyloid imaging? (P-Ex1)  A picture of the brain that looks at the protein (P-Ex1)  I wonder if we should just define amyloid imaging at the beginning of the question (MJA)  I think so (P-CP1)  As a consumer, most of us have heard the word PET scan. Maybe not everyone, but you know beta amyloid, you know and use it with PET scan as maybe a further definition of it because it is a type of a PET scan. (P-CP2)  So what’s the lay definition? (P-Mod)  A special picture (P-Ex1)  A specialized… (P-Adv)  I don’t know that sounds awfully fancy to me (P-Ex1)  Can we have one fancy name? (P-Adv)  All right… picture of the brain that looks for a protein, now do we want to replace protein with something? (P-Ex1)  Yes. (P-CP2)  With what? (P-Ex1)  What is a protein? Is it a… (P-CP2)  What about a marker or a signature (P-Method)  No not signature (P-Ex1)  Compound or chemical or (P-Mod)  A substance (P-Ex1)  I think that’s a good term. A substance. (P-Mod)  They are actually referred to as biomolecules (P-CP2, looking at phone)  Put that away, P-CP2. Put it away. (P-Adv)  Look I’ve got some people who come to my clinic, they like have a 6th grade education, you know. (P-Ex1)  Yes I know (P-CP2)  I’m trying to keep it as plain spoken as possible (P-Ex1)  I’ve never heard of that one, actually. I was just… (P-CP2) |
| How to describe terminology used to reference different cognitive populations | **At risk/asymptomatic**  Ok, so at risk for Alzheimer’s disease who have, now this is where we get to translate a little bit (P-Mod)  Without memory and thinking problems (P-Ex1)  **MCI**  With mild memory and thinking problems? (P-Mod)  Sure (P-Adv)  Do we want to put in parentheses “mild cognitive impairment” because some people would have heard that term? (P-Ex1)  I’ll say as a Cochrane Collaborator that would never go… The mild cognitive impairment part. Just that would never go. (P-Method)  This is for the patients, though. (P-GDDI-Ex1)  This is plain language (P-Ex1)  That’s what I’m talking about, the plain language how about… You could say “sometimes called mild cognitive impairment” (P-Method)  **Dementia**  Ok in patients we’re leaving dementia as a word. (P-Mod)  You need to define dementia (P-GDDI-Ex1)  I would say people have a lot of trouble differentiating dementia and Alzheimer’s (P-Ex2)  Yeah they do (P-CP2) |
| Worsening-progressing terminology | It’s sort of worsening though I think this… (P-Ex2)  Progressing, um (P-CP2)  Getting demented due to Alzheimer’s (P-Ex1)  Getting worse, I guess, I mean I would say worsening and developing (P-Ex2)  Predict worsening. Is that OK with you guys? (P-Mod)  Predict worsening… (P-CP2)  I like putting that dementia word in there, but that’s OK (P-Ex1)  Declining cognition (P-P1)  No. I’m sorry, sir, you are not a plain spoken man. (P-Ex1)  Anyway he’s coming up with too many big words (P-CP1)  Worsening mild memory and thinking problems (P-GDDI-Ex1)  Memory and thinking problems (P-Mod)  You know just use the same language (P-GDDI-Ex1)  Well if you just say mild worsening… you’ve… so I think that is not actually… I think you’re talking too low now (P-Ex1)  So can we say dementia? (P-Mod)  Yes. Worsening to dementia. (P-Ex1)  Speed, predict speed of worsening (P-Ex1)  I don’t like that (P-CP1)  What would you like? (P-Ex1)  I think most people understand rate of decline (P-CP1)  It’s a little sophisticated (P-CP2)  Should be what, a 6^th^ grade reading level? (P-GDDI-Ex1)  Speed of worsening. A 6^th^ grader knows what speed is, they know what worse is (P-Ex1)  Help predict speed of worsening (P-Ex1)  That’s too many words (P-Adv)  Well rather than saying…can we look at worsening? Worsening due to Alzheimer’s could we say does this change in people with mild memory and thinking problems? No wait a minute it’s number 7. In people with dementia does amyloid PET imaging help predict Alzheimer’s disease becoming worse. Is it possible to say that? (P-CP2)  Alzheimer’s disease… (P-Mod)  Becoming worse (P-CP2)  It’s the speed of decline (P-P1)  Speed (P-Adv)  Yeah we know it’s going to get worse so it’s really the speed. It’s not if, it’s when (P-Ex1)  It could, how about “the speed,” how about “the speed”? (P-CP1)  I’m good with putting “the” in (P-Ex1)  What is worsening? (P-P1)  It’s in the dictionary here (P-CP2)  But I mean is it memory? I mean, worsening is an adjective that applies (P-P1)  It’s very broad I agree and (P-Mod)  I think we have to keep it broad (P-Ex1)  Yeah I was going to say I think we want to kind of leave things relatively like…I think you’re right that it’s an important question for sure but again we wanted to make these sort of interpretable to anybody who’d be reading it and they can take it where they want to for what (P-Mod)  Did you say worsening symptoms or not symptoms because symptoms is improper at this time (P-CP1)  What is worsening (P-P1)  Yeah, what is worsening? (P-CP1)  Worsening problems? Do you want problems in there? (P-Ex1)  To get rid of speed and say worsening symptoms? (P-Mod)  No, it’s speed of worsening (P-P1)  It’s worsening blank (P-Adv)  What is worsening? (P-P1)  The problems, you’re saying? (P-CP1)  But the thing is there’s so many things that can worsen with dementia. I think you should just leave it at worsening. And I think people will get what that means in general. (P-Ex1)  Worsen is actually a verb (P-CP2) |

Table 12. Themes and Exemplary Quotes Relating to Guideline Methodology Issues Other than PICOT Questions

| Theme | Gp 1 (Exper, WITH PATIENTS) | Gp 2 (Control, PHYSICIANS ONLY) |
| --- | --- | --- |
| Is scope correct | While we’re again speaking globally at this point, our framework is beta amyloid imaging diagnosis dementia I guess I raise the question is that the right framework? Uh two things, one is it diagnosis in dementia or is it in people with you know other cognitive things where there’s a worry about dementia, is it the right framing. The other is, is it really fair to just take beta amyloid imaging in isolation when there’s all these other tests that one can do in the situation of at risk or incipient dementia. And I just…now you tell us what our frame is and we’ll go with it. (P-GDDI-Ex1)  Well there’s a different guideline on the waiting list for diagnosis of dementia and I think that’s more than we can probably take on with this guideline. I think that’s a guideline that needs to be done. Our last guideline on that topic is like unfathomably out of date. Um but that is a bigger…that’s a big project um, that’s a much bigger project than people here agree to. I think that’s going to be a many year project just because it’s getting so complicated now. Um, so I…you can decide how to interpret the topic of beta amyloid imaging in dementia but I would say we shouldn’t broaden it to diagnosis of dementia because that is on the waiting list as a separate topic and would be a very large endeavor which we want to undertake and then I think what will happen when we undertake that one, um is that this will probably be referenced as part of that guideline. (MJA)  So you don’t have to…so you can feel free, like the diagnosis of dementia was just kind of the framing but you could say what you might decide is prediction of what’s going to happen in 5 years is totally fine. Uh the word dementia was used was because they didn’t…when we were requested to do this topic they didn’t necessarily say we had to focus on Alzheimer’s Disease and so if you as a group feel that we want to look at dementia more broadly, we want to look at dementia with Lewy Bodies in amyloid, are we looking at dementia in general, are we focusing on Alzheimer’s? Those are things that haven’t been decided. So I think that the topic that you should ponder is beta amyloid imaging and its clinical use in the memory realm rather than the vascular realm, um, but how we define those questions you can be flexible and pick the important things. (MJA)  So could the title even change on this (P-Ex2)  Oh absolutely (MJA) |  |
| Limiting guideline scope for feasibility | The one thing I would worry about in the guideline setting is do we then need to do systematic reviews for every single one of those alternative tests and their prognostic capabilities that adds you know ten fold potentially to the amount of systematic reviewing and (talk over) (P-Mod)  So having done guidelines for a number of years now it’s always tempting to try to get more questions and broaden things but the answer should be go narrower because getting it done… I’m glad you brought it up because it’s always that inclination and I feel it too, but the experience is that you want to try to stay focused and…and be narrow so you get it done, obviously good as the uh…or prefect including everything is the enemy of actually getting a good product that’s actually finished. (P-Mod)  Well it’s…no I’m agreeing with you I think that if you add the additional question, the third question, you’re adding…at the risk of increasing the workload by 50% without necessarily increasing the workable outcomes by 50%. (P-Method) |  |
| Audience for the guideline | Just bring this back, so one of the major purposes for these guidelines is to inform the general neurology membership of the Academy and so we do have to kind of remember that our primary consumer is general neurologists deciding what to do with this…What do I do with this test. And so when we look again at our PICO things we should make sure that at least somewhat we’ve…we’re trying to help the general neurologist do the right thing. So just to remind us about that because I think we’ll probably need to come back to that six or so that we’ve developed and say do these still fit, do these work and come to some resolution. (P-GDDI-Ex1)  Can I just say though that patients generally interact with their general practice and how do we get this back to that stage because that’s where I think, you know I was fortunate that my general practice doctor listened to us and you know asked the questions and then got us into looking at this seriously long before I would ever have thought about it and I think my life is extremely improved by having that early diagnosis. And so I mean that’s where I think you back that back to how can we get to that earlier diagnosis or just channeling say this is something to check up. (P-P1)  Just sort of reminding us that our actual designated primary audience, but we have a dissemination program as part of these guidelines and early on I don’t know whatever you started for this one, but very early we started saying Hey we need to tell the family doctors, internists as well as the neurologist so we would have a system for that. (P-GDDI-Ex1)  Well because even if we could get the family doctor to more readily refer to the neurologists, you know if the family doctor is not the one that’s actually doing this but we need to connect them to neurology I think sometimes they are in a little bit of a box even if they are in the same building we’ve had experiences with clinics where the neurology department doesn’t talk to primary care and they are in the same hall. You know so I think even if this starts to establish some communication in that process that will improve the diagnostic process and it will improve outcome. (P-Adv)  ***Guideline subcommittee member focuses on membership as intended audience but patient, advocate discuss importance of primary care as an additional target – this holistic approach seems very patient-centered and is raised by the non-physicians on the panel*** |  |
| Questions not based on available data | I would like to remind you… the questions we’re going to ask are not based on the available data. The questions you ask are based on the importance of the questions... (P-Method)  I agree with P-Method very much and I appreciate the point that we don’t want to think about what’s available right now in the literature to frame all the questions (P-Mod)  And let me remind you that we’re creating question not based on the evidence. We are creating questions that have the utility to be useful, that we want answers to. (P-Method)  I guess my only…and again I’m…this is, um, I’m differing to y’all in terms of not worrying about what the knowledge base is out there but our answer is going to be, we’re not going to be able to answer three of the questions which are outcomes and quality of life because those are things that are actively trying to be assessed right now. And so that’s fine, again we can sort of make a statement that there isn’t an evidence base for that but that is going to be the answer. (P-Ex2)  So for this role what we are really framing the conversation, we are saying these are the right questions you should be asking before considering clinical implementation and if the evidence supports or refutes on of these or more, great. If it doesn’t, these are where you need more evidence. (P-Ex1) | But as far as the guideline is concerned remember we can ask through research related questions or sort of questions that are related to the clinical arena. So it doesn’t preclude the fact that you know because there’s no treatment it doesn’t preclude the fact that you cannot ask that question. (C-Mod)  Right that is…and everything else is sort of, you take that over arching questions and decompose it into its parts and what are those important parts because even though we think we won’t find evidence to answer the over arching question if we find good evidence for the parts, all the steps that are necessary then we might…that will help inform it. (C-Method) |
| Risk of incorporation bias in questions |  | But most of these insist…I mean it seems like there would be some type of incorporation bias in a lot of these questions. (C-GDDI1)  Yes and we’ll have to look for that because we’ll have to make sure…I mean that that’ll be and they will be class 4 but we’ll have to make sure that you know the standard eval with diagnostic accuracy or the standard eval was done total without knowledge of the amyloid PET and if we can’t be certain of that you know the study will be… (C-Method) |
| Designing systematic review |  | So this will be a diagnostic pragmatic search? (C-Mod)  Uh I don’t think so… because of diagnostic filters isn’t that validated… We’ll miss something. I don’t think we can. If it was a therapeutic question then you can do it for this I suppose. So this is…uses the therapeutic scheme. These two use the diagnostic accuracy scheme, this one would use the screening scheme. (C-Method) |
| Challenge of low-quality evidence for diagnostic questions |  | The usual issue that we come up with is that the numbers look pretty reasonable you know the sensitivity and specificity is predictive values look pretty reasonable but we have very little confidence in the numbers because of the study design. And then we struggled with that and then we made, you know we may No…like level C, You may do this you may choose but then you better tell your patients that there’s a lot of uncertainty in the results which is always hard particularly with diagnostic tests because if there’s a lot of uncertainty the diagnostic test isn’t telling you what you want, it’s not that helpful. (C-Method) |
| Recommendation process | **Recommendations are not based on evidence alone**  The evidence means the starting point of the recommendations and there are some of our guidelines that have had pretty weak evidence but a lot of pretty strong recommendations because the other elements pushed it that way. (P-Method)  Underlying principles, the related evidence (P-Mod)  So we don’t want to already have us in a fallout even if the evidence is week (P-Method)  **Judgement is involved in drafting recommendations**  When it comes to recommendations there is absolutely, there’s judgment you know and everybody on the panel gets to help with that framing of the recommendation and so that’s important. (P-Mod)  **Counseling recommendations during recommendation phase of guideline**  So one of the things on the recommendation phase is that we can have discussions about the sort of nuanced counseling things that we can incorporate that into that phase (P-GDDI-Ex1)  That’s very important (P-CP2)  **Feasibility/availability considered during recommendation phase**  [When it] comes into the recommendation phase we could look into availability of a test or treatment and then that downgrades the level of recommendation it if’s not readily available. So there’s some of that, when we get to that phase that will be a critical piece. (P-GDDI-Ex1)  **Option of a Level R recommendation**  There is a specific recommendation which is…there are different levels in the end come out like you should, you must, you can, there’s one that’s just level R says it can only be used, we recommend that it’s only used in a research setting. So that’s perfectly Ok. (P-Method) | **Recommendations involve consensus but are not purely consensus-based**  Coming back to [the appropriate use criteria]…the methodology was very different that the methodology that we used. It was not based on the evidence based systematic review and the consensus process so we do… we do have a consensus judgment for the group at the end of the systematic review, but uh so that was a little bit different. (C-Mod)  **Related evidence is involved in drafting recommendations**  Right why not to get it. So now we’re connecting the dots and so we connected the diagnostic dots, what about all these other outcomes, Ok. So all these other outcomes are what is the advantage of knowing that you’ve got Alzheimer’s disease. What is the advantage if you’re not certain of looking for alternative causes or the harms, what are the advantages of early treatment, what are the advantages of the anticipating preparing for Alzheimer’s disease, what are the advantages of dealing with these social consequences, Ok. These questions are not specific to amyloid PET, Ok. These questions are specific to whether you have dementia or you have Alzheimer’s disease. So this is what we would call related evidence. And so…there’s actually one more question, don’t let me forget. So the way we will deal with this is because we’re not going to do a systematic search, I wouldn’t recommend looking for uh you know what’s the benefit of knowing that you’ve got a disease or Alzheimer’s disease specifically or all these other things. We’re not, we don’t want to do a systematic review on the efficacy of cholinesterase inhibitors in patients with AD or patients with MCI. You know we don’t…but we may want to use that evidence in the recommendations to connect the dots. Let’s say we find out that it’s highly accurate in symptomatic patient population and we think you should do it and because there…we’re going to want to highlight what all those benefits are and so we’re going to want to find related evidence but we don’t do that systematically. You’ve got to find some good references but we don’t have to do a systematic review of all that. So then all these things which are important outcomes become picked up in the recommendation phase that related evidence which really simplifies the process. (C-Method)  Well this isn’t directly related but just when you talked about the related evidence because this came up for the DMT recommendations, related because we’re supposed to be thinking about related evidence as we go through some of the article reviews and stuff like that. It’d have to be certain classes to be related evidence? (C-GDDI-Ex2)  Yes and no. So the methodologist got together and we wrote sort of compromises. It depends on what the recommendation is and it’s in the updated [methodology manual]… the short version of it is if you’re making a direct treatment recommendation because you couldn’t find good therapeutic evidence for the patients that were pertinent to your question, but in this related population, like children with MTBI versus adult MTBI you found strong evidence you could make a recommendation for children, that requires class 1 or class 2. But beyond that if you’re just rounding out your recommendation or your making a counseling recommendation and you’re suggesting that you should say it this way rather than that way, that evidence doesn’t need to be class 1 or class 2. (C-Method)  **Principles of care involved in drafting recommendations**  I think we could state an axiom so when we get to the recommendation process there’s ways that we can uh, bring in like even principals of care that you physicians will be less likely to look for other causes if they have a positive amyloid PET, you know that may or may not be appropriate but I think we can just state that as a fact that would stipulate that as a principal. (C-Method) |
| Systematic review updated before final publication | In terms of process, so usually what we do is at the end of the process, particularly if there’s a significant amount of time we will update a systematic review before the final phase so if there is new information that may come along we may pick it up at that point but part of what we need to do in the questions we’d say we want a question on economic impact which would then pull in something like that. If we don’t have something on that it wouldn’t come out in systematic reviews. (P-GDDI-Ex1) |  |
| Costs in guideline process are generally patient-level | There’s a methodologic point that we should all be aware of, it’s about the cost. So when we think of cost generally speaking our perspective is that of the individual patient, Ok. The studies that exist about the costs are about the cost to society and so if you’re going to frame questions about cost you may wish to consider specific questions both from a patient perspective and who our patient…questions about cost from society perspective, it may be useful to separate those two. In terms of your thinking. (P-Method) |  |
| Implementation planning | So I’m going to throw out a little methodology thing just to in terms…y’all said it’s important that we implement it broadly by people. When we think about implementation the way that we do guidelines, it’s either narrow or wide in general and so it seems to me what y’all are saying is that we want wide interpretation so that a lot of people get it. Um, we do that when the…we feel like the Academy should um, put significant resources into drafting statements and sending things to newspapers and things like that. (P-Method)  If knowledge of the guideline, yes it would be broad, wide. (P-Ex1) | **Products to accompany guideline**  And that [a pretest counseling document] would be a great shared decision making tool. Not a pamphlet but homework, they have to put in their values and concerns, write down questions they want to ask, where you’re going to get those answers. (C-GDDI4) |

Table 13. Themes and Exemplary Quotes Relating to Background Discussions of Topic

| Theme | Gp 1 (Exper, WITH PATIENTS) | Gp 2 (Control, PHYSICIANS ONLY) |
| --- | --- | --- |
| Being careful about terminology/vocabulary | I think we should be careful about the terms we use because it doesn’t necessarily mean they are going to progress to Alzheimer’s Disease (talk over) (P-Ex2)  So my, but I guess I was also just trying (inaudible) because the terminology that’s being used now in the field is pre-clinical Alzheimer’s disease, prodromal Alzheimer’s disease and dementia level Alzheimer’s and prodromal Alzheimer’s disease is effectively the mild cognitive impairment of Alzheimer’s disease. Pre-clinical is when people have no symptoms of the disease, they are asymptomatic in dementia stage of the disease. And I think we’ve talked a lot about the prodromal it’s not so much the pre-symptomatic. (P-Ex2)  Is it amyloid pathology or Alzheimer’s pathology? (P-Ex1)  It’s like uh…it’s like faith based in terms of people want (talk over) (P-Ex2)  Well we were just discussing whether or not everyone who has amyloid in their brain gets Alzheimer’s so I think it’s important if we’re trying to clarify it and also when you’re talking about comparing CSF to you know amyloid it’s not usually amyloid, CSF to amyloid imaging is the amyloid to Tau ratio versus to amyloid to (P-Ex1)  Yeah and I would get away with, again in what we were just talking about because I’m getting myself confused, but in terms of comparing the different biomarkers (P-Ex2)  Alzheimer’s disease is the pathology, right? (MJA)  So I mean you know I think what you guys are all saying here and I think a lot of this is just that we have so much…the field has evolved so much that we have such difficulty in the terms we use for all these things and I think that that actually creates a lot of confusion in just even how we talk about these things. (P-Ex2)  Yeah. Well I mean it’s kind of impairment due to Alzheimer’s pathology for being straight forward because if…who cares if you have Alzheimer’s pathology if it never causes you a problem during your life, you know. (P-Ex1)  Well it’s talking… he wanted us to talk about dementia (P-Ex1)  What’s the definition (P-CP2)  We need some dice, just roll the dice (P-Adv)  Dementia is just a clinical diagnosis and essentially it’s when people have cognitive impairment that’s enough that it’s impacting their day to day function, what that line is, if you want…you can here us argue about this, you can hear even more mundane arguments of us in a consensus conference whether someone has cross the threshold from mild cognitive impairment where they are not really functionally decline to where they are. So it’s just that it’s somewhat of a judgment call on that regard. But dementia is just where you have enough impairment that it’s really impacting your day to day function. Mild cognitive impairment technically is not really impacting your day to day function, you’re still able to do many of your normal tasks. Now (inaudible) like how do you, well I have memory loss so it takes me longer to do x, y or z and what that…so what that line is, is always a source of opinion. (P-Ex2)  I think terminology is important here so I would make sure that we all agree on the terms we’re using so here it would be diagnosing… So I might say that you’re…you’re diagnosing of non Alzheimer’s disease…you could say Alzheimer’s disease dementia. So in other words refining your dementia diagnosis to dementia due to Alzheimer’s disease. Um, and just try to be kind of consistent with that because it does get super confusing with what we…when we now use the word Alzheimer’s disease and not everyone even in our field is consistent about that. (P-Ex2)  Why are we using the terminology symptomatic dementia? (P-GDDI-Ex1)  Because dementia is symptomatic (P-Mod)  All dementia is symptomatic (P-Ex2)  It was a hangover from symptomatic Alzheimer’s disease (P-Ex1) | The PET scan is detecting we think pathology but it’s the clinical manifestation that we’re interested in as neurologists so that’s why MJA labored over the clarification between Alzheimer’s dementia which would be the prominent amnestic types of complaints of memory loss that’s progressed over the years versus Alzheimer’s disease which is the finding of plaques and tangles on pathologies. (C-GDDI-Ex1)  And I guess for… we should call it AD dementia just for semantics (C-Ex1)  That’s important. (C-Method)  You guys are arguing about AD versus ADD? (C-Method)  Yeah I know what we have to do… possible ADD though references to McKann criteria (C-GDDI-Ex1) |
| Accuracy of clinical Alzheimer disease diagnosis | So if you look at numbers across various autopsy based studies our sensitivity and specificity for AD clinically is on the order of 50-90% depending on the center for sensitivity and you know (P-Ex2)  You know regular diagnosis in the community, that’s on that 50% spectrum. When you look dedicated memory centers that’s where you’re getting up to that 90-95%. (P-Ex1)  Yeah I disagree actually I think that much of that data is based on autopsy based studies. I you look at um, you know some of that is driven a little bit, our sensitivity gets a lot better at tertiary care centers partly because our specificity  gets a lot worse, we tend to call everyone Alzheimer’s Disease at our center but if you look at numbers…and then if you look in clinical trials at amyloid positivity for mild Alzheimer’s Disease it’s on the order of you know 80%, 85% certainly for non ApoE for carriers so there is a fair bit of uncertainty and then you get into the mild cognitive impairment range where our amyloid positive raid even studies like ADNI which are very well vetted and very um, you know all of them are specialty centers for the most part and it’s about you know anywhere from 45-60% are amyloid positive and have mild cognitive impairment range and if you use that again as a gold standard I would argue we have fairly poor clinical acumen for… (P-Ex2)  Well I’m looking at this very simplistically, um, and I thank you for your incredible questions, but in my small experience with this I am hoping that one of the outcomes of the amyloid testing will be that the diagnosis of the disease will be much more respected with doctors. Because as it stands now we hear different…differences in numbers where 33% of those really who have Alzheimer’s or dementias are actually diagnosed, 46%, but it’s all below 50% and that’s what we keep hearing over and over again and the rationale (P-CP2) | In terms of the setting, I mean you’re going to probably have a different facility or abilities to make diagnosis or detect things in those three settings and so you’ll probably have different populations that would then could be potentially tested. (C-GDDI4)  So yeah I think there’s…if I’m not mistaken, there’s data that suggests that you know uh a cognitive neurology experts have a really high (inaudible) sort of concordance with brain pathology with clinical…like something in the 90s (C-Mod)  But it’s also positive predictive value with that assessment by walking into my clinic by definition my patients have an 80% chance of having Alzheimer’s disease because they’re saying I’ve got memory complaints and they are referred to me by people who have evaluated for other (C-GDDI-Ex1)  [The percentage of patients with what seems to be clinically typical Alzheimer’s disease without amyloid present on imaging in] the solanezumab trial [was] 20%, over 20? (C-Ex1)  Those are probably bias by people, clinicans maybe trying to get their patients into this study [C-GDDI-Ex1] |
| Accuracy of MCI diagnosis | MCI is just a clinical syndrome and we know that it’s going to develop in various different ways. There is some of those patients that will develop into Alzheimer’s and if it’s amnestic the majority have that potential but some of those people have underlying Tau pathologies, they have vascular changes, some of those are going to develop Lewy Body or Parkinson’s disease dementia I mean it’s…MCI is a pre-dementia syndrome it is not a pathology diagnosis so it’s OK that only 40-50% have…unless you think…now if we’re talking about going with the new diagnostic criteria for pre-clinical Alzheimer’s or you know this in between, that’s where I think it becomes problematic. Identifying which percentage of…or what portion of that MCI old category is actually Alzheimer’s pathology, pre-dementia. (P-Ex1)  So let me just clarify the two kind of experts, so would you agree then that among patients who come in with MCI, even a specialist is not very good at determining who would have amyloid pathology at that time. Would amnestic MCI can you say with some certainty there’s like an 80% likelihood based on exam that patient has amyloid pathology or is it much worse than that. (P-Mod)  It’s lowered. I mean if again if you look in ADNI, which you could take as a cohort again which is very much driven by um, tertiary care, memory specialty centers, you know and depending on the severity of MCI the range is you know in the 45-60%, 65% range for amyloid positivity. You take community samples of mild cognitive impairment, it’s even lower than that just given the expertise of the community. (P-Ex2) | And people with MCI you’ll have a lot, 40% are amyloid (talk over) (C-Ex1) |
| Amyloid angiopathy [both groups indicate no need to include this specific diagnostic group in guideline] | I mean there are…I mean we…there are abnormalities the PET scans in the context of cerebral angiopathy, um, there’s some argument about the differences in the pattern you see in the context of Alzheimer’s disease um, pathology in the absence of cerebral amyloid angiopathy versus with it. I don’t’ know if there’s actually good data on people who have more isolated cerebral amyloid angiopathy um, how sensitive amyloid imaging is to looking just more secure, um, but mostly those cases are often mixed and so it is um, something (P-Ex2)  There’s not…I mean there’s not a great data that you can distinguish between the two. I would argue that…this is my intuition…this is opinion not uh data driven that MR imaging is a much better test of amyloid angiopathy (P-Ex2)  Looking at those micro-hemorrhages (P-Ex1)  …you can detect sort of tiny things you would never even know that you had associated with it. Probably my guess would be if you don’t have those micro hemorrhages M1 angiopathy your risk would be...your risk might not be that high anyway. Although that’s sort of my memory. (P-Ex2) | Well in general [amyloid angiopathy] coexist[s] with Alzheimer’s disease, but yes you can have someone with just amyloid angiopathy and without…that will be positive that doesn’t have dementia (C-Ex1)  They would have an MRI showing multiple microbleeds (C-Ex1)  And I guess the question is in that patient do we even need to send them for an amyloid PET scan when you look at their SWI and you see multiple lobar (talk over) (C-GDDI-Ex1)  [Discussion that MRI with SWI/GRE part of all dementia protocols but not good quote]  So if you have someone with vascular dementia, uh and you think it’s amyloid angiopathy, the amyloid PET would add nothing to your clarification. (C-GDDI4)  No in the new ICH guidelines they actually talk about getting APOE testing for prognostication of future hemorrhage risk. Um and a better test would actually be…if you have a lobar hemorrhage and say and you’re deciding on anti-platelets versus warfarin in someone…You can stratify them based on their APOE status. The idea that someone with an APOE 4 or 2 allele would be a much higher risk and you should try and avoid anti-platelet, anti-coagulants because their risk is going to be so high for future (talk over) (C-Ex1) |
| Details of actual scanning |  | Actually I want to back off just to the nuts and bolts, so to do the amyloid PET scan is it like a 45 minute integration period? And does it matter what they are doing in terms of…during that wait time before you put them in the scanner? (C-GDDI4)  You mean the scanning? It takes like 20 minutes. (C-Ex1)  No, no when I was doing FDG PET you gave them the tracer, they did something for 45 minutes and then you put them in the scanner. (C-GDDI4)  No it’s not that long, I don’t know how long (C-Ex1)  I don’t know the exact time (inaudible) but it’s very reasonable. And there’s no activity requirements I mean they don’t have to fast that you will with the FDG-PET, but it’s very well tolerated. I think the actual, I’m sure we’ll review the harms of the test and the risks of the test but those are well established through (inaudible) process. (C-GDDI-Ex1) |
| Details of interpretation of PET |  | The PET scan is detecting we think pathology (C-GDDI-Ex1)  So is the PET scan rated as negative or positive? (C-GDDI4)  In clinical practice that’s the way it’s done, positive negative, um, but for research they have a…they take certain regions that have a high amyloid load and come up with a number quantification you know for positive and negative. But for clinical purposes I think for this it would be positive or negative. (C-Ex1)  When you’re using it in a clinic now, yeah like I said for research you can do a quantified number that changes over time, but if you ordered it clinically if would be read as positive or negative. (C-Ex1)  Does the clinical definition of positive have…is it a standard definition like it has to have a certain quantity to be positive or is it subject to interpretation of the radiologist or (talk over) (C-GDDI2)  The nuclear medicine societies have kind of come up with a training program to be able to grade these and then they have good intra rate of reliability for when someone is calling something positive. (C-Ex1)  So it has to have a certain threshold of amyloid quantity basically to be considered positive. (C-GDDI3)  Essentially (C-Ex1) |
| Tracer differences |  | Are the three tracers equivalent? (C-GDDI-Ex2)  Uh that’s a good question, uh probably not. There’s just small studies to guide us but I think (C-Ex1)  No I think that’s the right answer (C-GDDI-Ex1)  Florbetapir, Amyvid, is the best study and uh, pretty reliable but otherwise there’s smaller studies, autopsy conformed groups and their sensitivity and specificity were slightly lower. But there’s not been a head to head that I know of. (C-Ex1) |
| FTD presentations without cognitive symptoms |  | I have a different standard when I do memory disorder clinic. If the patient had made the appointment, had driven themselves, you know if they were on time they were sitting in the waiting room I would shake their hand and say them there’s nothing wrong with them. (C-Method)  Yeah well you don’t see a lot of FTD patients then because they do all those things as well. (C-GDDI-Ex1)  Really they do all those executive things? (C-Method)  Yeah – it can be a fully behavioral presentation (C-Ex1)  Interesting (C-Method) |
