# Supplementary material for: Impact of patient involvement on clinical practice guideline development: a parallel group study
Source: Implement Sci. 2018 Apr 16;13:55. doi: 10.1186/s13012-018-0745-6 (PMC5902835; doi:10.1186/s13012-018-0745-6)
Supplement: Supplementary file 4 — PICOT questions from experimental (Gp1) and control (Gp2) groups. Microsoft Word table of proposed guideline PICOT questions. (DOCX 17 kb) [file 13012_2018_745_MOESM4_ESM.docx]

**Additional file 4. PICOT questions from Experimental (Gp1) and Control (Gp2) Groups**

|  | Gp 1 Q1 | Gp 1 Q2 | Gp 1 Q3 (diagnosis) | Gp 1 Q4 (prognosis) |
| --- | --- | --- | --- | --- |
| P | In patients at risk for AD without CI (preclinical), | In patients with MCI suspected to be prodromal AD, | In patients with dementia, | In patients with dementia, |
| I | Does beta-amyloid imaging | Does beta-amyloid imaging | Does beta-amyloid imaging | Does beta-amyloid imaging |
| C | Compared to not getting beta-amyloid imaging | Compared to not getting beta-amyloid imaging | Compared to not getting beta-amyloid imaging | Compared to not getting beta-amyloid imaging |
| O | Accurately predict developing cognitive impairment due to Alzheimer’s pathology | Accurately predict developing dementia due to Alzheimer’s pathology | Improve accuracy of diagnosing Alzheimer’s disease dementia? | Accurately predict rate of decline due to Alzheimer’s dementia? |
| T | In 1, 3, 5, 10, and 15 years? | In 1, 3, 5, 10, and 15 years? |  |  |

|  | Gp 1 Q5 (prognosis) | Gp 1 Q6 (diagnosis) | G2 Q2 (diagnostic accuracy) | G2 Q3 (diagnostic accuracy) |
| --- | --- | --- | --- | --- |
| P | In patients with MCI, | In patients with dementia, | For persons at present or future risk of ADD (1. asymptomatic, 2. subjective symptoms without objective findings, 3. MCI, 4. typical dementia [AD], 5. atypical dementia [AD], | For persons at present or future risk of ADD (1. asymptomatic, 2. subjective symptoms without objective findings, 3. MCI, 4. typical dementia [AD], 5. atypical dementia [AD], |
| I | Does beta-amyloid imaging | Does beta-amyloid imaging | Does amyloid PET | Does amyloid PET in addition to “standard evaluation” (standard clinical, radiographic, CSF evaluation) |
| C | Compared to other testing (see list; head-to-head comparisons only) | Compared to other testing (see list; head-to-head comparisons only) | As compared to a reference standard (see list) | As compared to a standard evaluation alone |
| O | Better predict progression to Alzheimer’s dementia? | Better determine the type of dementia? | Accurately identify patients with ADD as determined by an independent reference standard (the outcome here will be sensitivities, specificities and for non-case control studies predictive values) | Increase the accuracy of identifying patients with ADD as determined by an independent reference standard (outcome: comparison of diagnostic accuracy measures) |

|  | Gp 1 Q7 (prognosis) | Gp 1 Q8 | Gp 2 Q1 (diagnostic utility question) | Gp 2 Q4 (specificity question, screening criteria) |
| --- | --- | --- | --- | --- |
| P | In patients with dementia, | In patients at risk for AD without CI (preclinical), | For persons at present or future risk of ADD (1. asymptomatic, 2. subjective symptoms without objective findings, 3. MCI, 4. typical dementia [AD], 5. atypical dementia [AD], | For persons without ADD |
| I | Does beta-amyloid imaging | Does beta-amyloid imaging | Does undergoing amyloid PET (any tracer) | How often does amyloid PET |
| C | Compared to other testing (see list; head-to-head comparisons only) | As compared to other testing (see list; head-to-head comparisons only) | Compared to not undergoing amyloid PET | (As compared to how often it would be indentified without amyloid PET, which is zero by definition) |
| O | Better predict rate of progression? | Better predict progression to Alzheimer’s dementia? | Improve outcomes:  - Quality of life  - Overall costs  - Other outcomes (benefits/harms of “correct” diagnosis, stopping search for other causes, treatment, preparation for personal consequences of ADD, preparation for social consequences of ADD) | Demonstrate amyloid? (Proportion of persons with amyloid) |

Gp: Group, AD: Alzheimer disease, MCI: mild cognitive impairment, ADD: Alzheimer disease dementia, PET: positron emission tomography, CSF: cerebrospinal fluid
